# Eating disorder risk during behavioral weight management in adults with overweight or obesity: A systematic review with meta‐analysis

**DOI:** 10.1111/obr.13561

**Published:** 2023-03-15

**Authors:** Hiba Jebeile, Sol Libesman, Hannah Melville, Timothy Low‐wah, Genevieve Dammery, Anna L. Seidler, Rebecca A. Jones, Caitlin M. McMaster, Susan J. Paxton, Andrew J. Hill, Amy L. Ahern, Sarah P. Garnett, Caroline Braet, Denise E. Wilfley, Louise A. Baur, Natalie B. Lister

**Affiliations:** ^1^ Children's Hospital Westmead Clinical School The University of Sydney Sydney New South Wales Australia; ^2^ NHMRC Clinical Trials Centre The University of Sydney Sydney New South Wales Australia; ^3^ InsideOut Institute for Eating Disorders The University of Sydney Sydney New South Wales Australia; ^4^ MRC Epidemiology Unit University of Cambridge Cambridge CB2 0QQ UK; ^5^ School of Psychology and Public Health La Trobe University Melbourne Victoria Australia; ^6^ Leeds Institute of Health Sciences University of Leeds Leeds UK; ^7^ Kids Research The Children's Hospital at Westmead Westmead New South Wales Australia; ^8^ Department of Developmental, Personality and Social Psychology Ghent University Henri Dunantlaan 2 Ghent 9000 Belgium; ^9^ School of Medicine, Washington University in St. Louis Missouri St. Louis USA

**Keywords:** diet intervention, disordered eating, treatment

## Abstract

This systematic review examined change in eating disorder risk during weight management interventions. Four databases and clinical trials registries were searched in March and May 2022, respectively, to identify behavioral weight management intervention trials in adults with overweight/obesity measuring eating disorder symptoms at pre‐ and post‐intervention or follow‐up. Random effects meta‐analyses were conducted examining within group change in risk. Of 12,023 screened, 49 were eligible (*n* = 6337, mean age range 22.1 to 59.9 years, mean (SD) 81(20.4)% female). Interventions ranged from 4 weeks to 18 months, with follow‐up of 10 weeks to 36 months post‐intervention. There was a within group reduction in global eating disorder scores (20 intervention arms; Hedges' *g* = −0.27; 95% CI −0.36, −0.17; *I* ^2^ 67.1%) and binge eating (49 intervention arms; −0.66; 95% CI −0.76, −0.56; *I* ^2^ 82.7%) post‐intervention, both maintained at follow‐up. Of 14 studies reporting prevalence or episodes of binge eating, all reported a reduction. Four studies reported eating disorder symptoms, not present at baseline, in a subset of participants (0%–6.5%). Overall, behavioral weight management interventions do not increase eating disorder symptoms for most adults; indeed, a modest reduction is seen post‐intervention and follow‐up. A small subset of participants may experience disordered eating; therefore, monitoring for the emergence of symptoms is important.

**Abbreviations**
BES: Binge Eating Scale
EDE‐Q: Eating Disorder Examination Questionnaire

## 1 INTRODUCTION

Behavioral weight management interventions, characterized by a multidisciplinary focus on dietary change, increased physical activity, and behavior change strategies, are currently the first‐line treatment for overweight and obesity.^1^ Previous syntheses of results from trials of these interventions have primarily focused on measures of effectiveness, such as change in weight and cardiometabolic outcomes.^2^ However, despite psychosocial and mental health being increasingly recognized as important outcomes of obesity care,^3^ eating disorder risk remains understudied from an efficacy or safety perspective. Evidence shows that the prevalence of eating disorders is higher in adults with overweight or obesity, compared to those of lower weight,^4^ and there is a potential lifelong comorbidity associated with eating disorders.^5^ This amplifies the need to investigate the impact of behavioral weight management interventions on eating disorder risk.

Behavioral weight management interventions aim to improve weight status and health outcomes for adults with overweight or obesity. However, there are concerns that interventions may unintentionally contribute to the onset of disordered eating or the development of eating disorders.^6^, ^7^ Several core components of behavioral weight management interventions are considered risk factors or behaviors associated with the development of eating disorders in community samples. For example, although dieting and dietary restraint are frequently employed in weight management interventions, they are also associated with the onset of binge eating, binge eating disorder, and bulimia nervosa in adolescent girls.^8^, ^9^ Similarly, excessive dietary restriction, excessive increase in physical activity, and significant and/or rapid weight loss are features of anorexia nervosa.^10^ Our systematic review of dietary interventions used for weight management in children and adolescents found that eating disorder risk did not increase,^11^ and that dietary restraint may not be a useful marker of risk in this context.^12^ However, to our knowledge, there is a lack of understanding on the impact of weight management interventions on eating disorder risk in an adult population. Behavioral weight management interventions conducted in adults may have a greater focus on weight loss and may use more prescriptive interventions, compared to those used in pediatrics, warranting investigation.

Recent systematic reviews have examined the impact of weight management interventions on various dimensions of mental health and disordered eating among adults. A 2020 meta‐analysis of 42 trials found behavioral weight management interventions to lead to a greater improvement in symptoms of depression, mental health‐related quality of life, and self‐efficacy than inactive comparators.^13^ This review identified only one study reporting results for binge eating, finding no difference between intervention and control arms on the likelihood of reporting any binge eating.^13^, ^14^ A 2017 systematic review identified five trials of weight management interventions measuring eating disorder risk.^15^ All trials reported improved eating disorder outcomes, including a reduction in binge eating post‐intervention compared to baseline. A 2015 systematic review of 10 trials^16^ examined restrictive diets including low‐energy diets and very low energy diets with mixed findings. Trials including participants with pre‐treatment binge eating disorder generally reported a reduction in binge eating behaviors. Yet, among trials including participants with sub‐clinical or no binge eating symptoms at baseline, some reported a reduction in symptoms and others reported no change or an increase. Within that review, two studies reported the onset of binge eating or binge eating disorder in 10%–15% of participants who did not report binge eating at baseline.^16^

The conflicting findings across previous relevant reviews make it difficult to thoroughly understand the impact of behavioral weight management interventions on a broad range of eating disorder outcomes. Additionally, the present literature has either had a broad focus, that is, including all mental health outcomes^13^ or all obesity treatment approaches and outcomes,^15^ or a very narrow focus, that is, limited to examining binge eating following restrictive diets.^16^ To date, there has not been a comprehensive synthesis focused on eating disorder outcomes following behavioral weight management in adults, and no review of eating disorder risk in weight management has included a meta‐analysis.

Eating disorder risk may be measured using a variety of assessments examining different outcomes. This ranges from risk scores for global eating disorder risk or binge eating severity, for example, using the Eating Disorder Examination Questionnaire (EDE‐Q)^17^ or the Binge Eating Scale (BES),^18^ to the number of episodes of disordered eating behaviors (e.g., binge eating, loss of control, or purging) and/or the prevalence of various disordered eating behaviors or symptoms. To comprehensively investigate the impact of behavioral weight management on eating disorder risk, this systematic review aimed to examine the change in a broad range of eating disorder outcomes, including prevalence, global eating disorder scores, binge eating scores, and episodes.

## 2 METHODS

This systematic review was prospectively registered on PROSPERO (CRD42021265340) and follows the Preferred Reporting Items for Systematic Reviews and Meta‐Analysis (PRISMA).^19^

### 2.1 Eligibility criteria

This systematic review included randomized controlled trials of behavioral weight management interventions conducted in adults (aged ≥ 18 years at baseline) with overweight or obesity, defined as body mass index (BMI) ≥ 25 kg/m^2^. Interventions were targeted to individuals or groups. Trials aimed at obesity prevention in a community sample and including individuals in a healthy weight range were excluded, but obesity prevention trials in a population classified as having overweight were included. Trials were excluded if they included bariatric surgery or pharmacotherapy, or targeted secondary or syndromic causes of obesity (e.g., Prader Willi syndrome) or an alternate medical condition (e.g., type 2 diabetes and sleep apnea), or if they aimed to treat eating disorders. Trials comparing two or more active interventions, for example, a novel intervention compared to standard care, as well as those comparing a weight management intervention to a no‐treatment control were eligible. Intervention arms were defined as those providing any advice or information relating to nutrition, physical activity, sedentary behavior, sleep health, or behavior change outcomes, for the purposes of weight management, including print information, online programs, or individual or group consultations. The intervention duration was defined as any period with ongoing contact with the study team, and follow‐up was defined as the period with no contact or intervention provided. Support provided during a weight maintenance period was considered part of the intervention. Control arms were defined as those providing no treatment or support during the study period, for example, waitlist control groups. There was no limit on intervention duration, setting (e.g., community, inpatient, and outpatient), date, or language. Articles in a language other than English were translated using Google Translate to assess eligibility.

### 2.2 Outcomes

Trials were required to report one or more measures of eating disorder risk, symptoms, or behaviors at baseline and post‐intervention or follow‐up using a validated self‐report questionnaire or diagnostic interview, for example, clinical diagnosis (e.g., using the Eating Disorder Examination [EDE] interview), global risk score or binge eating episodes (e.g., using the EDE‐Q), and binge eating severity (e.g., using BES).

### 2.3 Information sources and search strategy

**FIGURE 1:**
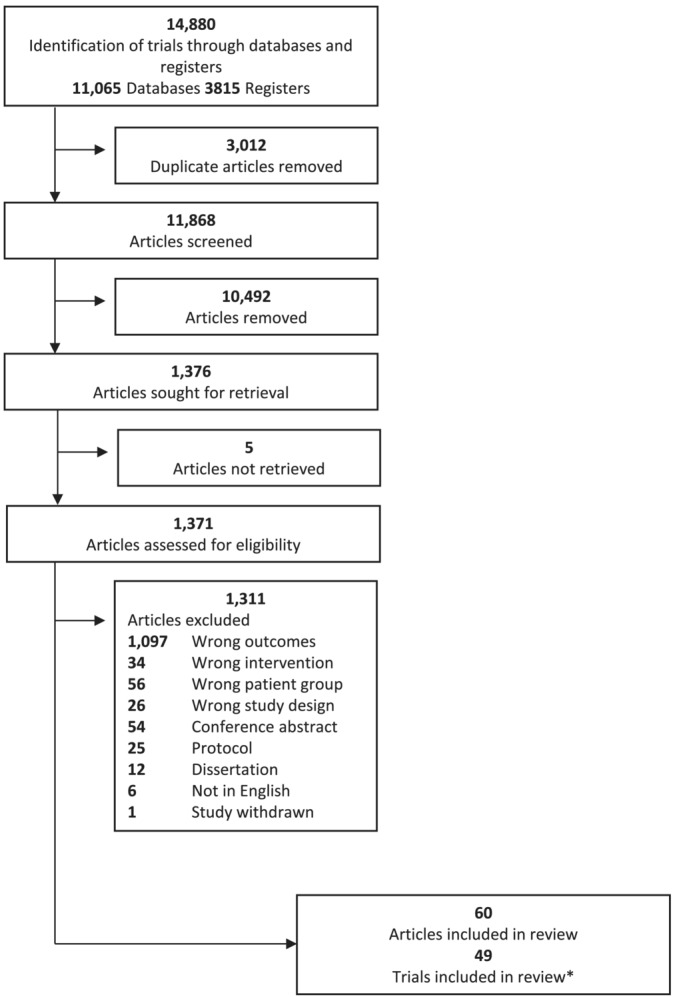
Preferred Reporting Items for Systematic Reviews and Meta‐Analysis (PRISMA) flow diagram. *Two articles each were identified for 11 trials.

Electronic databases (MEDLINE, Embase, PsycINFO, and Scopus) were searched from inception to March 2022 (see Table S1 for search strategies). Records were imported into Covidence systematic review software (Veritas Health Innovation, Melbourne, Australia) to remove duplicates and screen against eligibility criteria. Two reviewers independently screened records by title and abstract and then full text, with reasons for full text exclusion recorded (Figure 1). The clinical trials registries^20^ ClinicalTrial.gov and WHO International Clinical Trials Registry Platform were searched from inception to May 2022 using the key words weight management OR obesity treatment. Records were exported to Microsoft Excel (Microsoft Corporation, WA, USA) and first screened by title and outcomes by one reviewer, and then the complete online records were screened by two reviewers. Conflicts were resolved through discussion.

### 2.4 Data extraction

Data were extracted independently by one reviewer and checked for accuracy by a second. Extracted data included participant characteristics, study and intervention design, setting, frequency of contact, eating disorder assessment tool, and weight‐related (e.g., weight and/or BMI) and eating disorder outcomes at all measured timepoints. Where the required data for meta‐analysis were not reported, authors were contacted to request this. One study was excluded from meta‐analysis because the required data could not be obtained.

### 2.5 Risk of bias assessment

Risk of bias was assessed independently by two reviewers with discrepancies resolved through discussion. Each intervention arm within a trial was assessed independently, using the US Academy of Nutrition and Dietetics Quality Criteria Checklist: Primary Research.^21^ The checklist allows a rating of positive, neutral, or negative to be given to each intervention arm within the study.

### 2.6 Data synthesis

First, a narrative synthesis of results was conducted according to the Synthesis Without Meta‐analysis (SWIM) guidelines.^22^ Trials were synthesized as those comparing a weight management intervention with a no‐treatment or weight–neutral comparator and trials comparing two or more weight management intervention arms. As the focus of this review was to examine eating disorder risk from a safety perspective, we primarily examined the pre‐post intervention change within each intervention arm and the difference between weight management interventions and controls when the comparator did not provide a weight management intervention, that is, waitlist or weight neutral control groups.

Where the required data were available, meta‐analyses were conducted to determine the difference in means between baseline and post‐intervention, post‐intervention and follow‐up, and baseline and follow‐up for global eating disorder and binge eating scores. Post‐intervention was defined as the outcome measure taken immediately following the end of the intervention. Follow‐up was defined as the latest data collection timepoint, following a period of no intervention. Meta‐analyses were conducted to examine the within group change within individual intervention arms and the difference between intervention arms and weight‐list controls where more than two studies were available. Analyses were conducted using the *metafor* package^23^ (version 3.0–2) for R^24^ (version 4.1.3), and the results are presented in the form of forest plots.

For analyses that required the combination of scores from different questionnaires, Hedges' *g* and the respective 95% confidence interval (95% CI) were calculated by applying the method recommended by Goulet–Pelletier (2018)^25^ for estimating the effect size between two repeated measurements. Hedges' *g* was chosen as the measure of effect size as it carries the same interpretation as Cohen's d (0.2 = small effect, 0.5 medium effect and 0.8 large effect), and it is not biased when applied to small samples. We used the pooled standard deviation as the standardizer for Hedges' *g*, as has been recommended.^25^, ^26^ When the correlations between timepoints were not available, we estimated Hedges' *g* through using a conservative estimate of *r* = 0.7 according to the recommendation of Rosenthal (1993).^27^ In the small number of trials where only the standard deviation of the difference was reported, we estimated the pooled standard deviation assuming the same correlation value. We probed how robust our conclusions were to our assumptions in sensitivity analyses which varied the assumed correlation values [*r* = 0.3, 0.5, 0.9]. To display any differences between trials that required the use of a correlation assumption to calculate the pooled standard deviation, we reported the subgroups for trials that had reported scores at each time point (no correlation assumption) against the small number of trials that only “reported difference scores.” When scores were synthesized across the same questionnaire (EDE‐Q only or BES only), the effect measures used were mean difference scores and the respective 95% confidence interval.

We used a random effects meta‐analysis model to synthesize all estimates as the trials being examined employed varying interventions and measurement tools. This was deemed appropriate as random effects models assume there are true effect estimates that vary between studies and that this is because of heterogeneous factors. Heterogeneity was examined through *τ* ^2^ which captures the between study variance and prediction intervals which captures the likely effect sizes of a new study if that study was selected at random from the same population of studies. Inconsistency between observed variance and heterogeneity was captured with *I* ^2^. Heterogeneity was further explored by examining whether the time of data collection was related to the effect estimate using meta‐regression in each respective analysis. Where sufficient data were available, sub‐group analyses were conducted to compare active intervention arms with standard care/minimal interventions (e.g., information booklet). Publication bias was examined by funnel plots and Egger's test.

## 3 RESULTS

### 3.1 Included studies

From 12,023 articles screened, 49 trials met eligibility criteria for this review (Figure 1). Characteristics of included trials are summarized in Table S2. Included trials were published between 1994 and 2022 and were conducted in the United States (combined sample size, *n* = 4016), United Kingdom (*n* = 557), Australia (*n* = 412), the Netherlands (*n* = 336), Italy (*n* = 251), New Zealand (*n* = 250), Canada (*n* = 195), Finland (*n* = 167), Brazil (*n* = 74), and Greece (*n* = 34). Sample size ranged from 30 to 572 participants per trial, mean (SD) 81 (20.4)% female, with a mean age at baseline ranging from 22.1 to 59.9 years, and mean BMI range from 27.0 to 47.9 kg/m^2^.

#### 3.1.1 Intervention characteristics

Most trials compared two or more weight management intervention groups,^28^, ^29^, ^30^, ^31^, ^32^, ^33^, ^34^, ^35^, ^36^, ^37^, ^38^, ^39^, ^40^, ^41^, ^42^, ^43^, ^44^, ^45^, ^46^, ^47^, ^48^, ^49^, ^50^, ^51^, ^52^, ^53^, ^54^, ^55^, ^56^, ^57^, ^58^, ^59^, ^60^, ^61^, ^62^, ^63^, ^64^, ^65^, ^66^, ^67^, ^68^, ^69^ and five compared a weight management to a weight–neutral intervention group using a non‐diet approach to health focusing on self‐acceptance and improving emotional well‐being.^70^, ^71^, ^72^, ^73^ Four trials included a no‐treatment or waitlist comparator group.^14^, ^71^, ^73^, ^74^ Interventions were primarily conducted in community or hospital outpatient settings, with one trial conducted as part of care provision within the US military. Intervention duration ranged from 4 weeks to 18 months with follow‐up of 10 weeks to 36 months from the end of intervention. Interventions were delivered by multi‐disciplinary teams or a single health professional. Two trials^34^, ^36^ did not report who delivered the intervention.

Dietary components within interventions included nutrition education with or without an energy prescription (ranging 1200 kcal/day to 2000 kcal/day) with some trials including additional approaches such as mindful eating.^35^, ^53^, ^57^, ^59^, ^62^, ^63^ Two trials^61^, ^75^ used a very low energy diet (420 to 1000 kcal/day) within one intervention arm. Physical activity interventions ranged from education to increase activity and reduce sedentary time to providing a structured and supervised exercise program. Behavior change, psychological, or counselling strategies within interventions ranged from guided self‐help to group and/or individual counselling sessions with a clinical psychologist. Cognitive behavior therapy was provided as part of the intervention for six studies.^38^, ^39^, ^51^, ^56^, ^65^, ^67^

#### 3.1.2 Outcomes

Studies measured eating disorder risk using nine different assessment tools. The EDE‐Q was most frequently used to assess global eating disorder risk and/or objective or subjective binge eating episodes, and the BES was most frequently used to measure binge eating severity. Across all included assessments, a higher score indicates greater symptom severity. Outcome data for all included trials can be found in Tables S2 and S3.

### 3.2 Risk of bias assessment

No differences in quality ratings were assigned for intervention arms within a trial; an overall rating per trial is reported in Table S2. A positive quality rating was assigned to 31 studies, and the remaining 18 studies assessed were of neutral quality. No studies were given a negative quality rating. The most frequent reasons for a neutral evaluation were because of not reporting method of randomization or blinding of outcome assessments.

### 3.3 Trials with intervention versus no‐treatment or weight–neutral comparator

#### 3.3.1 Change in global eating disorder risk score

Two trials reported reductions in some subscales on the Eating Disorder Inventory (EDI)^76^ in the weight–neutral group^73^ or in both the intervention and weight–neutral groups. A greater reduction was reported in the weight–neutral group on the drive for thinness and maturity fears subscales.^70^ One trial^14^ reported no difference between groups on the Anorectic Cognitions Scale, and another^72^ found a greater reduction on the EDE‐Q post‐intervention in the weight–neutral group compared to the weight management intervention group, with no difference between groups at 24 months follow‐up. On the subscales for the EDE‐Q, there was a reduction in both groups on the shape and weight concern subscales, with no difference between groups. There was a significant difference in the dietary restraint subscale with an increase in the weight management group and no change in the weight–neutral group.^72^

#### 3.3.2 Change in binge eating score

One trial^71^ reported a reduction in binge eating scores in both the intervention and weight–neutral groups, with no difference between groups. Intervention groups had a greater reduction in binge eating scores than the no‐treatment control groups in two trials.^71^, ^74^

#### 3.3.3 Change in binge eating behaviors

One trial^14^ reported a statistically significant reduction in the number of participants reporting binge eating episodes in the intervention group from 30% (*n* = 14) at baseline to 14% (*n* = 6) at 6 months and no change in the control group (*n* = 8 at baseline, *n* = 9 at 6 months), with no difference between groups. Another trial^75^ reported no change in objective or subjective binge eating in the weight management and weight–neutral intervention groups.

#### 3.3.4 Meta‐analysis

Two trials^71^, ^74^ measured binge eating and compared four different intervention arms with a no treatment control. Meta‐analysis found no difference between groups post‐intervention (Hedges' *g* = 0.71; 95% CI −0.09, 1.52; *I* ^2^ 91.4%; Figure S1). There were not enough trials to evaluate publication bias. No trials measuring global eating disorder scores had a no treatment control group, and thus, meta‐analysis was not conducted.

### 3.4 Trials comparing two or more weight management interventions

#### 3.4.1 Change in diagnosis of eating disorders

One trial^49^ reported a reduced prevalence of BED following the 18‐month intervention. From 36 participants who met criteria for BED at baseline, two still met criteria at 18 months and 34 no longer met BED criteria (94%); nine participants (6%) who did not meet criteria at baseline met criteria at 6, 12, or 18 months timepoints, although none of these participants met criteria for BED at all three timepoints.

#### 3.4.2 Change in global eating disorder risk score

Nine trials reported change in global eating disorder risk scores. Two trials reported no change in eating disorder risk within the included intervention groups.^37^, ^72^ Four trials reported a reduction in ED risk within both intervention groups with no difference between groups.^30^, ^49^, ^50^, ^62^ Three trials reported no difference between intervention groups but did not report within group change.^32^, ^40^, ^46^ No trials reported increased scores within or between groups.

Five trials reported on individual subscales within the EDE‐Q. Studies reported reduced eating concern,^43^, ^54^, ^62^, ^72^ weight concern, and shape concern^50^, ^62^, ^72^ in one or more intervention arms. One trial reported increased dietary restraint,^72^ one trial reported an increase post‐intervention, no change from baseline at 1 month follow‐up and a reduction from baseline at 6 months follow‐up,^50^ and another reported no change.^54^

#### 3.4.3 Change in binge eating score

Twenty‐eight trials reported on the change in binge eating scores. Five trials reported no change in binge eating scores within at least one intervention group,^41^, ^42^, ^65^, ^69^, ^74^ and 19 trials reported a significant reduction in binge eating scores within one or more intervention groups.^28^, ^29^, ^31^, ^33^, ^34^, ^35^, ^41^, ^42^, ^44^, ^48^, ^53^, ^55^, ^56^, ^59^, ^61^, ^64^, ^67^, ^74^ Thirteen trials reported no difference between groups,^31^, ^33^, ^34^, ^36^, ^39^, ^44^, ^45^, ^48^, ^55^, ^56^, ^63^, ^64^, ^65^ five trials reported a greater reduction in binge eating scores in one intervention compared to another,^28^, ^29^, ^35^, ^41^, ^53^, ^61^ and two trials did not report between group change.^59^, ^67^ An increase in binge eating scores was not reported for any intervention group.

#### 3.4.4 Change in binge eating episodes and compensatory behaviors

Thirteen trials reported on the number of participants reporting binge eating and/or change in the number of binge eating episodes or compensatory behaviors. Six trials reported a reduction in the number of participants binge eating,^38^, ^52^, ^56^, ^58^, ^61^, ^65^ four trials reported a reduction in the number of binge eating episodes,^47^, ^57^, ^62^, ^68^ and a further four trials^29^, ^52^, ^56^, ^61^ reported a reduction in binge eating severity with participants moving from a threshold of severe or moderate to mild or no binge eating. For example, the number of participants reporting any binge eating reduced from 19.4% to 15.9% in one trial^52^ and from 24% to 17% at 3 years in another trial.^38^ Simpson et al. (2015)^58^ reported that six of 12 participants that reported binge eating at baseline had stopped at the 12 months follow‐up, and Rieger et al. (2017)^56^ reported an increase in the number of participants reporting no binge eating from 50%–53% at baseline to 78%–81% at the 12 months follow‐up across intervention groups. Trials reported a reduction in the number of participants engaging in severe binge eating (4% to 2%;^52^ 11%–14% to 5–9%;^56^), moderate/severe binge eating (34%–35% to 4%–18%^29^), and moderate binge eating (21% to 15%;^52^ 36% to 10%–17%^56^). Zwickert et al.^65^ reported that 25 (47%) participants moved from moderate to no binge eating.

One trial^57^ reported no change in self‐induced vomiting or excessive exercise, and another^58^ reported that from five participants reporting compensatory behaviors at baseline, three ceased these and two continued post‐intervention. Two trials reported no change in the purgative subscale in any intervention arm,^64^, ^69^ and another trial reported no significant change in self‐induced vomiting, laxative use, fasting/skipping meals or excessive exercise between baseline and follow‐up.^49^

Three trials descriptively reported the onset of disordered eating behaviors. Cooper et al. (2010)^38^ reported that of 114 participants (76% of sample) reporting no binge eating at baseline, seven (6.1%) reported some binge eating at 12 months follow‐up. Simpson et al. (2015)^58^ reported that of 123 participants reporting no binge eating and 129 participants reporting no compensatory behaviors at baseline, seven (5.7%) and three (2.3%), respectively, reported the onset of behaviors at 12 months follow‐up. Pacanowski et al. (2014)^52^ reported the onset of “extreme” binge eating in one participant from 309 participants completing 24 months follow‐up.

#### 3.4.5 Meta‐analyses

##### Change in global eating disorder risk score

**FIGURE 2:**
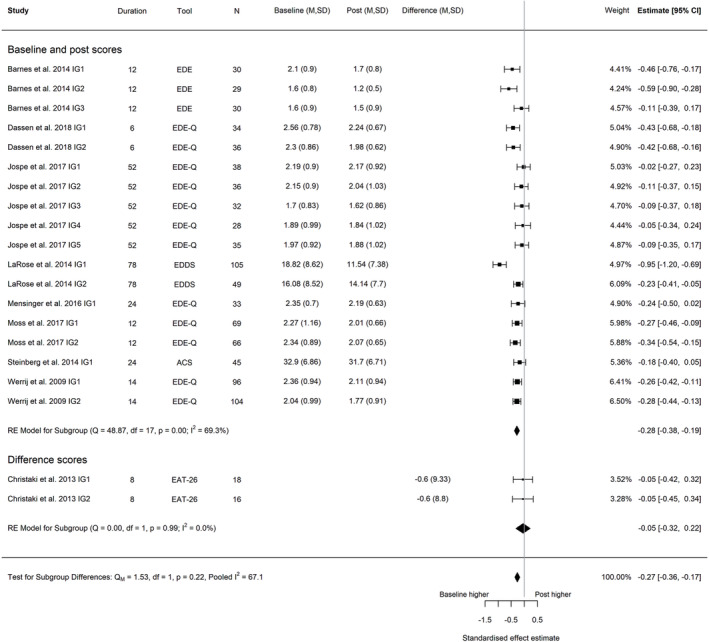
Forest plot of the change in global eating disorder scores between baseline and post‐intervention following behavioral weight management in adults with overweight and obesity. Each estimate was standardized using Hedges' *g*. A correlation of 0.7 was assumed between time points when necessary for the calculation of Hedges' *g*. A random effects model was used to combine estimates from each trial. Abbreviations: ACS, Anorectic Cognition Scale; EAT, Eating Attitudes Test; EDE‐Q, Eating Disorder Examination Questionnaire; EDE, Eating Disorder Examination, IG, intervention group.

**FIGURE 3:**
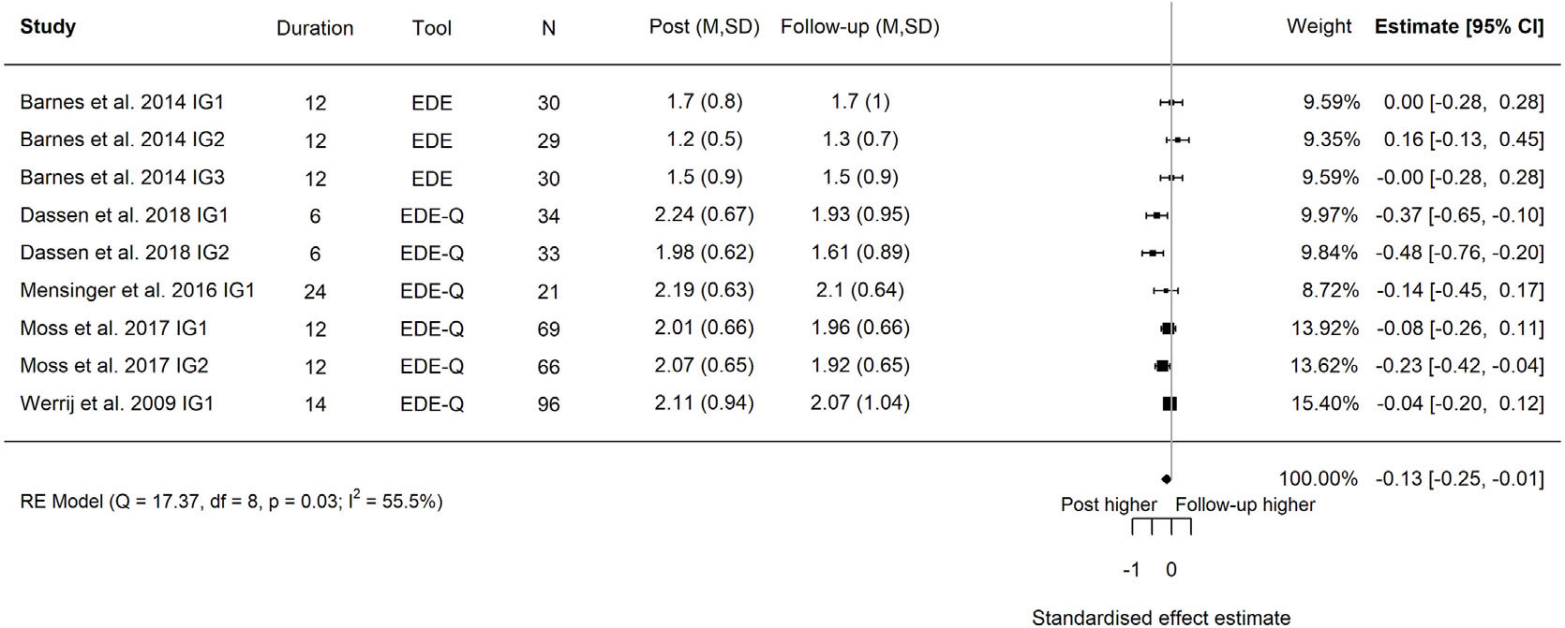
Forest plot of the change in global eating disorder scores between post‐intervention and follow‐up following behavioral weight management in adults with overweight and obesity. Each estimate was standardized using Hedges' *g*. A correlation of 0.7 was assumed between time points when necessary for the calculation of Hedges' *g*. A random effects model was used to combine estimates from each trial. Abbreviations: EDE‐Q, Eating Disorder Examination Questionnaire; EDE, Eating Disorder Examination, IG, intervention group.

**FIGURE 4:**
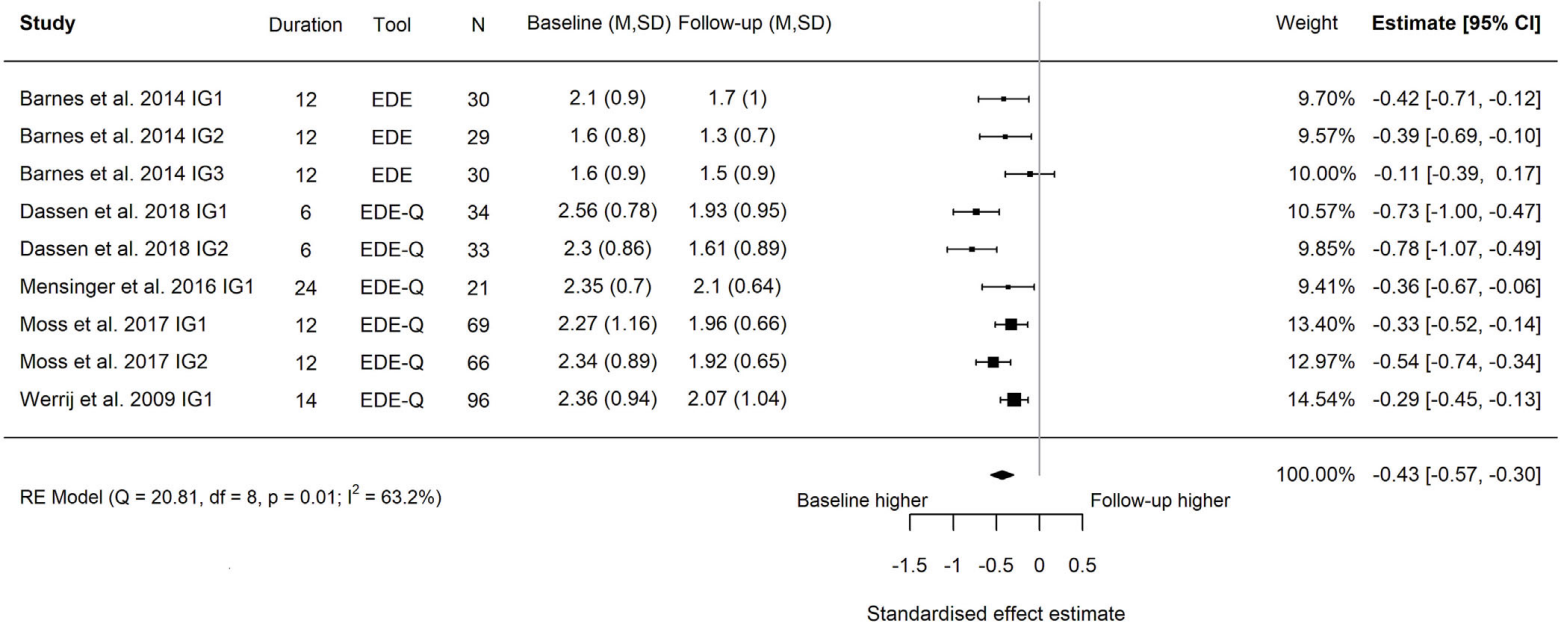
Forest plot of the change in global eating disorder scores between baseline and follow‐up following behavioral weight management in adults with overweight and obesity. Each estimate was standardized using Hedges' *g*. A correlation of 0.7 was assumed between time points when necessary for the calculation of Hedges' *g*. A random effects model was used to combine estimates from each trial. Abbreviations: EDE‐Q, Eating Disorder Examination Questionnaire; EDE, Eating Disorder Examination, IG, intervention group.

A meta‐analysis of nine trials,^14^, ^30^, ^37^, ^40^, ^46^, ^49^, ^50^, ^62^, ^72^ including 20 intervention arms and with a combined sample size of 929 participants, found a reduction in global eating disorder risk between baseline and post‐intervention (Hedges' *g* = −0.27; 95% CI −0.36, −0.17; *I* ^2^ 67.1%, 95% PI −0.62, 0.08, *τ* ^2^ = 0.029; Figure 2). The reduction in eating disorder risk was maintained at the latest follow‐up timepoint (five trials, nine intervention arms; post‐intervention to follow‐up, Hedges' *g* = −0.13; 95% CI −0.25, −0.01; *I* ^2^ 55.5%, 95% PI −0.42, 0.16, *τ* ^2^ = 0.018; Figure 3; baseline to follow‐up, Hedges' *g* = −0.43; 95% CI −0.57, −0.30; *I* ^2^ 63.2%, 95% PI −0.77, 0.09, *τ* ^2^ = 0.025; Figure 4). *I* ^2^ indicated that heterogeneity influenced a high proportion of the total variance. The prediction interval indicated that the effect size of a study selected at random from studies with a comparable population and intervention could range from a large reduction in eating disorder risk scores after intervention to a small but negligible increase in eating disorder risk. In further exploration of heterogeneity, meta‐regression revealed that the time of data collection (i.e., duration of the intervention and follow‐up period) was not associated with effect estimates between baseline and post‐intervention (*p* = 0.926), post‐intervention and follow up (*p* = 0.557), and baseline and follow‐up (*p* = 0.786) (Figures S2–S4). However, it is noteworthy that the meta‐regression for both post measurement to follow‐up, and baseline to follow‐up are comprised of less than 10 data points and fewer individual trials, and consequently, we suggest that further evidence is needed to confirm these findings. The sensitivity analyses we conducted demonstrate that these conclusions are robust to our correlation assumptions (Figures S5–S7). Only one intervention arm utilized a minimal or usual care intervention, so sub‐group analysis was not possible for global eating disorder risk.

We did not find any indication for publication bias: the funnel plot was symmetric (Figure S8–S10) and Egger's test *p*‐values 0.74, 0.7, and 0.42, respectively. Five trials^40^, ^46^, ^50^, ^62^, ^72^ using the EDE‐Q, with 12 intervention arms and a combined sample size of 607 participants, had a pooled mean reduction of 0.22 points (95% CI −0.31, −0.12; *I* ^2^ 0%) between baseline and post‐intervention (Figure S11), −0.13 points (95% CI −0.25, −0.02; *I* ^2^ 0%; four trials, six intervention arms, *n* = 319) between post‐intervention and follow‐up (Figure S12), and −0.40 points (−0.54, −0.27, *I* ^2^ 0%; four trials, six intervention arms, *n* = 319) between baseline and follow‐up (Figure S13).

##### Change in binge eating score

**FIGURE 5:**
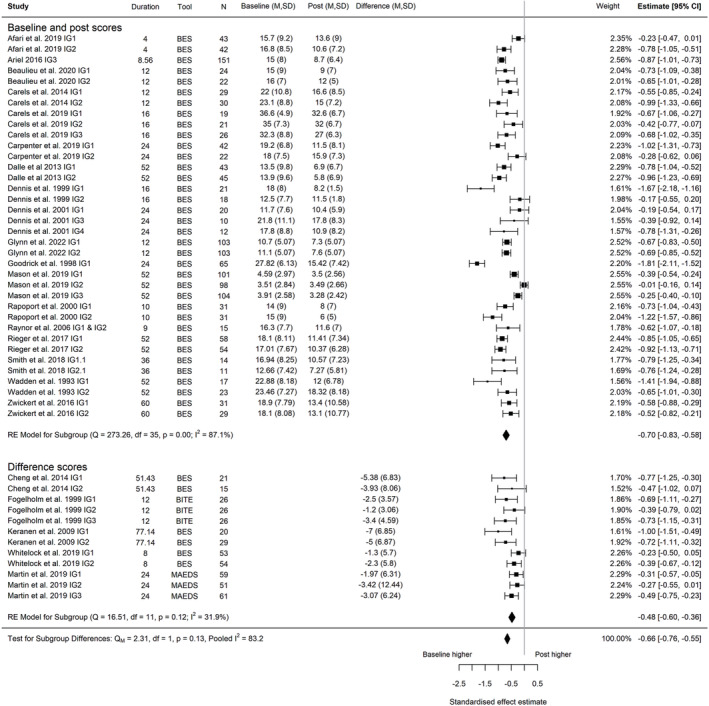
Forest plot of the change in binge eating between baseline and post‐intervention following behavioral weight management in adults with overweight and obesity. Each estimate was standardized using Hedges' *g*. A correlation of 0.7 was assumed between time points when necessary for the calculation of Hedges' *g*. A random effects model was used to combine estimates from each trial. Abbreviations: BES, Binge Eating Scale, BITE, Bulimic Investigatory Test of Edinburgh; IG, intervention group; MAEDS, The Multifactorial Assessment of Eating Disorders Symptoms.

**FIGURE 6:**
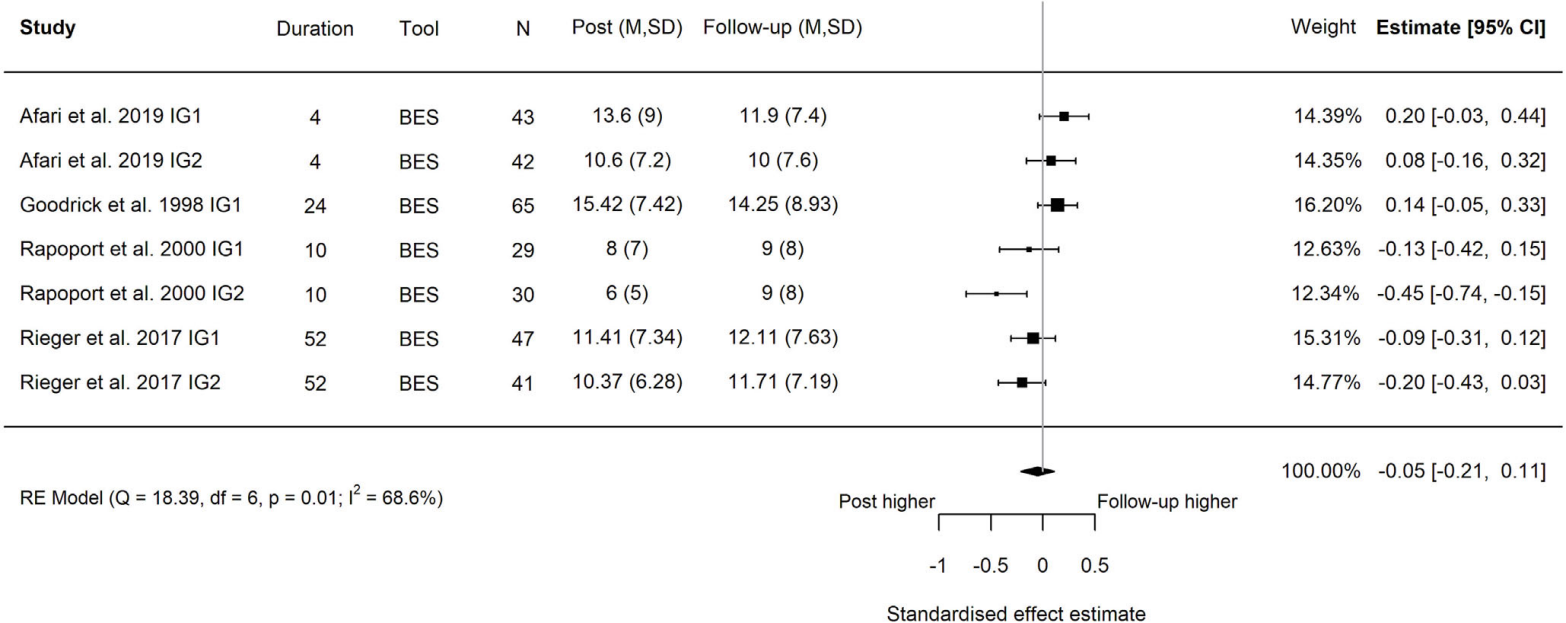
Forest plot of the change in binge eating between post‐intervention and follow‐up following behavioral weight management in adults with overweight and obesity. Each estimate was standardized using Hedges' *g*. A correlation of 0.7 was assumed between time points when necessary for the calculation of Hedges' *g*. A random effects model was used to combine estimates from each trial. Abbreviations: BES, Binge Eating Scale, IG, intervention group.

**FIGURE 7:**
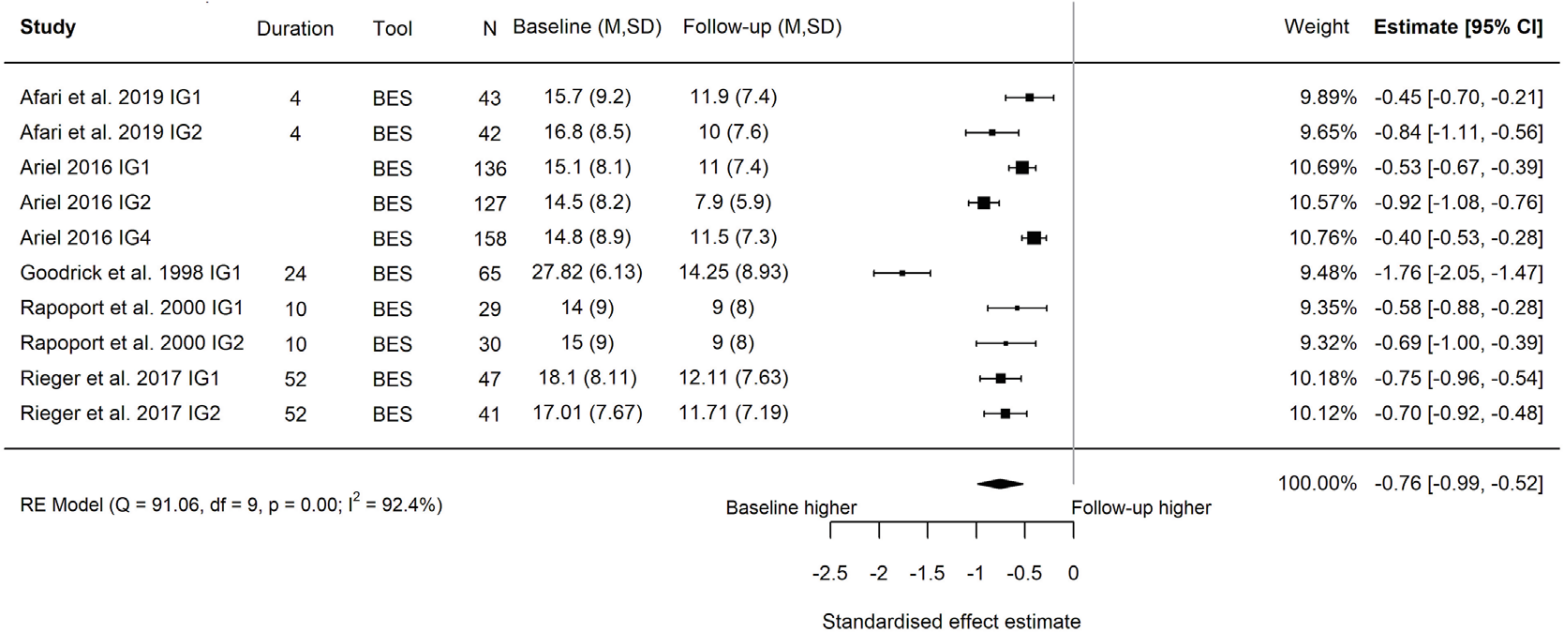
Forest plot of the change in binge eating between baseline and follow‐up following behavioral weight management in adults with overweight and obesity. Each estimate was standardized using Hedges' *g*. A correlation of 0.7 was assumed between time points when necessary for the calculation of Hedges' *g*. A random effects model was used to combine estimates from each trial. Abbreviations: BES, Binge Eating Scale, IG, intervention group.

A meta‐analysis of 23 trials,^28^, ^29^, ^31^, ^33^, ^34^, ^35^, ^36^, ^39^, ^41^, ^42^, ^44^, ^48^, ^55^, ^56^, ^59^, ^61^, ^63^, ^64^, ^65^, ^66^, ^67^, ^71^, ^74^ including 49 intervention arms and with a combined sample size of 1986 participants, found a reduction in binge eating between baseline and post‐intervention (Hedges' *g* = −0.66; 95% CI −0.76, −0.55; *I* ^2^ 83.2%; 95% PI −1.28, −0.03; *τ* ^2^ = 0.0984, Figure 5). The reduction in binge eating was maintained at the latest follow‐up timepoint (four trials, seven intervention arms; post‐intervention to follow‐up, Hedges' *g* = −0.05; 95% CI −0.21, 0.11; *I* ^2^ 68.6%; 95% PI −0.43, 0.33; *τ* ^2^ = 0.0314, Figure 6; five trials, 10 intervention arms, baseline to follow‐up, Hedges' *g* = −0.76; 95% CI −0.99, −0.52; *I* ^2^ 92.4%; 95% PI −1.49, −0.02; *τ* ^2^ = 0.1273, Figure 7). Again, *I* ^2^ indicated that heterogeneity influenced a high proportion of the total variance. The prediction interval revealed that effect sizes from studies with comparable populations and interventions could range from a large reduction in eating disorder risk score to a null effect. In further exploration of heterogeneity, meta‐regression revealed that the time of data collection was not associated with effect estimates between baseline to post‐intervention (*p* = 0.977), and baseline to follow‐up (*p* = 0.649) (Figures S14 and S15). We found a significant decrease in binge eating scores as the duration between post‐intervention and follow‐up measurement increased (*Q*_M_ = 7.16; *β* − 0.011; *p* < 0.05) (Figure S16). However, it is noteworthy that meta‐regression for both post‐measurement to follow‐up, and baseline to follow‐up are comprised of less than 10 data points and fewer individual trials, and consequently, we suggest that further evidence is needed to confirm the stability of these findings. Furthermore, the sensitivity analyses we conducted demonstrate that these conclusions are robust to our correlation assumptions (Figures S17–S19). We did not find a difference between the minimal or usual care interventions and active intervention arms for change in binge eating (*Q*_M_ = 2.31; *p* = 0.13) (Figure S20). However, as only four minimal intervention arms were included in this analysis, more evidence is needed to support these findings.

We did not find any indication of publication bias, with funnel plot symmetry (Figures S21–23) and Egger's test *p*‐value 0.06, 0.15 and 0.15, respectively. Twenty‐one trials using the BES with 43 intervention arms and a combined sample size of 1737 participants had a pooled mean reduction of 5.03 points (95% CI −5.90, −4.16; *I* ^2^ 95%; 95% PI −10.3, 0.24; *τ* ^2^ = 7.0311) between baseline and post‐intervention (Figure S24), 0.33 points (95% CI −0.84, 1.50; *I* ^2^ 66.1%; four trials, seven intervention arms, *n* = 297) between post‐intervention and follow‐up (Figure S25), and −6.05 points (95% CI −7.88, −4.22, *I* ^2^ 93.4%; five trials, 10 intervention arms, *n* = 718) between baseline and follow‐up (Figure S26).

## 4 DISCUSSION

To our knowledge, this is the first systematic review and meta‐analysis to examine the change in a comprehensive range of eating disorder outcomes following behavioral weight management interventions in adults with overweight or obesity. A reduction in global eating disorder risk, binge eating severity, and binge eating episodes (and/or no change in these) was consistently reported across intervention arms within the 49 included trials. Meta‐analyses indicated a pooled reduction in global eating disorder scores and binge eating severity sustained at the latest follow‐up timepoint. Importantly, no trials reported a mean increase in any measure of disordered eating across any intervention arm. This supports the safety of weight management interventions, for most adults, up to 36 months from post‐intervention.

Results are consistent with previous reviews examining the impacts of weight management on the mental and psychosocial health of adults with overweight or obesity,^13^, ^15^ and reviews examining eating disorder risk in weight management interventions in adults^15^, ^16^ and children and adolescents.^11^ As with these earlier reviews, reporting of long‐term eating disorder outcomes was limited, with only nine of 49 included trials reporting follow‐up from the end of intervention ranging from 10 weeks to 36 months. Further research is required to understand the implications of weight management on eating disorder symptoms and behaviors over much longer periods.

Overall, we identified few trials with a no‐treatment control arm. As such, we were unable to conduct a meta‐analysis comparing weight management interventions with no‐treatment control for global eating disorder risk, and only two trials were included in our meta‐analysis comparing intervention with no‐treatment control for the change in binge eating. Because of this limitation in currently available data, we were unable to determine whether engaging with behavioral weight management interventions produces greater or lesser benefits for eating disorder risk than not engaging with obesity services. Additionally, it is important to note that the included trials represent a single weight management attempt, whereas in real world settings people with obesity are likely to engage in multiple weight loss attempts (supported and unsupported, with varying intensity) over time. Thus, the interactions between weight management and eating disorder risk over time are likely to be bidirectional and dynamic. Future research should explore how multiple weight loss attempts influence eating disorder risk, including consideration of support provided and the type and intensity of the intervention. Investigations should also consider whether the time after supervised interventions have ended represents a significant risk for eating disorder emergence.

Most included trials measured binge eating severity or episodes; fewer trials considered global eating disorder risk or compensatory behaviors as symptoms of bulimia nervosa. This is problematic as the focus on binge eating may result in missed symptoms of restrictive eating disorders, for example, atypical anorexia nervosa or other disordered eating behaviors such as laxative misuse. It is possible that symptoms of binge eating are more likely to be measured because of the known associations between dietary restriction and the onset of binge eating,^8^, ^9^ or because binge eating is more common among adults with overweight or obesity compared to those with lower weight.^4^ Nevertheless, case reports in adolescents have shown that recognition and diagnosis of atypical anorexia nervosa can be delayed during weight loss attempts as complications may occur at a higher body weight.^77^ Future trials of behavioral weight management should monitor for the emergence or disordered eating behaviors across the full spectrum of eating disorders.

Current assessment of risk for atypical anorexia nervosa and bulimia nervosa in individuals with overweight and obesity may be limited as assessment tools designed to detect restrictive eating disorders have been developed in lower weight populations and may not have adequate sensitivity when used for individuals with overweight or obesity.^78^ Additionally, there is a lack of guidance for clinicians as to how best to assess and monitor eating disorder risk in people with overweight and obesity. In the context of weight management, eating disorders may develop within a short timeframe (e.g., rapid and significant weight loss during the intervention leading to atypical anorexia nervosa) or may develop slowly over time (e.g., weight regain may trigger repeated attempts at self‐directed weight loss resulting in a restrict‐binge cycle as a pre‐cursor to binge eating disorder). Future research should identify how trials can measure and consider risk for the broad spectrum of eating disorders in adults with overweight or obesity.

For most included trials, data were not available to examine individual changes in eating disorder symptoms. In trials that reported the number of participants engaging in binge eating or compensatory behaviors, a consistent reduction was seen in the number of participants with severe, moderate, or any binge eating. However, four studies reported the onset of binge eating, compensatory behaviors, or binge eating disorder between baseline and follow‐up in a small subset of participants (0.0% to 6.5%). It is not clear if these were new symptoms or the re‐emergence of previous symptoms that were not present at baseline. Only one included trial with a waitlist control reported on the number of participants binge eating and found this reduced in the intervention group with no change in the control group.^14^ It is possible that some trials will have excluded participants with a history of or current eating disorders. Thus, the sample represented within this review may have lower risk than those presenting to obesity treatment services in the community. Nevertheless, this highlights the possibility of the emergence or re‐emergence of disordered eating during weight management interventions for a small subset of participants, the monitoring for which is not routinely incorporated into clinical practice guidelines or care. Considering that eating disorders are likely to affect only a small proportion of participants, analysis of individual participant data is important to identify specific subgroups that may be vulnerable, including whether certain risk factors predict the development of eating disorders in weight management interventions. With the potential for lifelong complications associated with eating disorders and disordered eating,^5^ it is important for mechanisms to be in place during weight management interventions to identify individuals who may be at risk, including screening for a history of eating disorders.

### 4.1 Strengths and limitations

This review included a comprehensive search of published literature and clinical trial registries and is the first review examining eating disorder risk in adults undergoing weight management interventions to include meta‐analyses. We combined both narrative synthesis and meta‐analyses across a broad range of eating disorder outcomes to ensure a comprehensive assessment of weight management interventions. Consideration of eating disorder risk as a safety outcome allowed us to examine risk across a range of interventions. This contributed to a higher heterogeneity between intervention arms; however, prediction intervals indicated that results were robust. Meta‐analysis of aggregate data, conducted as part of this review, represents the likely change in eating disorder risk for most adults. Considering that eating disorders are likely to affect a small proportion of adults undergoing weight management, this review was limited in its ability to report on change within smaller subgroups as sufficient data were not available from individual trials to permit examining individual changes. To understand if the onset of or increase in disordered eating or eating disorders is a possible unintended consequence of weight management for a sub‐group of adults, an individual participant data meta‐analysis is required. The review findings were limited as few trials included measures of global eating disorder risk to assess the full spectrum of eating disorders. Furthermore, all included trials were conducted in developed countries, limiting generalizability of the findings to developing nations.

## 5 CONCLUSION

For most adults, behavioral weight management interventions do not appear to increase eating disorder risk or binge eating. Indeed, a reduction in global eating disorder and binge eating scores is seen following interventions of 4 weeks to 18 months duration, and at follow‐up of up to 36 months from post‐intervention. Although most participants experience less binge‐eating or compensatory behaviors, a small proportion of participants may experience the emergence or re‐emergence of symptoms that could have serious problematic consequences. Future research should seek to identify which participants are most likely to experience this increase in eating disorder risk, that is, whether there are certain risk factors that predict this and the most effective assessment methods for clinical practice. In practice, the relation between behavioral weight management interventions and eating disorder risk is likely to be dynamic. Hence, monitoring for the emergence of disordered eating behaviors in at risk individuals may help ensure the safety of these interventions.

## CONFLICT OF INTEREST STATEMENT

ALA is the Principal Investigator on two publicly funded trials where the intervention is provided by WW (formerly Weight Watchers) at no cost and is a member of the WW Scientific Advisory Board. AJH reports receiving payment for advice given to Slimming World (UK). LAB reports receiving honoraria (paid to a hospital cost centre) for speaking in forums organized by Novo Nordisk in relation to management of adolescent obesity and ACTION‐Teens study. This study is sponsored by Novo Nordisk. It is a multi‐country online study of attitudes toward perceptions of obesity held by adolescents living with obesity, their parents, and health‐care professionals. LAB is the Australian lead of the study.

## Supporting information


**Table S1:** Search strategies
**Table S2:** Table of study characteristics
**Table S3:** Summary of outcome data for included studies reporting mean scores/mean change in score
**Table S4:** Outcome data for studies reporting prevalence or frequency data for a behavior e.g. for binge eating/eating disorder diagnosis
**Figure S1:** Forest plot of the difference in binge eating between the control group and intervention at post. Each estimate was standardized using Hedges' *g*. Mason et al, 2019 had three intervention groups which were combined and compared against the control. A random effects model was used to combine estimates from each trial.
**Figure S2:** Eating disorder risk [Baseline ‐ Post] meta regression. The predicted change in eating disorder risk between baseline and post (Hedge's g) as a function of intervention duration (weeks) using a mixed effects meta‐regression. The grey area captures the bounds of the corresponding 95% confidence interval. Each study estimate is captured in a bubble with a size proportional to its study weight (test of moderators, QM (df = 2) = 0.0087; moderator (duration) beta: −0.00019; Qm p value: 0.92584).
**Figure S3:** Eating disorder risk [Post ‐ Follow‐up] meta regression. The predicted change in eating disorder risk between post and follow‐up (Hedge's g) as a function of follow‐up duration (weeks) using a mixed effects meta‐regression. The grey area captures the bounds of the corresponding 95% confidence interval. Each study estimate is captured in a bubble with a size proportional to its study weight (test of moderators, QM (df = 2) = 0.3446; Moderator time beta: −0.00169; Qm p value: 0.55719).
**Figure S4:** Eating disorder risk [Baseline ‐ Follow‐up] meta regression. The predicted change in eating disorder risk between baseline and follow‐up (Hedge's g) as a function of duration (weeks) using a mixed effects meta‐regression. The grey area captures the bounds of the corresponding 95% confidence interval. Each study estimate is captured in a bubble with a size proportional to its study weight (test of moderators, QM (df = 2) = 0.0735; Moderator time beta: 0.00074; Qm p value: 0.78635).
**Figure S5:** Eating disorder risk [Baseline ‐ Post] assuming a correlation of 0.3. Forest plot of the change in eating disorder risk from baseline to post for each trial. Each estimate was standardized using Hedges g. A correlation of 0.3 was assumed between time points when necessary for the calculation of Hedges' g. A random effects model was used to combine estimates from each trial (prediction lower bound: −0.42, Prediction upper bound: −0.14, Tau^2: 0.0033).
**Figure S6:** Eating disorder risk [Baseline ‐ Post] assuming a correlation of 0.5. Forest plot of the change in eating disorder risk from baseline to post for each trial. Each estimate was standardized using Hedges g. A correlation of 0.5 was assumed between time points when necessary for the calculation of Hedges' g. A random effects model was used to combine estimates from each trial (prediction lower bound: −0.54, Prediction upper bound: 0, Tau^2: 0.0167).
**Figure S7:** Eating disorder risk [Baseline ‐ Post] assuming a correlation of 0.9. Forest plot of the change in eating disorder risk from baseline‐post for each trial. Each estimate was standardized using Hedges g. A correlation of 0.9 was assumed between time points when necessary for the calculation of Hedges' g. A random effects model was used to combine estimates from each trial (Prediction lower bound: −0.67, Prediction upper bound: 0.16, Tau^2: 0.0424).
**Figure S8:** Eating disorder risk [Post ‐ Follow‐up] funnel plot. Funnel plot with the standardized change (Hedges' g) in eating disorder risk between post and follow‐up on the x axis and standard error on the y axis.
**Figure S9:** Eating disorder risk [Baseline ‐ Follow‐up] funnel plot. Funnel plot with the standardized change (Hedges' g) in eating disorder risk between baseline and follow‐up on the x axis and standard error on the y axis.
**Figure S10:** Eating disorder risk [Baseline ‐ Post] funnel plot. Funnel plot with the standardized change (Hedges' g) in eating disorder risk between baseline and post on the x axis and standard error on the y axis.
**Figure S11:** EDE‐Q scale only [Baseline ‐ Post] forest plot. Forest plot that only includes measurements of eating disorder risk that have been assessed with the EDE‐Q tool. Raw scores were used to calculate a mean difference between baseline and post for each trial and a random effects model was used to combine estimates from each trial.
**Figure S12:** EDE‐Q scale only [Post ‐ Follow‐up] forest plot. Forest plot that only includes measurements of eating disorder risk that have been assessed with the EDE‐Q tool. Raw scores were used to calculate a mean difference between post and follow‐up for each trial and a random effects model was used to combine estimates from each trial.
**Figure S13:** EDE‐Q scale only [Baseline ‐ Follow‐up] forest plot. Forest plot that only includes measurements of eating disorder risk that have been assessed with the EDE‐Q tool. Raw scores were used to calculate a mean difference between baseline and follow‐up for each trial and a random effects model was used to combine estimates from each trial.
**Figure S14:** Binge eating [Baseline ‐ Post] meta regression. The predicted change in binge eating between baseline and post (Hedge's g) as a function of intervention duration (weeks) using a mixed effects meta‐regression. The grey area captures the bounds of the corresponding 95% confidence interval. Each study estimate is captured in a bubble with a size proportional to its study weight (test of moderators, QM (df = 2) = 0.0612; Moderator time beta: −0.00064; Qm pvalue: 0.80461).
**Figure S15:** Binge eating [Baseline ‐ Follow‐up] meta regression. The predicted change in binge eating between baseline and follow‐up (Hedge's g) as a function of duration (weeks) using a mixed effects meta‐regression. The grey area captures the bounds of the corresponding 95% confidence interval. Each study estimate is captured in a bubble with a size proportional to its study weight (test of moderators, QM (df = 2) = 0.2075; Moderator time beta: −0.00175; Qm pvalue: 0.64872).
**Figure S16:** Binge eating [Post ‐ Follow‐up] meta regression. The predicted change in binge eating between baseline and post (Hedge's g) as a function of follow‐up duration (weeks) using a mixed effects meta‐regression. The grey area captures the bounds of the corresponding 95% confidence interval. Each study estimate is captured in a bubble with a size proportional to its study weight (test of moderators, QM (df = 2) = 7.1583; Moderator time beta: −0.01122; Qm pvalue: 0.00746).
**Figure S17:** Binge eating [Baseline ‐ Post] assuming a correlation of 0.3. Forest plot of the change in binge eating from baseline‐post for each trial. Each estimate was standardized using Hedges g. A correlation of 0.3 was assumed between time points when necessary for the calculation of Hedges' g. A random effects model was used to combine estimates from each trial (prediction lower bound: −1.26, Prediction upper bound: −0.09, Tau^2: 0.0855).
**Figure S18:** Binge eating [Baseline ‐ Post] assuming a correlation of 0.5. Forest plot of the change in binge eating from baseline‐post for each trial. Each estimate was standardized using Hedges g. A correlation of 0.5 was assumed between time points when necessary for the calculation of Hedges' g. A random effects model was used to combine estimates from each trial (Prediction lower bound: −1.26, Prediction upper bound: −0.05, Tau^2: 0.0929).
**Figure S19:** Binge eating [Baseline ‐ Post] assuming a correlation of 0.9. Forest plot of the change in binge eating from baseline‐post for each trial. Each estimate was standardized using Hedges g. A correlation of 0.9 was assumed between time points when necessary for the calculation of Hedges' g. A random effects model was used to combine estimates from each trial (Prediction lower bound: −1.31, Prediction upper bound: 0.15, Tau^2: 0.1344).
**Figure S20:** Forest plot of the change in binge eating from baseline to post for each trial split into the subgroups minimal or full intervention. Each estimate was standardized using Hedges' g. A correlation of 0.7 was assumed between time points when necessary for the calculation of Hedges' g. A random effects model was used to combine estimates from each trial.
**Figure S21:** Binge eating [Baseline ‐ Post] funnel plot. Funnel plot with the standardized change (Hedges' g) in binge eating between baseline and post on the x axis and standard error on the y axis.
**Figure S22:** Binge eating [Post ‐ Follow‐up] funnel plot. Funnel plot with the standardized change (Hedges' g) in binge eating between post and follow‐up on the x axis and standard error on the y axis.
**Figure S23:** Binge eating [Baseline ‐ Follow‐up] funnel plot. Funnel plot with the standardized change (Hedges' g) in binge eating between baseline and follow‐up on the x axis and standard error on the y axis.
**Figure S24:** BES scale only [Baseline ‐ Post] forest plot. Forest plot that only includes measurements of binge eating that have been assessed with the BES tool. Raw scores were used to calculate a mean difference between baseline and post for each trial and a random effects model was used to combine estimates from each trial.
**Figure S25:** BES scale only [Post ‐ Follow‐up] forest plot. Forest plot that only includes measurements of binge eating that have been assessed with the BES tool. Raw scores were used to calculate a mean difference between post and follow‐up for each trial and a random effects model was used to combine estimates from each trial.
**Figure S26:** BES scale only [Baseline ‐ Follow‐up] forest plot. Forest plot that only includes measurements of binge eating that have been assessed with the BES tool. Raw scores were used to calculate a mean difference between baseline and follow‐up for each trial and a random effects model was used to combine estimates from each trial.
