# Supplementary material for: Eating disorder risk during behavioral weight management in adults with overweight or obesity: A systematic review with meta‐analysis
Source: Obes Rev. 2023 Mar 15;24(6):e13561. doi: 10.1111/obr.13561 (PMC10909435; doi:10.1111/obr.13561)
Supplement: Supplementary file 1 — Table S1: Search strategies Table S2: Table of study characteristics Table S3: Summary of outcome data for included studies reporting mean scores/mean change in score Table S4: Outcome data for studies reporting prevalence or frequency data for a behavior e.g. for binge eating/eating disorder diagnosis Figure S1: Forest plot of the difference in binge eating between the control group and intervention at post. Each estimate was standardized using Hedges' g. Mason et al, 2019 had three intervention groups which were combined and compared against the control. A random effects model was used to combine estimates from each trial. Figure S2: Eating disorder risk [Baseline ‐ Post] meta regression. The predicted change in eating disorder risk between baseline and post (Hedge's g) as a function of intervention duration (weeks) using a mixed effects meta‐regression. The grey area captures the bounds of the corresponding 95% confidence interval. Each study estimate is captured in a bubble with a size proportional to its study weight (test of moderators, QM (df = 2) = 0.0087; moderator (duration) beta: −0.00019; Qm p value: 0.92584). Figure S3: Eating disorder risk [Post ‐ Follow‐up] meta regression. The predicted change in eating disorder risk between post and follow‐up (Hedge's g) as a function of follow‐up duration (weeks) using a mixed effects meta‐regression. The grey area captures the bounds of the corresponding 95% confidence interval. Each study estimate is captured in a bubble with a size proportional to its study weight (test of moderators, QM (df = 2) = 0.3446; Moderator time beta: −0.00169; Qm p value: 0.55719). Figure S4: Eating disorder risk [Baseline ‐ Follow‐up] meta regression. The predicted change in eating disorder risk between baseline and follow‐up (Hedge's g) as a function of duration (weeks) using a mixed effects meta‐regression. The grey area captures the bounds of the corresponding 95% confidence interval. Each study estimate is captured in a bubble wit [file OBR-24-e13561-s001.pdf]

Title: Eating disorder risk during behavioral weight management in adults with overweight or obesity: A systematic review with meta-analysis

Author information:
Hiba Jebeile[1], Sol Libesman[2], Hannah Melville[1], Timothy Low-Wah[1], Genevieve Dammery[3], Anna L. Seidler[2], Rebecca Jones[4], Caitlin M. McMaster[1], Susan J. Paxton[5], Andrew J. Hill[6], Amy L. Ahern[4], Sarah P. Garnett[1,7], Caroline Braet[8], Denise Wilfley[9], Louise A. Baur[1], Natalie B. Lister[1]

[1]Children's Hospital Westmead Clinical School, The University of Sydney, Australia
[2]NHMRC Clinical Trials Centre, The University of Sydney, Australia
[3]InsideOut Institute for Eating Disorders, The University of Sydney, Australia
[4]MRC Epidemiology Unit, University of Cambridge, Cambridge, CB2 0QQ UK [4]School of [5]Psychology and Public Health, La Trobe University, Melbourne, Victoria, Australia
[6]Leeds Institute of Health Sciences, University of Leeds, United Kingdom
[7]Kids Research, The Children's Hospital at Westmead, Australia
[8]Department of Developmental, Personality and Social Psychology, Ghent University, Henri Dunantlaan 2, 9000 Ghent, Belgium
[9]Washington University in St. Louis., United States

Corresponding author: Dr Hiba Jebeile, The University of Sydney, Children's Hospital Westmead Clinical School, Westmead, Australia
Email: hiba.jebeile@sydney.edu.au

Table S1: Search strategies

MEDLINE
1. exp Obesity/
2. exp Overweight/
3. obes*.tw.
4. overweight.tw.
5. 1 or 2 or 3 or 4
6. weight loss/
7. exp diet therapy/
8. exp bariatrics/
9. exp exercise/
10. anti-obesity agents/ or appetite depressants/
11. (diet* adj2 therap*).tw.
12. bariatric*.tw.
13. (low adj3 (energy or calor*) adj4 diet).tw.
14. ((pharma* or diet* or obes* or lifestyle or behavio*) adj3 (interven* or treat* or therap*)).tw.
15. ((calori* or diet*) adj3 (reduc* or restrict*)).tw.
16. (weight adj3 (manag* or los*)).tw.
17. (exercis* or physical activit*).tw.
18. HAES.mp.
19. health at every size.mp.
20. (weight adj2 neutral).mp.
21. nondiet.mp.
22. (non adj2 diet).mp.
23. (intuitive adj2 eat*).mp.
24. mindful*.tw.
25. 6 or 7 or 8 or 9 or 10 or 11 or 12 or 13 or 14 or 15 or 16 or 17 or 18 or 19 or 20 or 21 or 22 or 23 or 24
26. Body Image/
27. (body adj3 (accept* or dissatisf* or image or satisf* or appreciat* or esteem)).tw.
28. "feeding and eating disorders"/ or anorexia nervosa/ or binge-eating disorder/ or bulimia nervosa/ or "feeding and eating disorders of childhood"/
29. (bulimi* adj3 symptom*).tw.
30. (disorder* adj3 eat*).tw.
31. (emotion* adj3 eat*).tw.
32. (diet* adj3 restr*).tw.
33. (binge adj3 eat*).tw.
34. extreme weight loss.tw.
35. loss of control.tw.
36. drive for thinness.tw.
37. ((weight or shape or eat*) adj3 concern).tw.
38. (eat* adj2 behavi*).tw.
39. 26 or 27 or 28 or 29 or 30 or 31 or 32 or 33 or 34 or 35 or 36 or 37 or 38
40. randomized controlled trial/
41. (randomi?ed controlled trial* or RCT* or (controlled adj3 trial)).mp.
42. randomi?ed.ti.
43. clinical trials as topic.sh.
44. randomly.ab.
45. trial.mp.
46. clinical trial.mp.
47. 40 or 41 or 42 or 43 or 44 or 45 or 46
48. 5 and 25 and 39 and 47

EMBASE

1. obesity/
2. obes*.tw.
3. overweight.tw.
4. 1 or 2 or 3
5. weight reduction/
6. diet therapy/ or diet restriction/ or low calory diet/ or low fat diet/
7. bariatric surgery/ or gastric banding/ or sleeve gastrectomy/
8. exercise/
9. antiobesity agent/
10. (diet* adj2 therap*).tw.
11. bariatric*.tw.
12. (low adj4 (energy or calor*) adj4 diet).tw.
13. ((pharma* or diet* or obes* or lifestyle or behavio*) adj3 (interven* or treat* or therap*)).tw.
14. ((calori* or diet*) adj3 (reduc* or restrict*)).tw.
15. (weight adj3 (manag* or los*)).tw.
16. (exercis* or physical activit*).tw.
17. HAES.mp.
18. health at every size.mp.
19. (weight adj2 neutral).mp.
20. nondiet.mp.
21. (non adj2 diet).mp.
22. (intuitive adj2 eat*).mp.
23. mindful*.tw.
24. 5 or 6 or 7 or 8 or 9 or 10 or 11 or 12 or 13 or 14 or 15 or 16 or 17 or 18 or 19 or 20 or 21 or 22 or 23
25. body image/
26. (body adj3 (accept* or dissatisf* or image or satisf* or appreciat* or esteem)).tw.
27. eating disorder/ or anorexia nervosa/ or binge eating disorder/ or bulimia/
28. feeding behavior/
29. (bulimi* adj3 symptom*).tw.
30. (disorder* adj3 eat*).tw.
31. (emotion* adj3 eat*).tw.
32. (diet* adj4 restrain*).tw.
33. (binge adj3 eat*).tw.
34. extreme weight loss.tw.
35. loss of control.tw.
36. drive for thinness.tw.
37. ((weight or shape or eat*) adj3 concern).tw.
38. 25 or 26 or 27 or 28 or 29 or 30 or 31 or 32 or 33 or 34 or 35 or 36 or 37
39. randomized controlled trial/ or controlled clinical trial/
40. (randomi?ed controlled trial* or RCT* or (controlled adj3 trial)).mp.
41. randomi?ed.ti.
42. randomly.ab.
43. trial.mp.
44. clinical trial.mp.
45. 39 or 40 or 41 or 42 or 43 or 44
46. 4 and 24 and 38 and 45

PsycINFO

1. Obesity/
2. Overweight/
3. obes*.tw.
4. overweight.tw.
5. 1 or 2 or 3 or 4
6. weight loss/ or weight control/
7. diets/
8. exp bariatric surgery/
9. exp exercise/
10. (diet* adj2 therap*).tw.
11. bariatric*.tw.
12. (low adj3 (energy or calor*) adj4 diet).tw.
13. ((pharma* or diet* or obes* or lifestyle or behavio*) adj3 (interven* or treat* or therap*)).tw.
14. ((calori* or diet*) adj3 (reduc* or restrict*)).tw.
15. (weight adj3 (manag* or los*)).tw.
16. exercis*.mp. or physical activit*.tw.
17. HAES.mp.
18. health at every size.mp.
19. (weight adj2 neutral).mp.
20. nondiet.mp.
21. (non adj2 diet).mp.
22. (intuitive adj2 eat*).mp.
23. mindful*.tw.
24. 6 or 7 or 8 or 9 or 10 or 11 or 12 or 13 or 14 or 15 or 16 or 17 or 18 or 19 or 20 or 21 or 22 or 23
25. Body Image/
26. (body adj3 (accept* or dissatisf* or image or satisf* or appreciat* or esteem)).tw.

27. eating disorders/ or anorexia nervosa/ or binge eating disorder/ or bulimia/ or hyperphagia/ or "purging (eating disorders)"/<br>28. eating behavior/ or binge eating/ or dietary restraint/<br>29. (bulimi* adj3 symptom*).tw.<br>30. (disorder* adj3 eat*).tw.<br>31. (emotion* adj3 eat*).tw.<br>32. (diet* adj3 restr*).tw.<br>33. (binge adj3 eat*).tw.<br>34. extreme weight loss.tw.<br>35. loss of control.tw.<br>36. drive for thinness.tw.<br>37. ((weight or shape or eat*) adj3 concern).tw.<br>38. 25 or 26 or 27 or 28 or 29 or 30 or 31 or 32 or 33 or 34 or 35 or 36 or 37<br>39. randomized controlled trials/ or clinical trials/ or randomized clinical trials/<br>40. (randomi?ed controlled trial* or RCT* or (controlled adj3 trial)).mp.<br>41. randomi?ed.ti.<br>42. randomly.ab.<br>43. trial.mp.<br>44. clinical trial.mp.<br>45. 39 or 40 or 41 or 42 or 43 or 44<br>46. 5 and 24 and 38 and 45
**SCOPUS**<br>(TITLE-ABS-KEY ("clinical trials" OR "clinical trials as a topic" OR "randomized controlled trial" OR "Randomized Controlled Trials as Topic" OR "controlled clinical trial" OR "Controlled Clinical Trials as Topic" OR "Clinical trial*" OR trial* OR rct OR random*)) AND ((TITLE-ABS-KEY (obes* OR overweight*)) AND ((TITLE-ABS-KEY ("Weight loss" OR diet* OR bariatric* OR exercis* OR "anti-obesity agent*" OR haes OR "health at every size" OR "Weight neutral" OR "Intuitive eat*" OR mindful*)) OR (TITLE-ABS-KEY (((pharma* OR diet* OR obes* OR lifestyle OR behavio*) W/4 (interven* OR treat* OR therap*))))) AND ((TITLE-ABS-KEY (((weight OR shape OR eat*) W/3 concern*))) OR ((TITLE-ABS-KEY ("Body image*" OR "Eating disorder*" OR anorexia OR "binge eating disorder*" OR bulimi* OR "Emotion* eat*" OR "Diet* restr*" OR "Binge eat*" OR "extreme weight loss*" OR "loss of Control" OR "Drive for thinness")) OR (TITLE-ABS-KEY (((weight OR shape OR eat*) W/3 concern*))))))
**Clinicaltrials.gov**<br>Key search terms via basic search platform:<br>(weight management OR obesity treatment)
**WHO ICTRP**<br>Key search terms via basic search platform:<br>(weight management OR obesity treatment)

Table S2: Table of study characteristics

Author, year; Study name (if applicable); Country; Setting (outpatient/ inpatient/ community etc)<br><br>Eating Disorder tool/s<br><br>Quality rating (positive, neutral, negative)[1]	Participant characteristics: Sample Size (n), %female (F), Age, mean (SD); BMI at baseline, mean (SD); Ethnicity; Retention (R; post, FU; %)	Intervention duration<br><br>Duration of follow-up (FU) (from post-intervention)<br><br>Intensity (group, individual, frequency of contact)	Intervention Group (IG), weight-neutral intervention group (WN-IG), control group (CG) – nutrition component; physical activity (PA) component; behaviour change<br><br>Personnel delivering intervention (P)	BMI (weight if BMI NR) outcome (all timepoints, mean change (SD/SE))
Afari et al. 2019;[2] MOVE! Programme; USA; Community<br><br>BES<br><br>Positive	n=88, 23.9% F<br><br>57.3 (9.9)y; 7.2 (7.0)kg/m^2; African-American 19.3%, Caucasian 70.5%, Hispanic 13.6%<br><br>R: IG1 – 12-wk = 100%, 24-wk = 100%; IG2 – 12-wk = 100%, 24-wk = 98%	4-wk<br><br>12-wk & 24-wk FU<br><br>IG1 and IG2 4x 2 hours weekly group session	**IG1:** Acceptance and Commitment therapy group (ACT) – Participants taught to notice thoughts, emotions and urges related to eating and to allow their values to drive behavior rather than avoidance of negative internal experiences. The intervention stressed the importance of at-home assignments to develop skills taught in session.<br><br>**IG2:** Behavioral weight loss group (BWL) - program incorporated cognitive–behavioral techniques to target distorted thinking related to food consumption and physical activity, as well as strategies to maintain treatment gains (e.g. goal setting, focusing on strengths and being optimistic). Participants completed food and exercise logs<br><br>**P**: Psychologist, psychology post doc x2, psychology masters student x1 (IG1); Psychologist, psychology post doc, psychology master's student (IG2)	BMI NR<br><br>Weight (lbs):<br>IG1: B = 250.7 (63.0), Post = 249.6 (64.2), 12-wk FU= 246.2 (62.7), 24-wk FU= 248.5 (63.2)<br><br>IG2: B = 249.9 (53.1), Post = 248.4 (55.7), 12-wk FU = 243.8 (51.6), 24-wk FU = 246.5 (50.3)<br><br>Mean difference between groups (lbs, 95% CI): Post = 0.49 (-16.9, 17.9), 12-wk FU = -1.41 (-11.3, 8.49), 24-wk FU = -1.13 (-13.2, 10.9)
Ariel et al. 2016; [3] USA; Community<br><br>BES<br><br>Neutral	n=612, 78.3% F<br><br>IG1 51.5 (12.3)y, 36.1 (4.2)kg/m^2; IG2 52.8 (10.6)y, 36.2 (3.8)kg/m^2; IG3 53.2 (12.0)y, 36.7 (4.0)kg/m^2; IG4 52.0 (10.8)y, 36.3 (3.9)kg/m^2<br><br>Black, Non-Hispanic 15.5%; Hispanic 3.7%; White, Non-Hispanic 77.7%; Other/multiple 2.9%<br><br>Retention NR	24-mo<br><br>Nil FU<br><br>Phase 1, initial weight-loss induction, and Phase 2, extended care.<br>Phase 1 consisted of weekly sessions (8 for LOW, 16 for MOD, and 24 for HIGH).<br>Phase 2 targeted maintenance of behavior change and was conducted on a faded schedule, using a combination of scheduled telephone sessions and office-based "campaign sessions."	The contents of the lifestyle program employed in the LOW, MOD, and HIGH conditions included:<br>(a) a low-calorie eating pattern (1,200 kcal/day for participants weighing <114 kg, 1,500 kcal/day for those weighing 114-136 kg, and1,800 kcal/day for those weighing>136 kg);<br>(b) increased physical activity in the form of 30 min/day of walking above baseline levels; and<br>(c) training in behavior modification strategies including goalsetting, self-monitoring, stimulus control, cognitive restructuring, and problem solving.<br><br>**IG1 – Low Dose:** 16 sessions of behavioral lifestyle treatment over two years<br><br>**IG2 – Moderate Dose:** 32 sessions of behavioral lifestyle treatment over two years	% reductions in initial body weight (mean, 95% CI)<br>IG1: 6-mo = 7.2 (6.1,8.3), 24-mo = 3.5 (2.0,4.8)<br><br>IG2: 6-mo = 9.3 (8.2,10.3), 24-mo = 6.7 (5.3,7.9)<br><br>IG3: 6-mo = 10.9 (9.8,11.9), 24-mo = 6.8 (5.5,8.1)<br><br>IG4: 6-mo = 4.1 (3.1,5.1), 24-mo = 2.9 (1.7,4.3)

			**IG3 – High Dose:** 48 sessions of behavioral lifestyle treatment over two years<br><br>**IG4 – Education:** This acted as a control for staff attention and for the delivery of appropriate information regarding proper diet and exercise for weight management. The schedule of sessions provided to participants in the CONTROL condition was identical to that of the LOW dose lifestyle condition.<br><br>**P:** The interventionists for all conditions were Cooperative Extension Service Family and Consumer Sciences Agents or individuals with bachelors or master's degrees in nutrition, exercise science, or psychology.	
Bacon et al. 2002; Bacon et al. 2005;[4,5] USA; Community<br><br>EDI<br><br>Positive	n=78, 100% F<br><br>30-45y, 39.3 (4.5)y; 35.7 (3.6)kg/m^2<br><br>Ethnicity NR<br><br>R=59%	24-wk<br><br>52-wk FU<br><br>24x weekly group sessions of 90 mins with optional 6 month after-care program as monthly group support sessions and no new materials presented	**IG-WN:** Non-diet treatment program – educational and psychotherapeutic workshops using the non-diet approach in five aspects: body acceptance, eating behavior, activity, nutrition, and social support.<br><br>**IG:** Standard behavioral weight loss program –using the LEARN Program for Weight Control manual which focuses on eating behaviors and attitudes, nutrition, social support, and exercise. Participants were encouraged to moderately reduce their fat and energy intake, maintain a food diary, and monitor weight weekly.<br><br>**P:** IG-WN was facilitated by a counsellor experienced non-diet approach. IG was delivered by registered dietitian.	BMI (kg/m^2)<br>IG-WN: (n=29) B = 36.6 (4.1), 12-wk = 35.2 (4.2), Post = 34.9 (4.2), 52-wk FU = 34.5 (3.5)<br><br>IG: (n=23) B= 35.9 (4.1), 12-wk = 36.1 (4.1)†, Post = 36.1 (3.9)†, 52-wk = 36.1 (4.1)†*<br><br>*a significant between-group difference<br>†a significant within-group difference from baseline<br><br>Mean change: significant reduction in mean BMI from 36.6 to 34.5 in IG post-aftercare (52 weeks), no significant change in BMI in IG<br><br>Mean BMI (kg/m^2) within group change data NR
Barnes et al. 2014; Barnes et al. 2017;[6,7] USA; Community<br><br>EDE-Q<br><br>Positive	n=59, 74.6% F<br><br>22-65y, 48.0 (10.7)y, White 66.1%<br><br>IG1 47.07 (9.97)y, 34.65 (7.06)kg/m^2; IG2 48.93 (11.59)y, 35.07 (7.52)kg/m^2; IG3 47.77 (10.05)y, 36.08 (6.44)kg/m^2	3-mo<br><br>3-mo & 12-mo FU<br><br>IG1: 5 sessions over 12 weeks, initial 60-mins in-person individual session, 4x 20-mins motivational interviewing sessions	**IG1:** Motivational interviewing and internet condition (MIC) – delivered by medical assistants using motivational interviewing strategies to motivate patients for behavioral changes to support weight loss. Participants were given a Lifestyle, Exercise, Attitudes, Relationships, and Nutrition (LEARN) manual and access to a free website for tracking food intake, setting weight and intake goals, and physical activity (Livestrong.com)<br><br>**IG2:** Nutrition psychoeducation and internet condition (NPC) – delivered by medical assistants providing basic	BMI (kg/m^2)<br>IG1: n=30; B=34.65 (7.06)<br>12-mo minus baseline = n=21; 0.474 (2.025)<br><br>IG2: n=29; B=35.07 (7.52)<br>12-mo minus baseline = n=23; -0.521 (1.544)<br><br>Group difference = 0.996 (-0.094, 2.085)

	R for 12 months follow-up: IG1 = 76.7%; IG2 = 89.7%; Overall = 83.1%	IG2: 5 sessions over 12 weeks, initial 60-mins in-person individual session, 4x 20-mins sessions<br><br>IG3: no contact during intervention, offered compassionate care (MIC) after completing 3-month follow-up assessment	nutritional information e.g. recommended daily intake of food groups. Participants were given a Lifestyle, Exercise, Attitudes, Relationships, and Nutrition (LEARN) manual and access to a free website for tracking food intake, setting weight and intake goals, and physical activity (Livestrong.com)<br><br>**IG3:** Usual care – participants were encouraged to continue working with their primary care providers and were not given LEARN manual or guidance for the website. They were offered compassionate care (MIC) after the 3-month follow-up assessment, but this data was not presented<br><br>**P:** Medical Assistants (MAs) delivered IG1 and IG2. MAs did not have prior weight loss treatment or motivational interviewing (MI) training. Four MIC clinicians attended two eight-hour training sessions by a member of the Motivational Interviewing Network of Trainers.	IG3 - NR
Beaulieu et al. 2020;[8] UK; Community<br><br>BES<br><br>Positive	n=46, 100% F<br><br>IG1 34 (9)y, 28.9 (2.3)kg/m^2; IG2 35 (11)y, 29.4 (2.5)kg/m^2.<br><br>Ethnicity NR<br><br>Retention NR	12-wk<br><br>Nil FU<br><br>Both IGs had weekly progress meetings with a dietitian	**IG1:** Continuous energy restriction (CER) – dietitian calculated energy requirements based on RMR x PAL and created meal plan for participants based on the requirements and food preferences. All food were pre-portioned except liquids. The participants consumed 75% of their daily energy requirements each day from commercially available products. The macronutrient composition of the diet was 50–55% carbohydrate, 30–35% fat, and 15–20% protein.<br><br>**IG2:** Intermittent energy restriction (IER) – dietitian calculated energy requirements based on RMR x PAL and created meal plan for participants based on the requirements and food preferences. All food were pre-portioned except liquids. The participant had alternating fast days and food intake ad libitum. On fast days, participants consumed 25% of their daily energy requirements from total diet replacement products (LighterLife Ltd), there were no time restrictions on when food needed to be consumed. The calorie content (~150 kcal) and macronutrient composition (~36% carbohydrate, ~27% fat, and ~37% protein) was similar for each product, and ensured a daily protein intake of 49.2 ± 8.2 g.<br><br>**P:** Participants were given the details of their meal plan (i.e. CER or IER) by the research dietitian	BMI (kg/m^2)<br>IG1: n=22; B = 29.1 (2.4), Post = n=18; 27.3 (2.3)*<br><br>IG2: n=24; B = 29.2 (2.5), Post = n=12; 27.2 (2.4)*<br><br>Mean weight and BMI within group change data NR
Bolognese et al. 2020;[9] Brazil; Community	n=74, 100% F	12-wk	**IG1:** Group nutrition counselling group – weekly group consultation with a registered nutritionist who provided	BMI and weight outcomes NR

EAT<br><br>Positive	40-59y, 45.7 (3.2)y<br><br>BMI NR<br><br>Ethnicity NR<br><br>R=36.4%	Nil FU<br><br>IG1: once a week with registered nutritionist for approximately 40 mins<br><br>IG2: monthly consultations with registered nutritionist for approximately one hour, participants had initial evaluations and fortnightly visits if necessary<br><br>Resistance-training: IG1 and IG2, 3x week physical exercise for an average of 47-62 mins with a physical trainer.	educational materials and strategies focusing on nutrition counselling and eating behavior changes. Participants performed alternating resistance and aerobic exercises with a physical trainer 3 times a week.<br><br>**IG2:** Individualized nutrition prescription group – monthly individual consultation with registered nutritionist where a meal plan is prescribed. Energy requirements calculated based on RMRx1.4PAL, adherence to diet was not monitored daily. Participants performed alternating resistance and aerobic exercises with a physical trainer 3 times a week.<br><br>**P:** The participant were attended by a certified nutritionist.	BMI time effect F = 30.69, $p < 0.001$<br><br>BMI between group difference:<br>IG1 d = -0.24 (medium effect)<br>IG2 d = -0.23 (medium effect)<br><br>Mean weight and BMI within group change data NR
Carels et al. 2014; [10,11] USA; Community<br><br>BES<br><br>Positive	n=59, 78% F<br><br>18-65y, 44.3 (13.2)y, 39.7 (10.3)kg/m^2, Caucasian 86%<br><br>R=73%, 60% FU	12-wk<br><br>6-mo FU<br><br>IG1 & IG2 had weekly 90-minute group sessions	**IG1:** New Perspectives weight loss program – the aim of this program was to facilitate weight loss through the systematic deconstruction of misinformation, encourage exploration of attitudes that contribute to unhealthy lifestyle behaviors, and provide tools to rebuild a healthier and more adaptive attitude toward food, weight, and one's body.<br><br>**IG2:** Transforming Your Life weight loss program – the aim of this program was to facilitate weight loss through emphasis on healthy habit formation and disruption of unhealthy habits and changing our food and exercise environment to minimize unhealthy habits.<br><br>**P:** All assessments and interventions were conducted by a licensed clinical health psychologist or psychology doctoral students with experience in leading weight loss interventions.	BMI NR<br><br>Overall treatment effect for weight loss in both groups from baseline to post-treatment (lbs) = 66.38, $p < 0.001$<br><br>Weight loss between IG1 and IG2 groups (lbs) = 0.03, p = 0.87<br><br>Mean weight (lbs) change data only reported on in a graph

Carels et al. 2019;[12] USA; Community<br><br>BES<br><br>Positive	n=94, 70.2% F<br><br>19-73y, 46.0y, 36.4 (6.5)kg/m^2, European American 58.5%, African American 37.2%, Asian 2.1%, Hispanic 1.1%<br><br>R=56%	16-wk<br><br>Nil FU<br><br>IG1 weekly 90mins weight loss group for 8 weeks. IG2 and IG3, no contact, continued with DPP manual.	All participants were provided a self-help intervention adapted from the Diabetes Prevention Program (DPP), Fitbit Zip and access to MyFitnessPal. The first 8 weeks were self-monitoring of eating behaviors and exercise (10,000 steps and 150 mins of brisk physical activity per week). Weight loss target of 2.5% of body weight. Then participants who did not meet the 2.5% weight loss goal were randomized into groups:<br><br>**IG1:** Acceptance-based weight loss group (MISS-ABT) – participants who did not meet the 2.5% weight loss goal, they attended weekly 90 mins group consultations and were provided an acceptance-based weight loss manual focusing on Acceptance-base therapy principles such as acceptance, willingness, values, defusion, and committed action.<br><br>**IG2:** Self-help weight loss group (MISS-SH) – participants who did not meet the 2.5% weight loss goal, were provided with the same instructions as participants who met the weight loss goal.<br><br>**IG3:** Self-help weight loss group (MET-SH) – participants who met the 2.5% weight loss goal, were provided with the subsequent eight chapters of the DPP weight loss manual.<br><br>**P:** NR	BMI NR<br><br>Total average weight loss of 2.96% (3.88)<br><br>Weight loss (%):<br>IG1: 0-8 week = 0.76 (1.20), 9-16 week = 0.50 (2.21), Mean difference = 0.26 (2.63), $p = 0.707$<br><br>IG2: 0-8 week = 1.10 (1.80)*, 9-16 week = -0.70 (2.44), Mean difference = 1.80 (2.00), $p = 0.007$<br><br>IG3: 0-8 week = 4.50 (1.67)*, 9-16 week = 0.82 (2.42), Mean difference = 3.68 (2.51), $p < 0.001$<br><br>Weight (kg) change data NR

Carpenter et al. 2019;[13] USA; Community<br><br>BES<br><br>Positive	n=75, 92% F<br><br>26-68y, 47.3 (10.0)y,31.5 (2.3)kg/m^2, White 65.3%, Black 26.7%, Hispanic 6.7%, Asian 1.3%<br><br>R=92%	6-mo<br><br>Nil FU<br><br>IG1 & IG2: 11x 20-30 mins proactive phone-based counselling sessions with registered dietitian (2/11) and health coach (9/11). Participants could choose whether these phone sessions were done weekly or biweekly, after the 11 sessions participants were allowed unlimited call access to health coaches.	**IG1:** Mind Your Weight weight loss program – participants were provided with 11 phone coaching sessions with dietitian and health coach, each session started off with a 60 seconds mindfulness exercise. Health coach would check in about progress on weight goal, food tracking and physical activity followed by a discussion of a mindfulness topic. These topics may include: meditation, mindfulness of everyday activities, mindful eating, acceptance of thoughts and emotions and self-compassion. Participants were given mindfulness eLessons and resources.<br><br>**IG2:** Weight Talk weight loss program – based on the National Institute of Health (NIH) clinical guidelines on identification, evaluation, and treatment of overweight and obesity in adults, the diabetes prevention program, and Dietary Approaches to Stop Hypertension (DASH) diet. Participants were provided with 11 phone-based counselling sessions with dietitian and health coach accompanied with an integrated website and Fit-bit Zip activity tracker.<br><br>**P:** Sessions delivered by registered dietitian and health coach	BMI NR<br><br>% weight loss<br>IG1: n=45; B = 0.0, Post = 2.7 (4.9)<br><br>IG2: n=24; B = 0.0, Post = 3.1 (3.7)<br><br>Weight loss (kg)<br>IG1: n=45; B = 0.0, Post = 2.4 (4.4)<br><br>IG2: n=24; B = 0.0, Post = 2.6 (3.2)
Cheng et al. 2014;[14] Australia; Community<br><br>BES<br><br>Neutral	n=71, 100% F<br><br>18-25y<br><br>IG1 22.4 (0.5)y, 34.6 (0.7)kg/m^2, Caucasian 80%, Asian 5%, South American 5%, Mixed 10%; IG2 22.1 (0.5)y, 32.2 (0.9)kg/m^2, Caucasian 86%, Asian 7%, African 7%<br><br>R=IG1 = 57%; IG2 = 43%	12-mo<br><br>Nil FU<br><br>IG1 & IG2: 27x dietetic and behavior modification sessions, weekly from 0-3 months, fortnightly from 3-6 months, monthly from 6-12 months.	**IG1:** Higher-protein diet (HP) – energy restriction diet providing 32% protein, 41% carbohydrates, 25% fat per day. Limited alcohol consumption (<20 g/week) and low glycemic-index foods (GI < 55) were recommended. Total energy intake was 5,600 kJ/day, saturated fat limited to 10% daily energy and total fat intake was 37.9 g. Participants were given a 10-module behavior modification program targeting healthy eating habits and the control and modification of eating behaviors.<br><br>**IG2:** Higher-carbohydrate diet (HC) – energy restriction diet providing 20% protein, 58% carbohydrate, 21% fat per day. Total energy intake was 5,600 kJ/day, saturated fat limited to 10% daily energy and total fat intake was 37.9g. Participants were given a 10-module behavior modification program targeting healthy eating habits and the control and modification of eating behaviors.<br><br>**P:** NR	BMI (kg/m^2)<br>IG1: B = n=36; 34.1 (32.7-35.5) 6-mo completers = n=24; 34.3 (32.8-35.7), 12-mo completers = n=21; 34.6 (33.1-36.2)<br><br>IG2: B= n=35; 33.8 (32.1-25.5), 6-mo completers = n=20; 32.2 (30.6-33.9), 12-mo completers = n=15; 32.2 (30.2-34.2)
Christaki et al. 2013;[15] Greece; Outpatient<br><br>EAT-26	n=34, 100% F	8-wk<br><br>Nil FU	**IG1:** Self-administered stress management program (SM) – low energy balanced diet with energy deficit of 2510 kJ/day (45-50% carbohydrates, 30-35% fat, 15-20% protein) using the Mediterranean diet regime. Stress	Mean BMI (kg/m^2) change post-treatment:<br>IG1 = n=18; -1.63 (0.29)<br>IG2 = n=16; -0.53 (0.28)

Neutral	IG1 44.06 (11.11)y, 39.42 (7.29)kg/m^2; IG2 52.81 (10.13)y, 36.76 (7.07)kg/m^2<br><br>Ethnicity NR<br><br>R=56.7%	IG1: 3x 20 mins individual nutrition consultation + 40 mins stress management<br><br>IG2: 3x 20 mins individual nutrition consultation	management sessions included progressive muscular relaxation and diaphragmatic breathing techniques with CD recorded instructions.<br><br>**IG2:** Diet-control group – low energy balanced diet with energy deficit of 2510 kJ/day (45-50% carbohydrates, 30-35% fat, 15-20% protein) using the Mediterranean diet regime.<br><br>**P:** Consultations led by clinical nutritionist who specialized in stress management.	
Cooper et al. 2010;[16] UK; Community<br><br>EDE<br><br>Incidence and frequency of binge eating<br><br>Positive	n=150, 100% F<br><br>IG1 41.20 (8.77)y, 33.85 (2.71)kg/m^2; IG2 41.38 (9.90)y, 34.79 (3.06)kg/m^2; IG3 = 41.86 (8.67)y, 35.41 (2.71)kg/m^2<br><br>Ethnicity NR<br><br>R=86%	44-wk (IG1 and IG2)<br>24-wk (IG3)<br><br>6, 12, 24 and 36-mo FU<br><br>IG1 and IG2: 24x 50 mins 1-on-1 sessions over 44 weeks, 7x weekly sessions then 17x biweekly<br><br>IG3: 2x initial face-to-face session, followed by 15-20 mins telephone sessions for 24 weeks	**IG1:** Cognitive behavior therapy group – modified CBT treatment to encourage the acquisition and practice of weight maintenance skills to sustain long-term weight loss. For the first 24-30 weeks of treatment, participants were instructed on an energy-deficit of 1500 kcal/day, afterwards for the remainder of the treatment focusing on weight maintenance strategies.<br><br>**IG2:** Behavior therapy group – based on the Pittsburgh behavioral weight control manual to provide individualized treatment to match participant needs and progress. Participants were instructed on an energy-deficit of 1200 kcal/day, between weeks 24 and 30, and again at week 36, participants were given a choice to continue to pursue further weight loss or switch to weight maintenance strategies to maintain new lower weight.<br><br>**IG3:** Guided self-help group – based on the LEARN program which is a commonly used weight control program, participants were instructed on an energy-deficit of 1200 kcal/day and gradually increase physical activity with limited support and guidance from a therapist using a guided self-help mode of treatment delivery.<br><br>**P:** There were three therapists, and each delivered all three treatments after a six-month period of training. All three treatments were fully manualized. Two of the therapists were clinical psychologists and one was a dietician.	BMI NR<br><br>Weight (kg)<br>IG1: B = n=49; 92.34 (8.81), Mid = n=49; 83.20 (10.39), Post = n=49; 84.17 (11.11), 6-mo FU = n=49; 86.76 (11.21), 12-mo FU = n=49; 89.02 (11.48), 24-mo FU = n=49; 91.44 (11.17), 36-mo FU = n=49; 91.86 (10.69)<br><br>IG2: B = n=50; 95.20 (11.15), Mid = n=50; 84.48 (12.64), Post = n=50; 83.60 (14.60), 6-mo FU = n=50; 86.46 (14.38), 12-mo FU = n=50; 88.24 (14.34), 24-mo FU = n=50; 90.86 (12.94), 36-mo FU = n=50; 91.99 (13.43)<br><br>IG3: B = n=51; 95.94 (9.18), Mid = n=51; 89.52 (11.58), Post = n=51; 90.70 (11.66), 6-mo FU = n=51; 92.97 (11.65), 12-mo FU = n=51; 93.64 (11.04), 24-mo FU = n=51; 95.14 (11.61), 36-mo FU = n=51; 95.90 (10.89)<br><br>% Weight loss from baseline<br>Total: Mid = -9.32 (7.13), Post = -9.01 (8.92), 6-mo FU = -6.13 (8.61), 12-mo FU = -4.47 (8.13), 24-mo FU = -2.14 (7.32), 36-mo FU = -1.26 (7.65)

Dalle Garve et al. 2013;[17]<br>Italy; Inpatient/Outpatient<br><br>BES<br><br>Positive	n=88, 58% F<br><br>19-65y, 46.7 (11.1)y<br><br>IG1 46.7 (10.3)y, 45.8 (6.5)kg/m^2; IG2 45.6 (12.0)y, 45.4 (7.0)kg/m^2<br><br>Ethnicity NR<br><br>R=IG1 = 74%, IG2 = 82%	12-mo<br><br>Nil FU<br><br>Stage 1 – inpatient intervention with 15x CBT groups, 18x aerobic exercise sessions, 6 callisthenic sessions.<br>Stage 2 – 12x 45 mins dietetic consultations, first 4 sessions fortnightly, then 4 sessions monthly, and then 4 sessions every 6 weeks	**IG1:** High protein diet (HPD) with CBT weight loss intervention – energy-restricted diet with 1200 kcal/day for women, 1500 kcal/day for men (20% fats (<10% saturated fats), 34% protein, 46% carbohydrates). Diet commenced as inpatient in Stage 1 for 3 weeks, attending 15 CBT groups (5 sessions/week) based on principles of the LEARN program with team of physicians, dietitians, and psychologists, and 18 sessions of aerobic exercise and 6 sessions of callisthenic sessions with a physical trainer. Stage 2 involves 12 outpatient consultations with dietitian focusing on weight maintenance.<br><br>**IG2:** High carbohydrate diet (HCD) with CBT weight loss intervention – energy-restricted diet with 1200 kcal/day for women, 1500 kcal/day for men (20% fats (<10% saturated fats), 17% protein, 63% carbohydrates). Diet commenced as inpatient in Stage 1 for 3 weeks, attending 15 CBT groups (5 sessions/week) based on principles of the LEARN program with team of physicians, dietitians and psychologists, and 18 sessions of aerobic exercise and 6 sessions of callisthenic sessions with a physical trainer. Stage 2 involves 12 outpatient consultations with dietitian focusing on weight maintenance.<br><br>**P:** Stage 1 – Groups were chaired by physicians, dieticians and psychologists. Stage 2 – Sessions were delivered by a CBT-trained dietitian.	BMI (kg/m^2)<br>IG1: n=43; 3-wk = 43.7 (5.9), 27-wk = 39.2 (5.8), 1-yr = 39.6 (6.1)<br><br>IG2: n=45; 3-wk = 43.3 (6.7), 27-wk = 39.2 (6.9), 1-yr = 39.7 (7.0)<br><br>Mean BMI (kg/m^2) change from baseline:<br>IG1: n=43; 3-wk = -2.0 (0.8), 27-wk = -6.5 (3.5), 1-yr= -6.2 (4.5)<br><br>IG2: n=45; 3-wk = -1.9 (0.8), 27-wk= -6.1 (2.7), 1-yr = -5.7 (3.3)
Dassen et al. 2018;[18]<br>Netherlands; Community<br><br>EDE-Q<br><br>Positive	n=91, 74.7% F<br><br>IG1 46.29 (11.89)y, 30.96 (3.64)kg/m^2; IG2 50.10 (8.60)y, 30.49 (3.97)kg/m^2<br><br>Ethnicity NR<br><br>R=73.6%	42 days (average)<br><br>FU 6-mo<br><br>25 training sessions online, daily reminders were sent to participants. Minimum interval of 24 hours and a maximum interval of 48 hours between sessions.	All participants received online psychoeducation about weight loss and a healthy lifestyle, while completing the 25 sessions of working memory (WM) training or sham training at home.<br><br>**IG1**: Online lifestyle intervention with gamified working memory (WM) training (experimental condition). The WM training was developed as a serious game, a game specifically designed to improve cognitive ability by adding game-elements to the original training. All sessions and tasks were presented in an online restaurant-setting. To complete a full session, participants had to practice three WM tasks: a visuospatial WM task, a backward digit span task and an object memory task<br><br>**IG2:** Sham training at home alongside lifestyle intervention<br><br>The lifestyle intervention used general nutrition information and principles of cognitive behavioral therapy.	BMI (kg/m^2)<br>IG1: B = n=51; 30.96 (3.64), Post (6-wk) = n=34; 29.95 (3.46), FU1 = 29.78 (3.56), FU2 (26-wk) = n=33; 29.65 (3.80)<br><br>IG2: B= n=40; 30.49 (3.97), Post (6-wk) = n=36; 30.17 (4.14), FU1 = 30.28 (4.31), FU2 (26-wk) = n=33; 30.34 (4.55)<br><br>On the average, participants lost a total of respectively1.26% of their BMI at post-test, 1.52% at FU1 and 1.61% at FU2 relatively to baseline. The main effect of condition was not significant (p>0.47).

			It combined general principles of weight loss and motivation to lose weight, advice to keep track of daily caloric intake via an online tool, topics such as the 'obesogenic' environment, healthy weight loss and nutrition, designing a personal diet plan. The third lifestyle session addressed several aspects of physical activity, such as the health benefits of regular physical activity and implementing physical activities in daily life. The fourth session discussed strategies for dealing with difficult moments and gave tips to maintain a healthy weight after the intervention.<br><br>**P:** This training was delivered online and developed by Fania Dassen (Cognitive Neuropsychiatry and Clinical Neuroscience)	
Dennis et al. 2001;[19] USA; Community<br><br>BES<br><br>Positive	N=82, 100% F<br><br>50-65y, 59.9 (5.7)y<br><br>IG1 (Assured) 31.9 (4.5)kg/m^2; IG2 (Disbelievers) 34.4 (4.9)kg/m^2<br><br>Ethnicity NR<br><br>R=28%	6-mo<br><br>Nil FU<br><br>24x weekly 1 hour classroom sessions plus 45 mins walking period over 6 months	At baseline, women were stratified by self-efficacy type - Assured or Disbeliever - and the randomly assigned to one of three treatment groups. Assured were assigned to either AT (IG1) or NT (IG3). Disbelievers were randomly assigned to DT (IG2) or NT (IG3)<br><br>**IG1:** Assured treatment (AT) – multi-faceted weight loss treatment including heart-healthy diet (300-500 calories deficit per day, target of 0.5 kg/week weight loss), low-intensity walking (3x 45 mins walking sessions/week), lifestyle behavior change and self-efficacy-based treatment based on the 4 Bandura dynamics: performance accomplishment, vicarious experience, verbal per- suasion, and emotional arousal. The AT aimed to support and further strengthen the confident efficacy beliefs of the assured type of obese women by fostering positive expectations of weight management.<br><br>**IG2:** Disbeliever treatment (DT) – multi-faceted weight loss treatment including heart-healthy diet (300-500 calories deficit per day, target of 0.5 kg/week weight loss), low-intensity walking (3x 45 mins walking sessions/week), lifestyle behavior changes and self-efficacy-based treatment based on the 4 Bandura dynamics: performance accomplishment, vicarious experience, verbal per- suasion, and emotional arousal. The DT aimed to build and instill confidence in disbeliever women for weight control behavior change; thereby converting women from the disbeliever to the assured type. DT was intensely structured and organized, using components of Social Learning Theory.	BMI (kg/m^2)<br>Assureds in AT (IG1): B = 32.6 (4.1), Post = 30.3 (4.9)<br><br>Assureds in NT (IG2): B = 31.1 (5.0), Post =28.1 (4.8)<br><br>Disbelievers in DT (IG3): B = 35.7 (4.2), Post = 32.8 (5.2)<br><br>Disbelievers in NT (IG4): B = 33.5 (5.4), Post = 30.6 (3.5)

			**IG3:** Non-targeted treatment (NT) – participants followed the same diet and walking schedule as AT and DT groups, but the NT program was more focused on nutrition with no targeted self-efficacy content.<br><br>**P:** A dietitian gave education on a heart-healthy diet	
Dennis et al. 1999;[20] USA; Military<br><br>BES<br><br>Positive	n=39, 0% F<br><br>IG1 31.9 (0.1)y, 108.1 (10.2)kg; IG2 30.4 (5.7)y, 106.7 (12.0)kg<br><br>Ethnicity NR<br><br>R=79.5%	16-wk<br><br>Nil FU<br><br>16x 1-hour weekly group lecture and discussion sessions with dietitian	**IG1:** Treatment group – lifestyle behaviors weight control program including diet (heart-healthy guidelines – 50-55% carbohydrate, <20% protein, <30% fat; 500 calories/day deficit for 0.5-1.0 kg weight loss/week), behavior modification, cognitive/emotional/social determinants of weight management, and exercise (4x 1 hr exercise per week).<br><br>**IG2:** Navy's usual treatment – no intervention applied or recommended, nutrition factsheets provided upon request and participants were directed to follow usual Navy's exercise routine (4x 1 hr exercise per week).<br><br>**P:** The program format was small group lecture and discussion conducted by a Navy dietitian.	BMI (kg/m^2)<br>IG1: B = n=21; 33.9 (2.7), Post = n=21; 31.3 (3.3)<br><br>IG2: B = n=18; 33.0 (2.9), Post = n=18; 31.2 (3.0)
DiMarco et al. 2009;[21] USA; Community<br><br>EDE-Q<br><br>Neutral	n=39, 82% F<br><br>20-54y, 39.9 (8.84)y, 32.36 (3.05)kg/m^2, Caucasian 71.8%, African American 7.7%, Hispanic/Latino 5.1%, South Asian 5.1%, East Asian 2.6%, Other 7.7<br><br>R=66%	12-wk<br><br>Nil FU<br><br>Total of 8 treatment session – 2x 1-hour sessions weekly, then 3x 30 mins sessions weekly, then 3x 30 mins sessions bi-weekly	**IG1:** Guided self-help with motivational interviewing (GSH/MI) behavioral weight loss treatment – program based of LEARN manual for behavioral weight loss incorporated with motivational interviewing techniques.<br><br>**IG2:** Guided self-help behavioral weight loss treatment – treatment as usual control, program based of LEARN manual for behavioral weight.<br><br>**P:** Therapists were graduate students in clinical psychology who received training in MI from Thomas Morgan, Psy.D. Dr. Morgan's training was tailored to the study manual.	BMI (kg/m^2)<br><br>IG1: B = n=20; 33.06 (3.17), Post = n=20; 31.58 (3.08)<br><br>IG2: B = n=19; 31.62 (2.81), Post = n=19; 30.92 (3.05)
Fogelholm et al. 1999;[22] Finland; Community<br><br>BITE<br><br>Neutral	n=85, 100% F<br><br>29-46y, 34kg/m^2<br><br>Ethnicity NR<br><br>R=94%	52-wk<br><br>(12-wk weight reduction phase, WR, followed by a 40-wk weight maintenance phase, WM)<br><br>Nil FU<br><br>Weekly small group (5-12 people) sessions for 1 year	All participants undergo a 12-week weight reduction period (WR) on a low-energy diet during weeks 1 and 10-12 and very-low-energy diet (Nutrilett^R, Nycomed Pharma AS, to cover 40% of resting energy requirements) during weeks 2-9. All participants had weekly small group sessions with a nutritionist. After the weight reduction period, the participants underwent a weight maintenance period (WM) following a low-fat diet and randomized into 3 different physical activity groups:<br><br>**IG1:** W1 group – a walking program targeting 1000kcal expenditure (average of 2-3 hrs walking) per week.	BMI NR<br><br>Weight (kg)<br>IG1: Weight before WR = n=81; 90.8 (1.6), Weight change during WR = n=81; -13.0 (0.7), Weight change during WM = n=80; -0.7 (1.0)<br><br>IG2: Weight before WR = n=81; 91.7 (2.3), Weight change during WR = n=81; -12.6 (0.7), Weight

			**IG2:** W2 group – a walking program targeting 2000kcal expenditure (average 4-6 hrs walking) per week.<br><br>**IG3:** Control group – had no increase in habitual exercise.<br><br>**P:** A nutritionist delivered the small group sessions.	change during WM = n=80; 0.2 (0.9)<br><br>IG3: Weight before WR = n=81; 93.2 (1.6), Weight change during WR = n=81; -13.5 (0.6), Weight change during WM = n=80; 1.7 (0.8)<br><br>Mean weight loss (kg) during WR = 13.5 (0.4) kg<br><br>Mean weight change (kg) during WM = 0.4 (0.5) kg
Glynn et al. 2022;[23] USA; Community<br><br>BES<br><br>Positive	n=206<br><br>IG1 71.8% F, 37.9 (7.9)y, 30.6 (0.2)kg/m^2; IG2 68.9% F, 36.1 (7.7)y, 30.4 (0.2)kg/m^2<br><br>Ethnicity NR<br><br>Retention NR<br><br>Values are estimated means (SEM)	12-wk (84 days)<br><br>Nil FU<br><br>Participants interacted with study staff and nutritionists during 7-day run-in period (7 days before baseline visit) as well as testing periods. No further advice was given to participants.	The diet plan was based on the Diabetic Exchange List (Exchange Diet) as the basis of a meal planning system originally designed by a committee of the American Diabetes Association and the American Dietetic Association. Both groups were assigned a 500-kcal/d deficit from calculated energy needs via an exchange-based diet plan and were advised on guidelines for physical activity (2.5 h/wk of moderate to vigorous-intensity exercise).<br><br>**IG1: (HPF – high protein and fiber)** Participants in the HPF intervention group consumed a commercially available dietary supplement shake containing 17 g protein and 6 g fiber.<br><br>**IG2: (LPF - low protein and lower fiber)**<br>The LPF control group consumed a maltodextrin-based placebo supplement that contained 1 g protein and 3 g fiber. The LPF was matched for caloric content, color, flavor palatability, and vitamin and mineral fortification as in the HPF supplement.<br><br>Both groups were instructed to consume their respective shake preloads 30 min prior to both breakfast and lunch.<br><br>**P:** A qualified nutritionist provided instructions on completing 3-day food record, bowel habits diary and Stanford 7-Day Physical Activity Recall Questionnaire.	BMI (kg/m^2)<br>IG1: n=103; B = 30.4 (0.1), Day-28 = 29.8 (0.1), Day-56 = 29.5 (0.1), Day-84 = 29.3 (0.1)<br><br>IG2: n=103; B = 30.4 (0.1), Day-28 = 30.0 (0.1), Day-56 = 29.9 (0.1), Day-84 = 29.8 (0.1)<br><br>Values are estimated EMM (SE). N for each timepoint not provided
Goodrick et al. 1998;[24] USA; Community<br><br>BES	n=219, 100% F	6-mo<br><br>12-mo FU	**IG:** Dieting treatment (DT) – diet program based on LEARN manual, restricted to 40g fat/day, weight loss target of 1 lbs/week and home-based walking program (4-5 hrs/week).	BMI (kg/m^2)<br>IG: n=65, B = 33.50 (3.46), Post = 33.29 (4.03), 12-mo FU = 34.03 (4.14)

Neutral	25-50y, 40 (6.3)y, 33 (3.4)kg/m^2, White 85%, Black 8%, Hispanic 7%<br><br>Retention NR	24x 1 hour group sessions weekly for 6 months, followed by 26x biweekly maintenance classes for 12 months.	**IG-WN:** Non-dieting treatment (NDT) – no diet is encouraged; participants start intervention with a psychotherapeutic phase to address self-esteem and body issues and breaking of diet cycles. Participants attended the home-based walking program (4-5 hrs/week).<br><br>**CG:** Wait-list control – participants were assessed at baseline and after 6 months, no contact occurred during the period and afterwards they were offered a free course of treatment.<br><br>**P:** All classes were facilitated by dietitian and psychotherapist.	IG-WN: n=62; B = 33.16 (3.21), Post = 33.67 (3.68), 12-mo FU = 33.62 (4.34)<br><br>CG: n=58, B = 32.33 (2.97), Post = 32.47 (3.35)
Jeffery et al. 1998;[25] USA; Community<br><br>Gormally Binge Eating Questionnaire<br><br>Neutral	n=196, 84% F<br><br>25-55y<br><br>IG1 41.5 (1.3)y, 31.5 (0.3)kg/m^2, White 71%; IG2 41.0 (1.3)y, 31.4 (0.3)kg/m^2, White 88%; IG3 42.6 (1.4)y, 31.5 (0.4)kg/m^2, White 73%; IG4 40.7 (1.4)y, 30.6 (0.4)kg/m^2, White 86%; IG5 40.0 (1.3)y, 31.4 (0.3)kg/m^2, White 82%<br><br>R=6-mo = 87%, 18-mo = 78%	18-mo<br><br>Nil FU<br><br>All groups: 24x group sessions weekly for 24 weeks, then monthly group sessions until the end of 18 months<br><br>IG1-4: additional 3x supervised group walking sessions per week	All participants received Standard behavior therapy (SBT) - participants attended counselling session in groups of 20. Participants were assigned to a calorie goal of 1000 kcal/day if they weighed less than 91 kg or 1500 kcal/day if they weighed more than 91 kg, 20% fat intake (22g/day for 1000 kcal and 33g/day for 1500 kcal). Menus for 5 breakfast and 5 dinner meals were provided including shopping lists. Participants were instructed to walk or bike 250 kcal/week and gradually increase to 1000 kcal/week.<br><br>**IG1:** Supervised exercise group – participants received same dietary and behavioral counselling as the SBT group, instructed to 1000kcal/week of walking exercise. To help participants to achieve this goal, they were provided with 3 supervised walking sessions per week.<br><br>**IG2:** Trainer group – participants received the SBT treatment and 3x supervised walking sessions per week, however, each small group (3-4 people) were assigned a personal trainer who stayed with them for the entire study period to supervise the walking sessions and send reminder text-messages and do make-up sessions.<br><br>**IG3:** Incentive group – participants received the SBT treatment and 3x supervised walking sessions per week, they were given financial award for attendance. They were paid $1 per walk for the first 25 walks, $1.50 for the next 50 walks, $2 for the next 50 walks, and $3 for the remainder.	BMI NR<br><br>Weight change (kg)<br>IG1: Base-6-mo = -6.0 (1.1), 6-mo-18-mo = +2.9 (0.9), Base-18-mo = -3.8 (1.3)*<br><br>IG2: Base-6-mo = -5.6 (1.0), 6-mo-18-mo = +3.4 (0.8), Base-18-mo = -2.9 (1.1)*<br><br>IG3: Base-6-mo = -6.7 (1.1), 6-mo-18-mo = +2.1 (0.8), Base-18-mo = -4.5 (1.2)*<br><br>IG4: Base-6-mo = -7.9 (1.1), 6-mo-18-mo = +2.2 (0.9), Base-18-mo = -5.1 (1.3)*<br><br>IG5: Base-6-mo = -8.3 (1.0), 6-mo-18-mo = +0.9 (0.8), Base-18-mo = -7.6 (1.1)*

			**IG4:** Trainer + incentive group – this group received the SBT treatment, supervised walks with a personal trainer and financial award for attendance.<br><br>**IG5:** Standard behavior therapy (SBT) alone<br><br>**P:** Group sessions were led by trained interventionists with advanced degrees in nutrition or the behavioral sciences.	
Jospe et al. 2017;[26] New Zealand; Community<br><br>EDE-Q<br><br>Positive	n=250, 62% F<br><br>IG1 46.1 (11.4)y, 33.2 (4.8)kg/m^2; IG2 44.4 (10.2)y, 33.5 (4.5)kg/m^2; IG3 40.6 (9.9)y, 33.0 (4.1)kg/m^2; IG4 40.7 (10.8)y, 33.0 (4.3)kg/m^2; IG5 46.7 (11.4)y, 32.3 (4.3)kg/m^2<br><br>NZEO 88%, Maori 7.2%, Pacific 2.8%, Asian 2%<br><br>R=68.4%	12-mo<br><br>Nil FU<br><br>All groups: initial 30-45 mins face-to-face session<br><br>IG1, IG2 and IG5: no physical contact from researchers<br><br>IG3: monthly 10-15 mins individual meetings with researchers<br><br>IG4: 2x follow-up sessions at the clinic only in the first month	Participants were advised to follow their chosen diet (Mediterranean diet, Paleo diet or Intermittent fasting) and exercise plan (30 mins of moderate-intensity exercise at least 5 days/week or 5-15mins of high-intensity interval training 3 times/week). Participants were then randomized to 4 monitoring groups:<br><br>**IG1:** Daily self-weighing group (weight monitoring) – participants were asked to weigh themselves at the same time every day and then texted their weight to researchers or entered it to an online database. They received monthly emails providing feedback and encouragement.<br><br>**IG2:** MyFitnessPal group (diet monitoring) – participants were asked to track their dietary intake, using the MyFitnessPal app, every day for the first month and for 1 week every month from months 2-12.<br><br>**IG3:** Brief support group (face-to-face monitoring) – participants attended monthly individual meetings for weight measurements and discussion of ongoing successes and challenges.<br><br>**IG4:** Hunger training group (hunger monitoring) – participants were required to test their capillary blood glucose with a portable glucometer every time they wanted to eat for the first 2 weeks. If their blood glucose was less than or equal to their fasting blood glucose cut-off they were able to eat, otherwise, they were advised to retest in an hour if still hungry. For the remainder of the study blood glucose testing was optional, but participants were asked to complete an all-year round booklet recording perceived intensity of hunger.<br><br>**IG5:** No monitoring group. Diet alone<br><br>**P:** All groups received an initial face-to-face session dietitian and medical doctor.	BMI (kg/m^2)<br>IG1: B = n=48; 33.4 (4.9), 6-mo = n=48; 32.4 (4.9), 12-mo = n=39; 32.1 (5.5)<br><br>IG2: B = n=42; 33.1 (4.4), 6-mo = n=40; 32.2 (4.8), 12-mo = n=36; 32.2 (4.8)<br><br>IG3: B = n=38; 32.6 (3.6), 6-mo = n=36; 32.0 (3.8), 12-mo = n=32 31.9 (4.4)<br><br>IG4: B = n=37; 32.6 (4.4), 6-mo = n=36; 31.1 (4.0), 12-mo = n=28; 30.8 (4.4)<br><br>IG5: B = n=44; 32.0 (4.1), 6-mo = n=44; 30.9 (4.3), 12-mo = n=36; 30.9 (4.6)

Kalarchian et al. 2013;[27] USA; Community<br><br>EDE<br><br>Positive	n=240, 86.7% F<br><br>45.2 (11)y, 47.9 (6.7)kg/m^2, White 82.9%, Hispanic Latino 0.8%<br><br>R=IG1 = 85.1%, IG2 = 70.6%	24-wk<br><br>Nil FU<br><br>IG1: Participants received a combination of face-to-face and tele-health individual counselling sessions (total of 12 individual, face-to-face sessions and 12 telephone contacts).<br><br>IG2: Most patients were seen once a month for 6 months, either in group sessions provided by bariatric surgery program or as arranged individually (not by study staff).	**IG1:** (Lifestyle Program) Participants aimed for 1200-1400kcal/day within a balanced diet (in context of bariatric surgery nutritional guidelines). Participants received nutrition education and instruction to take a daily multivitamin.<br>Goal of 30 mins of exercise 5 times/week. Participants received support for self-monitoring and goal setting.<br><br>**IG2:** (Usual Care) Participants received synopsis of information provided to IG1. They were instructed to complete a non-standardized, physician advised, diet and exercise program.<br><br>**P:** Interventionists received training in behavioral and surgical management of obesity and regular supervision.	BMI NR<br><br>Weight (kg)<br>IG1: B = n=103; 130.3 (20.1), Post = n=103; 121.9 (19)<br><br>IG2: B = n=84; 128.9 (20.1) Post = n=81; 125.1 (19.1)
Keränen et al. 2009;[28] Lifestyle Intervention Treatment Evaluation (LITE) Study; Finland; Community<br><br>BES<br><br>Positive	n=82, 72%F<br><br>18-65y<br><br>IG1 49 (9)y, 35 (5)kg/m^2; IG2 50 (8)y, 35 (5)kg/m^2<br><br>Ethnicity NR<br><br>R=59.7%	18-mo<br><br>Nil FU<br><br>IG1: 10x counselling sessions with nutritionist biweekly for 20 weeks<br><br>IG2: 2x counselling sessions with nurse at a 2 week interval, later 2 more measurement visits at visits 6 and 10 as the IG group	**IG1:** Intensive counselling group – participants attended biweekly individual and group counselling sessions with nutritionist to improve diet and eating behaviors.<br><br>**IG2:** Short-term counselling group – participants attended 2 counselling sessions with a nurse specializing in obesity to provide dietary counselling at the beginning of the intervention. No further counselling sessions were provided for the rest of the intervention.<br><br>**P:** Counselling was conducted by a clinical nutritionist.	BMI NR<br><br>Weight loss (kg)<br>IG1: 1-mo-6-mo = -5.0 (5.7), 1-mo-18-mo = -2.6 (6.0)<br><br>IG2: 1-mo-6-mo = -2.4 (2.5), 1-mo-18-mo = -0.7 (3.5)
LaRose et al. 2014;[29,30] USA; Community<br><br>EDDS<br><br>Neutral	n=178, 53% F<br><br>52.0 (8.6)y, 35.0 (4.4)kg/m^2, Non-Hispanic White 90%<br><br>Retention NR	18-mo<br><br>Nil FU<br><br>Both groups attended weekly groups for the first 6 months, biweekly groups for the next 12 months.	Both groups were instructed to weigh daily, prescribed a low-calorie diet (i.e., 1200–1500 kcals/day, ≤30% kcals from fat) and ≥200 mins/week of moderate intensity exercise. The lifestyle intervention had 3 components: a cognitive behavioral intervention, a diet prescription, and a physical activity prescription. The 3 components are designed to assist participants with a developing a healthy lifestyle to achieve weight loss of 10%.<br><br>**IG1:** (Lifestyle Intervention with limited dietary variety) This group was limited to only 2 self-selected non-nutrient-dense, energy-dense foods (NND-EDFs). Participants were not given instructions regarding any specific amount of the	BMI NR<br><br>Weight (kg)<br>12-mo: Participants weighing more than daily (DW Group) = n=112; -13.8±8.6 kg<br><br>Participants weighing less than daily (<DW Group) = n=43; -9.4±7.4 kg<br><br>18-mo: Participants weighing more than daily (DW Group) = n=105; -13.4±9.4 kg

			chosen NND-EDFs to consume or the frequency of consumption of these foods.<br><br>**IG2:** (Standard Lifestyle Invention)<br><br>**P:** The meetings were led by an experienced research interventionist (either master or doctoral level) with expertise in nutrition, exercise physiology, and behavior modification and were delivered in a research setting.	Participants weighing less than daily (<DW Group) = n=49; -7.4±7.8 kg
Mason et al. 2019;[31,32] USA; Community<br><br>BES<br><br>Positive	n=439, 100% F<br><br>58.0 (5.0)y, 30.9 (40.0)kg/m^2<br><br>Ethnicity NR<br><br>R=90.9%	12-mo<br><br>Nil FU	**IG1:** Dietary Weight Loss – 1200–2000 kcal/day based on participants' baseline weight, with < 30% calories from fat. Included: Individual and group sessions were designed to develop skills for weight loss including goal setting, self-monitoring, coping strategies and problem solving, but were not designed to specifically address disordered eating.<br><br>**IG2:** Moderate-to-Vigorous Intensity Aerobic Exercise – Aerobic exercise progressed to 45 min of moderate-to-vigorous intensity exercise on 5 days/ week.<br><br>**IG3:** Combined Diet and Exercise – Received separate sessions and were instructed not to discuss diet during supervised exercise.<br><br>**CG:** No Intervention – Participants instructed not to change their diet or exercise habits for 12 months.<br><br>**P:** Dietary advice delivered by registered dietitians (RD). Exercise sessions delivered by a certified exercise physiologists.	BMI (kg/m^2)<br>IG1: B = n=118; 31.0 (3.9) (sig diff from control), 12-mo = n=118; 30.5 (4.1) (sig diff from IG2) % change sig diff from IG4<br><br>IG2: B = n= 117; 30.7 (3.7) (sig diff from control), 12-mo = n=117; 29.9 (3.8) (sig diff from IG1) % change sig diff from IG4<br><br>IG3: B = n=116; 31 (4.3) (sig diff from control) 12-mo = n=116; 27.6 (4.5) (sig diff from IG2) % change sig diff from IG2<br><br>CG:<br>Baseline = n=87; 30.7 (3.9)<br>12 months = n=87; 30.5 (4.1)
Martin et al. 2019;[33,34] E-MECHANIC; USA; Community<br><br>MAEDS<br><br>Neutral	n=198, 72.5% F<br><br>IG1 48.3 (11.2)y, 31.4 (4.6)kg/m^2, Caucasian 66.1%, African American 33.9%, Hispanic/other 0%; IG2 48.7 (12.4)y, 30.6 (4.4)kg/m^2, Caucasian 72.6%, African American 23.5%, Hispanic/other 3.9%; IG3 49.5 (10.8)y, 32.3 (4.8)kg/m^2, Caucasian 62.3%, African American 34.4%, Hispanic/other 3.3%.<br><br>R=89.9%	24-wk<br><br>Nil FU<br><br>Participants across IG1 and IG2 had flexibility to choose the number of days per week that they would like to exercise (between 3 and 5 days/week).	**IG1:** 8KKW – prescribed exercise that reflected recommendations for general health (8 kcal/kg of body weight/wk or ~700kcal/wk)<br><br>**IG2:** 20KKW – prescribed a higher exercise dose that is recommended for weight loss and weight loss maintenance (20 kcal/kg of body/wk or ~1760 kcal/wk)<br><br>**IG3:** Received multimedia health information twice weekly by text messaging or e-mail throughout the study period. The information covered many topics, including stress management, benefits of eating fruit and vegetables.<br><br>**P:** Supervised and monitored by trained professionals	Weight change (kg) at 24-wk (values are least-squares means with 95% CIs)<br><br>IG1 = n=59; -0.4 (-1.2, 0.4)*<br><br>IG2 = n=51; -1.6 (-2.4, -0.8)*<br><br>IG3 = n=61; -0.2 (-1.0, 0.6)*<br><br>(p=0.02, means differ significantly)

Mensinger et al. 2016;[35] USA; Community<br><br>EDE-Q<br><br>Positive	n=80, 100% F<br><br>IG-WN 39.83 (4.34)y, 37.42 (0.57)kg/m^2, White Non-Hispanic 93%, Hispanic 8%, African American/Black Non-Hispanic 0%; IG 39.35 (3.91)y, 38.56 (0.65)kg/m^2, White Non-Hispanic 95%, Hispanic 3%, African American/Black Non-Hispanic 3%<br><br>R=Post = 90%, 18-mo FU = 50%	6-mo<br><br>18-mo FU<br><br>6 months of facilitator-guided weekly group meetings of 20 women using structured manuals.	**IG-WN:** Weight-Neutral Program – used the HUGS Program for Better Health which incorporated the main components of Health at Every Size®. The key aim of the program was to help participants break away from a dieting mindset that often leads to a vicious cycle of bingeing and guilt due to an overly restrictive lifestyle. The program taught size acceptance, self-care, and strategies to recognize and respond to physiological signs of hunger and satiety to determine food intake. HUGS did not directly address internalized weight stigma.<br><br>**IG:** Conventional Weight-Management Program – used the LEARN Program for Weight Management. Weight loss an explicit goal and<br>focused on food intake levels based on external prescriptions and calorie restriction. Participants maintained food diaries and physical activity logs between the scheduled program meetings each week, participants were expected to complete exercises from the manual.<br><br>**P:** IG-WN was delivered by a psychotherapist<br>and fitness professional. IG was delivered by a registered dietician.	BMI NR<br><br>Weight NR
Moss et al. 2017;[36] Canada; Community<br><br>EDE-Q<br><br>Positive	n=135, 78% F<br><br>45.16 (11.30)y, 33.58 (6.26)kg/m^2, Caucasian 93.3%<br><br>IG1 45.56 (9.78)y, 33.78 (5.98)kg/m^2, Caucasian 95.77% Other (Asian, East Indian, Hispanic) 4.29%; IG2 44.67 (12.91)y, 33.37 (6.58)kg/m^2, Caucasian 90.63%<br>Other (Asian, East Indian, Hispanic) 9.38%	12-wk<br><br>6-mo FU<br><br>The semi-structured interviews were 45-min interventions. Participants completed an interview at the beginning of the BWLP program, at the 12th week of the program and at follow up.	All participants completed the behavioral weight-loss program (BWLPs). This BWLP emphasizes gradual, sustainable weight loss and lifestyle changes. The program consists of three core components: (1) Nutrition: individualized guidelines for healthy eating, based on the Canada Food Guide, were developed for each participant, (2) Physical activity: group exercise classes focused on fat loss, strength training, and development of endurance and flexibility, and (3) Behavior change: behavioral strategies including self-monitoring, goal-setting, and formulating action plans to achieve goals were taught in classroom sessions.<br><br>The first author delivered all the motivational interviewing and control sessions, both for practical reasons and to help control for possible therapist effects. Therapist training consisted of over 20 h of readings, video, role play, discussions of MI principles and strategies, and a total of 8 days of workshop training facilitated by members of the Motivational Interviewing Network of Trainers. Ongoing supervision was provided by a doctoral-level clinical psychologist throughout.	BMI (kg/m^2) mean (SE)<br>IG1: B = 33.78 (0.72), Post = 32.27 (0.16), 6-mo FU = 32.96 (0.16)<br><br>IG2: B = 33.37 (0.81), Post = 32.64 (0.16), 6-mo FU = 33.11 (0.16)

			**IG1:** Motivational Interviewing (MI) Intervention Group – The MI protocol included the following components: (1) eliciting concerns about weight, (2) exploring ambivalence, (3) assessing importance and confidence for change, (4) writing a decisional balance, (5) bolstering self-efficacy, (6) looking towards the future, and (8) eliciting ideas for possible changes participant could make to work towards weight loss.<br><br>**IG2:** Attention Control Intervention – The attention control interview was a semi-structured interview addressing health history, weight history, diet history, and dietary and physical activity habits. Most questions were drawn from the TrymGym intake application. It was designed to be structurally equivalent to the MI session in length of session, timing of sessions, and treatment modality. The goal was to provide a pseudo-intervention that controlled for factors common to attending treatment (e.g., attending treatment sessions, having personal contact with a therapist, discussing weight-related issues).<br><br>**P:** The BWLP was delivered by a team of health care practitioners including dietitians, kinesiologists, and fitness instructors via both classroom sessions and exercise sessions.	
Muggia et al. 2014;[37] Italy; Outpatient<br><br>BITE short version (16 items)<br><br>Positive	n=163, 57% F<br><br>44.82 (10.98)y, 32.22 (3.64)kg/m^2<br><br>Ethnicity NR<br><br>R=6-mo = 69.9%, 12-mo FU = 47.8%	6-mo<br><br>12-mo<br><br>All participants received 30-minute visit every 3 months until 12 month and every 6 months from 12 to 24 month, for a total of five visits in the first year, and of two visits in the second year.	Both interventions involved a 5-600 kcal/day calorie deficit (10-20% of total calories as protein, 55-60% as carbohydrates, 25-30% as lipids).<br><br>Participants were given a booklet containing information on food groups, and the use of portions reported in the Mediterranean food pyramid.<br><br>**IG1:** Standard Care – Participants followed a low-calorie diet.<br><br>**IG2:** Group CBT – In addition to a low-calorie diet, participants attended in small groups a series of 7 meetings (90 mins each).<br><br>**P:** Intervention was led by a physician, a therapist psychologist, and a dietician.	BMI NR<br><br>Weight (kg)<br>IG1: B = 85.58 (12.41)<br><br>IG2: B = 84.44 (13.54)<br><br>6-mo, mean weight loss = 6.0%.<br><br>12-mo FU, mean weight loss = 7.44%.
Pacanowski et al. 2014; [38,39] Keep It Off; USA; Community	n=419, 81.6% F<br><br>19-70y, 46.5 (10.8) y, 28.5	24-mo<br><br>Nil FU	**IG1:** Guided maintenance phone coaching intervention – Participants were provided with a Keep It Off coursebook on weight-loss maintenance and regular 1-on-1 phone coaching with health expert. No specific calorie or fat	BMI NR<br><br>Weight NR

Self-report frequency of binge eating, using 3 items from the Eating Disorder Diagnostic Scale<br><br>Neutral	(4.9)kg/m^2, Non-Hispanic white 91.4%<br><br>Retention NR	(10x 20 mins 1-on-1 core phone coaching fortnightly, then 8x 10-15 mins phone coaching monthly, then 6x 10-15 mins phone coaching bi-monthly<br><br>Frequency of contact (self-directed): 2x 20 mins 1-on-1 phone course at the beginning of the intervention	reduction goal was prescribed, participants were expected to self-monitor food intake and exercise.<br><br>**IG2:** Self-directed maintenance phone coaching intervention – Participants were provided with a Keep It Off coursebook on weight-loss maintenance and a two-session phone course with health expert. No specific calorie or fat reduction goal was prescribed, participants were expected to self-monitor food intake and exercise.<br><br>**P:** The Keep It Off phone coaches who conducted the intervention calls were masters' and/or bachelor's level individuals with expertise in nutrition, physical activity, and weight loss, and behavior change methods.	
Radin et al. 2020;[40,41] USA; Community<br><br>BES<br><br>Positive	n=194, 80% F<br><br>46.98 (12.71)y, 35.47 (3.62)kg/m^2, White 59.3%, Black 12.9%, Latino 11.9%, Asian/Pacific Islander 9.8%, Native American 1.0%, Other 5.2%<br><br>Retention NR	5.5-mo<br><br>6, 12-mo FU<br><br>12 weekly 2–2.5-hour group evening sessions, 3 bi-weekly sessions, 1 follow-up session 4 weeks later, and an all-day weekend session near the 8th week of the program.	Both intervention groups received the same dietary and exercise guidelines (e.g., goal of reducing daily food intake of their choice by 500 calories and increasing activity).<br><br>**IG1:** Diet-exercise intervention with a mindfulness component – This intervention incorporated both specific mindful eating techniques as well as general mindfulness techniques (for stress management and emotion regulation). This was adapted from the Mindfulness-Based Eating Awareness Training program. The aim was to promote awareness and self-regulation of physical hunger, stomach fullness, taste satisfaction, food cravings, and other triggers for eating in the context of reduced caloric intake.<br><br>**IG2:** Diet-exercise intervention without a mindfulness component – the participants received additional educational content, including information about nutrition and physical activity. It also included cognitive behavioral therapy tools and instruction in progressive muscle relaxation for stress management.<br><br>**P:** IG1 mindfulness intervention was led by one of three mindfulness meditation instructors and co-led by the same registered dietitian (except for one cohort). IG2 was led by one of three registered dietitians masked to study hypotheses.	BMI NR<br><br>Weight (kg)<br>IG1 lost an average of 1.9 kg more than IG2 at 18 months but this difference was not statistically significant (95% CI: −4.5, 0.8 kg).
Raman et al. 2018;[42] Australia; Community<br><br>EDE-Q<br><br>Positive	n=80, 86% F<br><br>IG1 40.6 (7.0)y, 40.3 (7.8)kg/m^2; IG2 42.2 (8.8)y, 39.2 (7.4)kg/m^2	7-9-wk<br><br>All participants received 3 weeks of Behavioral Weight Loss Treatment (BWLT)	The BWLT targeted diet and exercise through behavioral modification techniques. After the BWLT, the participants were then randomly allocated into two groups:<br><br>**IG1:** Cognitive Remediation Therapy for Obesity (CRT-O) – Program was delivered face-to-face and consisted of	BMI (kg/m^2)<br>IG1: B = n=42; 40.3 (7.7), Post = n=41; 38.9 (7.6), 3-mo FU = n=37; 38.3 (7.6)

	Ethnicity NR<br><br>Retention NR	group program 1x week for 90 mins.<br><br>4-6 weeks of either IG1 or IG2.<br><br>3-mo FU	mental exercises aimed at improving cognitive strategies, thinking skills and information processing through practice.<br><br>**IG2:** Participants were instructed to continue their weight loss efforts but were not given further instructions.<br><br>**P:** CRT-O was delivered by a clinical psychologist.	IG2: B = n=38; 39.2 (7.4), Post = n=31; 39.7 (8.4), 3-mo FU = n=26; 38.8 (8.4)
Ramirez et al. 2001;[43] USA; Community<br><br>EDE-Q<br><br>Neutral	n=65, 78.4% F<br><br>19-63y, 44.0 (9.7)y, 33.78 (5.13)kg/m^2<br><br>Ethnicity NR<br><br>R=74%	16-wk<br><br>3-mo & 1y FU<br><br>IG1: 12x 2-hour weekly visits with dietitian and psychologist, then 4x 1hr weekly visits with dietitian<br><br>IG2: 16x 1-hour weekly visits with dietitian	**IG1:** Weight control with body image therapy group – participants received the same weight-control intervention as the control group, with added cognitive behavioral body image therapy based on Rosen's program. Participants attended 2 hrs sessions, seeing dietitian for weight control in the first hour then psychologist for the second hour. After 12 weeks, participants were only provided 1 hr weight control sessions with dietitian.<br><br>**IG2:** Weight control group – a nutrition and behavioral management intervention based on LEARN program.<br><br>**P:** Sessions led by registered Dietitian	BMI NR<br><br>Weight (kg)<br>IG1: B = n=38; 101.08 (22.9), Post = 92.31 (21.7), 3-mo FU = 91.79 (21.9), 1-y FU = 95.43 (23.9)<br><br>IG2: B = n=27; 91.03 (13.2), Post = 81.78 (11.4), 3-mo FU = 81.95 (11.6), 1-y FU = 87.64 (13.1)<br><br>Weight loss (%)<br>IG1: B = 8.66 (4.2), 3-mo FU = 9.06 (6.6), 1-y FU = 5.90 (7.9)<br><br>IG2: B = 10.46 (5.2), 3-mo FU = 11.06 (5.6), 1-y FU = 4.69 (8.5)
Rapoport et al. 2000;[44] UK; Community<br><br>BES<br><br>Positive	n=84, 100%F<br><br>18-65y; IG1 49 (10)y, 35.4 (6.3)kg/m^2, White 81%, Afro-Caribbean/African 14%, Asian 5%; IG2 46 (12)y, 35.3 (5.6)kg/m^2, White 68%, Afro-Caribbean/African 21%, Asian 11%<br><br>R=84%	10-wk<br><br>6-mo & 12-mo FU<br><br>10x 2 hours weekly group consultation with dietitian and psychologist	**IG1:** Modified cognitive-behavioral treatment (M-CBT) group – no weight loss goal was set or promised as the aim of the intervention was weight management through lifestyle changes using CBT strategies. Diet goal (50-55% carbohydrates, 35% fat, 15% protein), participants were not given energy intake limits, but had average of 1800 kcal/day. Participants were instructed to start a walking program and increase by 5 mins each week.<br><br>**IG2:** Standard cognitive-behavioral treatment (S-CBT) – weight loss was encouraged in this intervention, participants were advised on 1200 kcal/day energy deficit (50-55% carbohydrates, 35% fat, 15% protein), weight loss target of 0.5-1.0 kg/week, and physical activity was addressed through motivational interviewing.<br><br>**P:** Treatment delivered by a State Registered Dietitian and a health psychologist, who had received training and supervision in CBT methods.	BMI (kg/m^2)<br>IG1: B= n=31; 35.2 (6.1), Post = n=31; 34.7 (5.7), 6-mo FU = n=28; 34.2 (5.3), 12-mo FU = n=30; 34.5 (5.5)<br><br>IG2: B = n=32; 35.5 (5.7), Post = n=32; 34.1 (5.8), 6-mo FU = n=31; 34.0 (6.0), 12-mo FU = n=28; 34.2 (6.7)
Raynor et al. 2006;[45] USA; Community	n=30, 90% F	8-wk<br><br>Nil FU	All participants were given a daily caloric goal of 1200 to 1500 kcal/day, depending on baseline body weight, with 20% calories from fat, for weight loss.	BMI NR<br><br>Weight (lbs)

BES<br><br>Neutral	49.5 (9.9)y, 32.2 (3.3)kg/m^2, Caucasian 90%	Time of group sessions NR	All participants kept a food diary throughout the intervention and were provided weekly feedbacks. Participants were also asked to gradually increase moderate-intensity physical activity to at least 150 min/week.<br><br>All participants attended weekly group sessions and were taught behavioral and cognitive skills, including self-monitoring, stimulus control, problem-solving, social support and assertiveness training, goal setting, cognitive restructuring, and relapse prevention.<br><br>**IG1:** Reduced Snack Variety Group – Participants were instructed to choose one highly liked, commonly eaten snack food to continue to include in the diet. The participants were then instructed to restrict their snack food consumption to this one chosen food during the 8 weeks in unlimited amounts at least 4x/week.<br><br>**IG2:** No Variety Limit for Snacks Group – The participants consumed snack food <1 per day however had no limitation on snack variety.<br><br>**P:** Sessions run by clinical psychologist	Mean Weight Loss = -7.4 (5.8)lb
Reiger et al. 2017;[46,47]<br>Australia; Outpatient<br><br>BES<br><br>Positive	n=201, 73.6% F<br><br>IG1 46.93 (12.01)y, 37.64 (6.61)kg/m^2; IG2 47.1 (11.0)y, 37.78 (6.02)kg/m^2<br><br>Ethnicity NR<br><br>R=47%	12-mo<br><br>12-mo FU<br><br>All participants received 26, 90 mins group sessions comprised of 8 weekly, 16 fortnightly, and 2 monthly sessions over 12 months, with 6-8 patients per group.	The program used CBT and motivational interviewing to teach participants cognitive-behavioral skills for dietary modification and increasing physical activity and included both a weight loss phase (the initial 8 months) and a weight maintenance phase (the final 4 months).<br><br>The initial sessions education regarding the recommended caloric intake, rate of weight loss and structure of eating as well as instituting daily self-monitoring of eating and physical activity.<br><br>**IG1:** Participants followed the cognitive behavior therapy weight program alone (CBT-A)<br><br>**IG2:** Participants followed the cognitive behavior therapy weight program with a support person (CBT-SP). The support people underwent training to enable them to become skilled in eliciting self-motivation for weight control from the patients.	BMI (kg/m^2)<br>IG1: B = 37.71 (6.64)*, Post = 35.75 (7.03)*, 12-mo FU = 36.49 (6.61)<br><br>IG2: B = 36.84 (5.01)*, Post = 34.25 (4.70)*, 12-mo FU = 35.07 (5.19)

			**P:** Interventions were conducted by five therapists with postgraduate degrees in clinical psychology.	
Schyns et al. 2020;[48,49] Netherlands; Community<br><br>EDE-Q + semi-structured clinical interview<br><br>Neutral	n=45, 100% F<br><br>44.26 (10.42)y, 33.68 (4.32)kg/m^2<br><br>Ethnicity NR<br><br>R=87%	1-mo<br><br>3-mo FU<br><br>All participants received eight individual therapy sessions of approx. one hour that took place during approximately one month (two sessions per week).	**IG1:** Cue Exposure – Participant's favorite foods and individual expectancies were used for the exposure sessions, including one most favorite food item (personal-exposed food item). Participants were instructed to do daily homework exposure exercises.<br><br>**IG2:** Lifestyle+ - Participants received dietary advice on a healthy lifestyle, mindfulness, power posing and psycho-education on body image. Daily homework exercises consisted of mindfulness and exercises related to the content of the previous session. During telephone sessions, the homework exercises were evaluated.<br><br>**P:** Both interventions were delivered individually and conducted by PhD students and clinical psychology students who were obtaining their master degree.	BMI NR<br><br>Weight loss (%)<br>IG1: B = NR, 1-mo = -1.8%, 3-mo FU = -2.1%<br><br>IG2: B = NR, 1-mo = -0.6%, 3-mo FU = +0.2%
Simpson et al. 2015;[50] UK; Outpatient and Community<br><br>EDE-Q<br><br>Positive	n=170, 83% F<br><br><30y 9.6%, 30-59y 60.8%, ≥60y 29.5%, 34.2 (5.86)kg/m^2, White 94.6%, Non-White 5.4%<br><br>R=84%	12-mo<br><br>Nil FU	**IG1:** An Intensive Intervention Arm –<br>Participants received six one-to-one individually tailored MI (motivational interviewing) sessions, delivered by experienced MIPs (motivational interviewing practitioner). These sessions were delivered face to face, approximately fortnightly for 3 months, and lasted about 60 mins. During the final 9 months of the intervention, participants received monthly MI telephone calls lasting approximately 20 mins.<br><br>**IG2:** A Less Intensive Intervention Arm –<br>Participants received two face-to-face tailored MI sessions 2 weeks apart and two MI-based telephone calls at 6 and 12 months only.<br><br>**IG3:** The control group were given an information pack also sent to participants in both intervention arms. The content of the information pack was based on useful resources for weight loss and healthy lifestyle, and advice on WLM (weight loss maintenance). Participants in all arms were able to access usual care, for example attending a slimming club.<br><br>**P:** All intervention staff (MIPs and group facilitators) were trained as per the appropriate manual.	BMI (kg/m^2)<br>IG1: n=54; B = 34.4 (6.19), Post = 33.3 (6.50)<br><br>IG2: n=54; B = 34.8 (6.20), Post = 33.4 (6.03)<br><br>IG3: n=58; B = 33.3 (5.19), Post = 33.0 (5.22)
Smith et al. 2018;[51] USA; Community<br><br>BES	n=40, 100% F<br><br>50-70y, 58.46 (4.87)y	6-wk<br><br>6-wk, 4-mo, 9-mo & 1y FU	**IG1:** Mindful eating and living (MEAL) group – incorporating mindfulness to eating behavior by increasing awareness and gaining greater control over their eating. The intervention based on the work of Kristellar, includes a	BMI (kg/m^2)<br>IG1: n=18; B = 34.68 (4.26), 6-wk FU = 33.39 (4.55), 4-mo FU =

Positive	IG1 34.68 (4.26)kg/m^2; IG2 38.24 (7.08)kg/m^2<br><br>Ethnicity NR<br><br>R=90%	6x 2 hours weekly group meetings, 10x 1 hour monthly follow-up group meetings	6-week curriculum in groups (up to 20 people) involving group discussion, mindfulness meditation, and group eating exercises. Participants are instructed to listen to a 9 mins breathing meditation CD, eat 1 meal mindfully every day and encourage yoga exercises.<br><br>**IG2:** Active control (CONT) group – providing the same healthcare professional attention and group weight loss treatment sessions as the MEAL group but without the mindfulness techniques, in the sessions the participants discussed food choices, activity levels and caloric goals.<br><br>**P:** IG1 was led by a medical doctor also professionally trained mindfulness-based stress reduction instructor. IG2 was led by an endocrinologist.	32.95 (4.58)*, 9-mo FU = 31.69 (3.64)*, 1y FU = 31.78 (3.85)*<br>*sig dif between baseline and FUs<br><br>IG2: n=18; B= 38.24 (7.08), 6-wk FU = 36.91 (6.59)*, 4-mo FU = 35.79 (6.29)*, 9-mo FU = 35.74 (6.77)*, 1y FU = 36.16 (7.18)*<br>*sig dif between baseline and FUs<br><br>Also sig dif between 6-weeks, 4-months and 9-months.
Steinberg et al. 2014;[52] USA; Community<br><br>Questionnaire for Eating and Weight Patterns Revised<br><br>Mizes Anorectic Cognitions Questionnaire<br><br>Neutral	N=91, 75% F<br><br>18-60y<br><br>IG 43.0 (11.4)y, 33.18 (4.03)kg/m^2, Black 13%, White 77%, Other 10%; CG 44.7 (10.6)y, 31.05 (3.13)kg/m^2, Black 18%, White 71%, Other 11%<br><br>R=98%	6-mo<br><br>Nil FU<br><br>Tailored feedback on self-weighing and weight loss via weekly emails	**IG:** Daily self-weighing group – participants were instructed to weigh themselves using an e-scale daily at the same time, and they would get tailored feedback on their weight loss trend via a graph via weekly emails. Participants also received 22 weekly lessons on behavioral weight loss based off the Diabetes Prevention Program, where they were advised on a 1200-1500 kcal/day energy-deficit and 150-200 mins/week of moderate intensity exercise.<br><br>**CG:** Delayed intervention group – no intervention during the study period, after 6 months participants were provided a modified program to blind participants on the focus of daily weighing.<br><br>**P:** The intervention was delivered via email. Content was derived and adapted from the Diabetes Prevention Program.	BMI NR<br><br>% Weight loss<br>IG: 3-mo = -4.41 (-5.5, -3.3), Post = -6.55 (-7.7, -5.4)<br><br>CG: 3-mo = -0.37 (-1.5, 0.76), Post = -0.35 (-1.5, 0.79)
Tanco et al.1998;[53] Canada; Community<br><br>EDI<br><br>Positive	n=60, 100% F<br><br>IG-WN 39.4 (5.2)kg/m^2; IG 38.7 (5.8)kg/m^2; CG 40.7 (5.5)kg/m^2<br><br>Ethnicity NR<br><br>R=83.3	8-wk<br><br>6-mo FU<br><br>IG1 and IG2: 8x 2 hour weekly meetings	**IG-WN:** Cognitive treatment program (CT) – focused on enhancing emotional well-being, promoting regular physical exercise and non-disordered eating in non-diet approach. Participants were only weighed on weeks 1, 4, and 8, no diet was instructed. Participants attended group sessions focusing on therapeutic, client-centered format.<br><br>**IG:** Standard behavioral weight management program (BT) – weight reduction by reducing fat intake and exercise adoption. Participants were weighed weekly, daily food and exercise records, instructed on a diabetic exchange diet (1200-1500 kcal/day). Participants attended group sessions in a prescriptive psychoeducational format.	BMI (kg/m^2)<br><br>IG-WN: n = 18; B = 39.4 (5.3), Mid = 39.2 (5.2), Post = 38.8 (5.1), n = 12; 6-mo FU = 37.5 (4.9)<br><br>IG: n = 18; B = 38.7 (5.8), Mid = 38.1 (5.8), Post = 37.3 (5.9), n = 9; 6-mo FU = 36.6 (6.4)<br><br>CG: n = 12; B = 40.7 (5.5), Mid = 40.8 (5.5), Post = 41.0 (5.5), 6-mo FU = NR

			**CG:** wait-list control<br><br>**P:** Weekly meetings for both groups were conducted by experienced clinical psychology graduate students.	
Vander Wal et al. 2006;[54] USA; Community<br><br>NESQ<br><br>Neutral	n=61, 72% F<br><br>IG1 44.76 (11.67)y, 38.34 (6.84)kg/m^2; IG2 47.53 (10.35)y, 37.68 (4.63)kg/m^2<br><br>Ethnicity NR<br><br>R=69%	8-wk<br><br>Nil FU<br><br>FU visits were conducted at weeks 2, 4, 6 and 8.	All participants were instructed to use the Kashi^R GOLEAN^R PMR program which includes a ready-to-eat cereal (RTEC) (177.44 mL serving size, 502.42 kJ, 8 g protein, 1 g fat, 10 g fibre), bar (1 bar serving size, 1214.17 kJ, 13 g protein, 6 g fat, 6 g fibre) and shake (325 mL, 962.96 kJ, 15 g protein, 3 g fat, 7 g fibre). Total energy provided from the products was 2,679.55 kJ per day. Participants were instructed to eat the RTEC for breakfast, the bar at mid-morning, and the shake for lunch. Supper was individually planned in consultation with dietitian. All food supplies required to follow the diet were provided by the study.<br><br>**IG1:** Post-dinner snack group (PDS) - participants in this group were instructed to have a standard bowl of RTEC and 2/3 cup of low-fat milk 90 mins after their supper meal.<br><br>**IG2:** No snack group (NS) - no additional instructions were given to this group.<br><br>**P:** Registered dietitians administered the NESQ and delivered instructions to participants. The study physician monitored health status alongside the dietitian.	Mean total BMI (kg/m^2) change: Total = -1.48 (1.23)*; IG1 = n=29; -1.31 (1.10)*; IG2 = n=32; 1.63 (1.34)*
Wadden et al. 1994;[55] USA; Community<br><br>BES<br><br>Neutral	n=49, 100% F<br><br>39.31y, 39.46kg/m^2<br><br>Ethnicity NR<br><br>R=52-wk = 81.6%	78-wk<br><br>Nil FU<br><br>All participants attended weekly group treatment sessions for the first 52 weeks and biweekly sessions for an additional 26 weeks.<br>Session duration was 90 mins with 6-9 participants.	At Week 8, all participants were instructed to exercise. Beginning with 10-20 mins 2-3 times a week (at 40-60% of estimated maximum heart rate). By week 52, participants were to exercise for 20-40 mins 3-5times a week (at 60-70% maximum heart rate).<br><br>**IG1:** Behavior therapy with 1200 kcal/day balanced-deficit diet (BDD) - Participant were prescribed 1,200kcal/day diet for the first 52 weeks of treatment (15-20% of the calories from protein, max 30% from fat).<br><br>**IG2:** Behavior therapy with short term VLCD (VLCD) - Participants were prescribed a 1,200kcal/day diet for the first week and then VLCD diet for weeks 2-17 (420kcal/day). During weeks 18-23 conventional foods were reintroduced and caloric intake was gradually increased to 1000kcal/day. During weeks 24-52, participants increase caloric intake to 1,200kcal/day.	BMI NR<br><br>Mean Weight (kg) Loss<br>IG1: 17-wk = 10.12 (6.20), 48-wk = 15.71 (9.31)<br><br>IG2: 17-wk = 22.61 (6.01), 48-wk = 18.79 (10.19)

			After the 52-week treatment periods, participants undertook a 26-week maintenance phase with biweekly sessions to provide basic "upkeep" skills.<br><br>**P:** Sessions were led by either a doctoral-level clinical psychologist or a psychology graduate student. Nutritional instructions were delivered by a registered dietitian.	
Wadden et al. 2004;[56] USA; Community<br><br>EDE<br><br>Neutral	n=123, 100% F<br><br>44.2 (10.0)y, 35.9 (4.5)kg/m^2, European American 64.2%, African American 35.0%, Hispanic American 0.8%<br><br>Retention NR	40-wk<br><br>25-wk FU<br><br>All participants attended weekly group treatment sessions during the first 20 week and every-other week sessions during weeks 22–40.<br><br>Group sessions were 90 mins, included 7 to 10 participants. After week 40, participants attended follow-up group sessions at week 52 and week 65.	All participants had activity goals which included walking (or other aerobic activity) for 150 min/wk by the end of week 20, with an increase to 180 min/wk by week 40.<br><br>**IG1:** Balanced-deficit diet (BDD) - From week 2 participants were instructed to self-select BDD of 1200–1500 kcal/d, with15% of calories from protein, 30% or fewer from fat, and the remainder from carbohydrate. This dietary regimen, which is based on the Food Guide Pyramid, is recommended by the LEARN Program for Weight Control.<br><br>**IG2:** Meal replacement plan (MR) - From week 2 to week 13, these participants were prescribed a 1000 kcal/d MR plan that consisted of 4 servings/d of a liquid diet (OPTIFAST 800; Novartis Nutrition Co, Minneapolis), combined with an evening meal of a frozen food entree, a serving of fruit, and a green salad. Each serving of the liquid diet provided 160 kcal, with 14 g of protein, 20 g of carbohydrate, and 3 g of fat. Beginning at week 14, participants gradually decreased their consumption of the liquid diet, so that by week 17 they were prescribed a 1200–1500 kcal/d diet of conventional foods, the same as women in the BDD.<br><br>**IG-WN:** Nondieting approach (ND) - Participants in this group were explicitly instructed not to reduce their calorie intake. At week 6, women were encouraged to adopt a new eating plan. It prescribed that they: 1) eat at least every 4 h to avoid becoming hungry; 2) consume whatever foods they desired; and 3) stop eating when they felt full. Participants also received instruction in improving self-esteem and body image, as well as in living more fulfilling lives, regardless of body weight.<br><br>**P:** Sessions were led by a clinical psychologist. A registered dietitian co-led 6 sessions in each of the 3 treatment conditions.	BMI NR<br><br>% weight loss<br>IG1: 20-wk = 7.8 (6.0)*, Post = 8.4 (8.7)*, 25-wk FU = 6.3 (8.3)*<br><br>IG2: 20-wk = 12.1 (6.7)*, Post = 11.5 (8.9)*, 25-wk FU = 8.6 (10.0)*<br><br>IG-WN: 20-wk = 0.1 (2.4)*, Post = 0.8 (3.2), 25-wk FU = + 0.8 (3.4)

Werrij et al. 2009;[57] The Netherlands; Outpatient<br><br>EDE-Q<br><br>Neutral	n=200, 81% F<br><br>19-65y, 45 (12)y, 27.0 to 52.3 kg/m^2, 33.4 (4.6)kg/m^2<br><br>Ethnicity NR<br><br>R=79%	10-wk<br><br>1-y FU<br><br>Both treatments were given by protocol and consisted of 10 weekly sessions of 2 hours each (12 participants maximum).	Each weekly treatment session was divided into two parts. The first hour was dietetic intervention which was the same for both treatment conditions. This consisted of nutritional education, food diaries, and cooking classes. In the second hour of the session, the intervention differed between the two groups.<br><br>In the CDT condition cognitive therapy (CT) was added to the dietetic treatment, whereas in the EDT condition physical exercise was added to the dietetic intervention.<br><br>**IG1:** Cognitive dietetic group treatment (CDT) - After the first hour of dietetic treatment, the second hour involved experimental CDT condition cognitive therapy (CT). The aims of the CT were to identify, challenge, and change dysfunctional cognitions concerning eating, control, weight, and shape, as well as related schemas.<br><br>**IG2:** Physical exercise dietetic group treatment (EDT) - After the first hour of dietetic treatment, the second hour consisted of one hour of supervised low intensity exercise program (gym).<br><br>**P:** Cognitive therapy was performed by fully qualified cognitive behavior therapists. Fully qualified physiotherapists led the physical exercise component. Dietitians conducted dietetic treatment.	BMI (kg/m^2):<br>IG1: B = 33.42 (4.38), Post = 32.06 (4.42), 1-y = 32.07 (4.46)<br><br>IG2: B = 33.29 (4.76), Post = 31.85 (4.63), 1-y = NR
Whitelock et al. 2019;[58] UK; Community<br><br>BES<br><br>Positive	n=107, 74% F<br><br>42y; IG1 35.9 (6.8)kg/m^2, White 92.5%, Mixed/Multiple 1.9%, Asian/Asian British 5.7%, Black/Black British 0%, Other 0%; IG2 35.2 (6.2)kg/m^2, White 94.4% Mixed/Multiple 1.9%, Asian/Asian British 3.7%, Black/Black British 0%, Other 0%<br><br>R=67.3%<br>Note – analyses were completed with imputed missing data.	8-wk<br><br>Nil FU<br><br>All participants received weekly tips via text message that related to content from the dietary advice booklet.	All participants received a standard dietary advice booklet which contained information and tips adapted from British Heart Foundation materials on healthy eating and weight loss.<br><br>**IG1:** Attentive eating smartphone application along with standard dietary advice – The attentive eating application was designed to promote attentive eating by encouraging users to photograph food and drink being consumed and then review this information when making dietary decisions throughout the day. An additional feature of the application that was added for this trial was an audio clip (2.5 min) that users could listen to whilst eating which encouraged mindful eating.<br><br>**IG2:** Standard dietary advice only	BMI NR<br><br>Weight Change (kg)<br>IG1: 4-wk = − 0.7 (2.1), 8-wk = − 1.2 (2.2)<br><br>IG2: 4-wk = −0.7 (2.2), 8-wk = −1.1 (3.0)

			**P:** The researcher (a psychologist with a PhD) then explained the dietary advice booklet and the weekly text tips following a script.	
Williamson et al 2008;[59] CALERIE trial; USA; Community<br><br>MAEDS – Binge Eating, Purgative Behavior, Restrictive Eating<br><br>Neutral	n=48, 56% F<br><br>IG1 39 (1.5)y, 27.9 (0.4)kg/m^2, White 15%, African American 8%, Asian or Latino 2%; IG2 36 (1.6)y, 27.6 (0.5)kg/m^2, White 15%, African American 8%, Asian or Latino 2%; IG3 38 (2.3)y, 27.8 (0.5)kg/m^2, White 17%, African American 8%, Asian or Latino 0%; IG4 37 (2.1)y, 27.9 (0.6)kg/m^2, White 17%, African American 8%, Asian or Latino 0%<br><br>R=IG1 = 100%; IG2 = 100%; IG3 = 91.7%; IG4 = 91.7%; Minimum care = 76.9%; Extended care = 100%	12-mo<br><br>Nil FU<br><br>During the initial 6 months of treatment, all participants meet for individual therapy at least twice per month, and they attended weekly groups.	All participants were provided with food for the first 12 weeks of the study, and they ate a self-selected diet from weeks 13 to 22. During weeks 22 to 24, participants were again provided food.<br><br>Following completion of the intervention, participants in IG1, IG2 and IG3 were randomly assigned to either a minimum care or an extended care condition. All participants who enrolled were instructed to continue their previously assigned "dieting" intervention. Participants in the extended care condition attended a monthly group and a monthly individual session. Participants in the minimum care condition were not contacted unless they requested help.<br><br>**IG1** (Calorie Restriction – CR): 25% calorie restriction of baseline energy requirements<br><br>**IG2** (Calorie Restriction with Exercise – CR + EX): 12.5% calorie restriction plus 12.5% increase in energy expenditure by structured exercise.<br><br>**IG3** (Low-Calorie Diet – LCD): 890 kcal/day liquid diet until 15% of body weight was lost, followed by a weight maintenance diet.<br><br>**IG4** (Weight Maintenance): weight maintenance diet<br><br>**P:** Individual therapy sessions were run by registered dietitians and exercise physiologists. Weekly group sessions were led by a doctoral-level psychologist.	BMI (kg/m^2) – mean (SEM)<br>Extended Care Arm: 6-mo = 24.1 (0.5), 12-mo = 24.3 (0.6)<br><br>Minimal Care Arm: 6-mo = 24.9 (0.5), 12-mo = 25.5 (0.5)
Zwickert et al. 2016;[60] Australia; Community<br><br>BES<br><br>Positive	n=60, 71.7% F<br><br>19-64y, 44.3y, 37.5kg/m^2<br><br>Ethnicity NR<br><br>R=IG1 = 48.4%; IG2 = 48.3%	15-mo<br><br>Nil FU<br><br>IG1: 3 months = weekly group CBT followed by 9 months of intensive technological support (one and two way text and email communication)	Both groups: 12-week CBT, psycho-education and nutritional info, preparing for implementation of eating and physical activity changes, personalized caloric intake targets based on Harris-Benedict equation, tasks aimed at increasing motivation to engage in weight control behaviors<br><br>**IG1:** Technological support was provided to the CBT + ITS group from 0 to 9 months and involved one- and two-way communication between the therapist and participant. Weekly text messages.	BMI (kg/m^2)<br>IG1: n=31; B = 37 (1.2), 3-mo = 35.1 (1.2), 6-mo = 33.9 (1.2), 9-mo = 33.5 (1.2), 15-mo = 34.1 (1.2)<br><br>IG2: n=29; B = 38.1 (1.2), 3-mo = 36.3 (1.2), 6-mo = 35.5 (1.2), 9-mo = 35.5 (1.2), 15-mo = 36.1 (1.3)

		IG2: 3 months = weekly group CBT followed by 6 months one way therapist to patient text message contact. From 6 to 9 months, CBT + MTS participants did not receive any technological support.	**IG2:** Technological support was provided to the CBT + MTS group from 0 to 6 months in the form of one-way therapist-to-patient contact. Participants received a daily text-message, primarily containing CBT weight control strategies.<br><br>**P:** Treatment sessions were delivered by a psychologist with a focus on CBT approaches for weight control. One of the treatment sessions was delivered by a dietitian which focused on healthy eating for weight loss.	

*mean change is statistically significant as reported in the study
The US Academy of Nutrition and Dietetics' Quality Criteria Checklist: Primary Research was used to complete the quality assessments.
Abbreviations
Mo, months; wk, weeks, y, year; mins, minutes; IG-WN, Weight Neutral Intervention Group; CG, No treatment or waitlist control; IG, Weight Management Intervention Group; n, sample size; SD, standard deviation; R, Retention; B, Baseline; Post, Post-Treatment; FU, Follow Up; NR, not reported; F, female; P, personnel delivering intervention; PA, physical activity; BES, Binge Eating Scale; EDE-Q, Eating Disorder Examination Questionnaire; MAEDS, The Multifactorial Assessment of Eating Disorders Symptoms; EDI, Eating Disorder Inventory; EDE, Eating Disorder Examination; NESQ, Night Eating Syndrome Questionnaire; BITE, Bulimic Investigatory Test of Edinburgh; EDDS, Eating Disorder Diagnostic Screening; EAT, Eating Attitudes Test;

Table S3: Summary of outcome data for included studies reporting mean scores/ mean change in score

Study Author/year	Tool	IG/CG	Sample size (n) at baseline	Baseline Mean (SD)	During and post-intervention time points, mean (SD)	Follow-up timepoints (from end of intervention), mean (SD)	Mean change Base-post	Mean change Post-FU	Mean change Base-FU	Comments
Afari et al. 2019; Wooldridge et al. 2019[2,61]	BES	IG1	43	15.7 (9.2)	4-wk 13.6 (9.0)	3-mo 11.9 (8.6); 6-mo 11.9 (7.4)	4.24 (1.26, 7.22)*		3-mo 2.52 (-0.42, 5.45); 6 -mo 2.95 (-0.32, 6.22)	
		IG2	42	16.8 (8.5)	4-wk 10.6 (7.2)	3-mo 10.6 (7.4); 6-mo 10.0 (7.6)				
Ariel et al. 2016[3]	BES	IG1	136	15.1 (8.1)		4-mo 11.0 (7.4)	-4.1 (0.69)			IG2 and IG3 had greater reductions in BE than IG1 (low) and IG4 (control), p<0.01
		IG2	127	14.5 (8.2)		2-mo 7.9 (5.9)	-6.7 (0.67)			
		IG3	151	15.0 (8.0)	6-mo 8.7 (6.4)		-6.2 (0.57)			
		IG4	158	14.8 (8.9)		4-mo 11.5 (7.3)	-3.4 (0.60)			
Bacon et al. 2002; Bacon et al. 2005[4,5]	EDI Drive for thinness	IG	22	6.0 (5.0)	3-mo 3.5 (3.0)*; 6-mo 3.5 (2.8)*	6-mo 2.9 (2.9)*			With-in group P = 0.000	
		IG-WN	29	7.5 (5.1)	3-mo 5.2 (5.6)*; 6-mo 3.5 (4.0)*	6-mo 2.9 (3.9)			p = 0.006	
	EDI Bulimia	IG	22	5.0 (4.0)	3-mo 1.4 (1.7)*; 6-mo 1.2 (2.0)*	6-mo 1.0 (1.5)*			Within group P = 0.000;	
		IG-WN	29	4.4 (3.5)	3-mo 2.4 (2.5)*; 6-mo 1.3 (1.4)*	6-mo 0.9 (1.8)*			P-value = 0.000	
	EDI Body dissatisfaction	IG	22	19.4 (5.1)	3-mo 18.3 (7.4); 6-mo 15.5 (6.8)*	6-mo 17.2 (8.4)			Within group P= 0.087;	
		IG-WN	29	18.8 (4.2)	3-mo 17.7 (8.4); 6-mo 13.8 (7.2)*	6-mo 15.0 (8.2)*			P = 0.001	
	EDI Ineffectiveness	IG	22	4.4 (5.2)	3-mo 2.8 (5.1)*; 6-mo 1.9 (4.9)*	6-mo 2.3 (4.7)*			Within group P= 0.003;	
		IG-WN	29	3.3 (3.3)	3-mo 3.3 (3.5); 6-mo 2.3 (2.5)	6-mo 2.0 (3.5)*			P = 0.030	
	EDI Perfectionism	IG	22	6.8 (5.7)	3-mo 5.7 (6.0); 6-mo 6.6 (6.1)	6-mo 6.0 (5.8)			Between group P= 0.154;	
		IG-WN	29	6.0 (4.0)	3-mo 5.1 (3.9)*; 6-mo 5.0 (3.5)*	6-mo 5.1 (3.1)*			P = 0.042	
	EDI Interpersonal distrust	IG	22	2.5 (2.5)	3-mo 2.1 (3.0); 6-mo 1.4 (2.3)*	6-mo 1.5 (2.6)*			Within group P= 0.039;	
		IG-WN	29	2.0 (3.1)	3-mo 2.2 (2.7); 6-mo 1.9 (2.1)	6-mo 1.5 (2.4)			P = 0.218	
	EDI Interoceptive awareness	IG	22	4.2 (4.9)	3-mo 2.0 (2.9)*; 6-mo 1.5 (2.6)*	6-mo 1.3 (1.9)*			Within group P=0.000;	
		IG-WN	29	4.1 (3.3)	3-mo 4.5 (4.7); 6-mo 3.3 (3.8)*	6-mo 2.3 (3.9)*			p-value = 0.011	
		IG	22	1.1 (1.5)	3-mo 0.9 (1.3); 6-mo 0.4 (0.7)	6-mo 0.5 (1.0)			Within group P= 0.130	

Study Author/year	Tool	IG/CG	Sample size (n) at baseline	Baseline Mean (SD)	During and post-intervention time points, mean (SD)	Follow-up timepoints (from end of intervention), mean (SD)	Mean change Base-post	Mean change Post-FU	Mean change Base-FU	Comments
	EDI Maturity fears	IG-WN	29	1.2 (1.9)	3-mo 1.8 (3.2)*; 6-mo 1.3 (2.6)*	6-mo 1.4 (2.4)*			P-value = 0.582	
Barnes et al 2014; Barnes et al. 2017[6,62]	EDE (baseline, post & 3-mo FU)<br><br>EDE-Q (base & 12-mo follow-up)	IG1	30	EDE 2.1 (0.9) EDE-Q 2.39 (0.99)	3-mo 1.7 (0.8)	3-mo 1.7 (1.0)			12-mo FU mean change (SD) -0.343 (0.836)	No between group difference at 12-mo<br><br>Significant decreases in disordered eating symptoms (Barnes 2014, group not specified)
		IG2	29	EDE 1.6 (0.8) EDE-Q 2.12 (1.06)	3-mo 1.2 (0.5)	3-mo 1.3 (0.7)			-0.550 (0.727)	
		IG3	30	EDE 1.6 (0.9)	3-mo 1.5 (0.9)	3-mo 1.5 (0.9)				
Beaulieu et al. 2020[8]	BES	IG1	24	15 (9)	3-mo 9 (7)*		BE score decreased post-WL (P < 0.001)			
		IG2	22	16 (7)	3-mo 12 (5)*					
Bolognese et al. 2020[9]	EAT	IG1	37				no sig differences (p > 0.05)			
		IG2	37							
Carels et al. 2014[11]	BES	IG1	29	22 (10.8)	3-mo 16.6 (8.5)		sig reduced pre- to posttreatment, p < .001*, Cohen's d = 1.85.			Group time interaction NS
		IG2	30	23.1 (8.8)	3-mo 15 (7.2)					
Carels et al. 2019[12]	BES	IG1	19	36.6 (5.9)	4-mo 32.6 (6.7)		Within p<0.001 (seems like for all groups)			Between groups, P=0.09
		IG2	21	35.0 (7.3)	4-mo 32.0 (6.7)					
		IG3	26	32.3 (8.8)	4-mo 27.0 (6.3)					
Carpenter et al. 2019[13]	BES	IG1	42	19.2 (6.8)	6-mo 11.5 (8.1)					Between group difference p=0.006
		IG2	22	18.0 (7.5)	6-mo 15.9 (7.3)					
Cheng et al. 2014[14]	BES	IG1					12-mo -5.38 (1.49)			Mean change (SE) Outcomes not significant between groups at 12-mo p=0.71
		IG2					12-mo -3.93 (2.08)			
Christaki et al. 2013[15]	EAT-26	IG1	18	17.39 (9.61)			8-wk -0.6 (2.2)			Mean change (SE)

Study Author/year	Tool	IG/CG	Sample size (n) at baseline	Baseline Mean (SD)	During and post-intervention time points, mean (SD)	Follow-up timepoints (from end of intervention), mean (SD)	Mean change Base-post	Mean change Post-FU	Mean change Base-FU	Comments
		IG2	16	15.24 (8.2)			8-wk -0.6 (2.2)			
Dalle Grave et al. 2013[17]	BES	IG1	43	13.5 (9.8)	3-wk 7.5 (7.7); 27-wk 5.6 (5.6); 52-wk 6.9 (6.7)					No sig differences between groups were observed at any time point
		IG2	45	13.9 (9.6)	3-wk 8.1 (6.5); 27-wk 5.5 (6.6); 52-wk 5.8 (6.9)					
Dassen et al. 2018[18]	EDE-Q	IG1	51	2.56 (0.78)	42 days 2.24 (0.67)	6-mo 1.94 (0.91), 1.93 (0.95)				No difference between groups at any time point
		IG2	40	2.30 (0.86)	42 days 1.98 (0.62)	6-mo 1.73 (0.94), 1.61 (0.89)				
Dennis et al. 1999[20]	BES	IG1	21	18.0 (8.0)	16-wk 8.2 (1.5)*		Significant reduction post-treatment			Significant differences between groups at pre-treatment
		IG2	18	12.5 (7.7)	16-wk 11.5 (1.8)					
Dennis et al. 2001[19]	BES	Assured in IG1 (AT)	20	11.7 (7.6)	24-wk 10.4 (5.9)					
		Assured in IG3 (NT)	17	11.2 (5.5)	24-wk 7.5 (4.4)*					
		Disbeliever s in IG2 (DT)	10	21.8 (11.1)	24-wk 17.8 (8.3)					
		Disbeliever s in IG3 (NT)	12	17.8 (8.8)	24-wk 10.9 (8.2)*					
Di Marco et al. 2009[21]	EDE-Q Eating Concern	IG1	20	1.02 (0.67)	3-mo 0.71 (0.56)*		Sig within group decrease, =0.02			Between group difference for EC, but no other sub-scales
		IG2	19	1.18 (1.18)	3-mo 1.35 (1.40)					
	EDE-Q restraint	IG1	20	1.52 (1.30)	3-mo 2.56 (1.20)					
		IG2	19	1.55 (1.01)	3-mo 2.13 (1.17)					
	EDE-Q shape concern	IG1	20	3.79 (1.30)	3-mo 2.58 (1.36)					
		IG2	19	3.31 (1.11)	3-mo 2.91 (1.36)					
	EDE-Q	IG1	20	2.80 (0.97)	3-mo 2.34 (0.74)					

Study Author/year	Tool	IG/CG	Sample size (n) at baseline	Baseline Mean (SD)	During and post-intervention time points, mean (SD)	Follow-up timepoints (from end of intervention), mean (SD)	Mean change Base-post	Mean change Post-FU	Mean change Base-FU	Comments
	Weight concern	IG2	19	2.84 (0.97)	3-mo 2.80 (1.19)					
Fogelholm et al. 1999[22]	BITE	IG1	During WR, n = 78<br><br>During WM, n = 72	<20 (ppl with score >20 excluded)			During WR phase, 12w -2.5 (0.7); WM phase, 52w -0.3 (0.4)			Mean (SEM)<br>BE decreased during WR phase in all groups; no change during WM phase, no diff b/w groups<br><br>N per group NR
		IG2		<20			-1.2 (0.6); -1.1 (0.5)			
		IG3		<20			-3.4 (0.9); -0.2 (0.7)			
Glynn et al. 2022[23]	BES	IG1	103	10.7 (0.5)	28 days 8.3 (0.5)*; 56 days 7.7 (0.5)*; 84 days 7.3 (0.5)*		From baseline to post, p<0.05			Data are mean (SE)
		IG2	103	11.1 (0.5)	28 days 8.2 (0.5)*; 56 days 7.5 (0.5)*; 84 days 7.6 (0.5)*		From baseline to post, p<0.05			
Goodrick et al.1998[24]	BES	IG	65	27.82 (6.13)	6-mo 15.42 (7.42)	12-mo 14.25 (8.93)	-12.40 (SD NR)<br><br>No diff b/w IG and IG-WN, p=0.27		-13.57 No diff b/w IG and IG-WN, p=0.66	
		IG-WN	62	27.58 (5.13)	6-mo 17.29 (7.77)	12-mo 14.90 (10.40)	-10.29		-12.68	
		CG	58	27.88 (5.28)	6-mo 24.22 (8.85)	NR	-3.66		NR	Sig diff between CG and IG/IG-WN p<0.002
Jeffery et al. 1998[25]	BES	IG1	41	15.7 (1.3)						No treatment group differences. Data NR.
		IG2	42	18.3 (1.3)						
		IG3	37	17.7 (1.3)						
		IG4	36	14.2 (1.3)						
		IG5	40	16 (1.3)						
Jospe et al. 2017[26,63]	EDE-Q	IG1	38	2.19 (0.90)	12-mo 2.17 (0.92)		12-mo 0.13 (-0.24 to 0.50)			Mean difference reported relative to IG5 (active control), no sig difference between any groups relative to IG5
		IG2	36	2.15 (0.90)	12-mo 2.04 (1.03)		12-mo 0.02 (-0.36 to 0.39)			
		IG3	32	1.70 (0.83)	12-mo 1.62 (0.86)		12-mo -0.10 (-0.46 to 0.27)			
		IG4	28	1.89 (0.99)	12-mo 1.84 (1.02)		12-mo -0.01 -0.37 to 0.35)			
		IG5	35	1.97 (0.92)	12-mo 1.88 (1.02)		N/A			
Keranen et al. 2009[28]	BES	IG1	35				18mo (delta values) -7 (CI 95% -10;-4)			

Study Author/year	Tool	IG/CG	Sample size (n) at baseline	Baseline Mean (SD)	During and post-intervention time points, mean (SD)	Follow-up timepoints (from end of intervention), mean (SD)	Mean change Base-post	Mean change Post-FU	Mean change Base-FU	Comments
		IG2	47				18mo -5 (CI 95% -7;-2)			Significant reduction in BE in both groups p<0.01, no difference between groups
LaRose et al. 2014[30]	EDDS	Daily weighing	29	Mean (SE) 18.82 (1.6)	6-mo 11.77 (0.57); 12-mo 11.86 (0.68)* , 18mo 11.54 (0.72)*		6-mo -4.5 (SE=0.7); 12-mo -3.4 (SE=0.8); 18mo -4.0 (SE=0.8)			No diff between groups at 6 and 18 months. At 12-mo participants reporting weighing <daily had a higher score p=0.03
		<daily weighing	148	16.08 (0.70)	6-mo 13.91 (1.3); 12-mo 15.35 (1.1)*; 18mo 14.14 (1.1)*					
Martin et al. 2019[33]	MAEDS – Binge Eating	IG1	59	54.8 (52.0, 57.6)			24-wk -1.97 (-3.58, -0.36)			Least squares mean (95% CI)<br><br>*IG3 significantly differed from IG1 and IG2 (p=0.01)
		IG2	51	55.7 (52.7, 58.7)			24-wk -3.42 (-5.12, 1.71)			
		IG3	61	56.4 (53.6, 59.1)			24-wk -3.07 (-4.63, -1.50)			
	MAEDS-Purgative Behaviour	IG1	59	46.8 (45.7, 48.0)			24-wk 0.27 (-0.74, 1.27)			
		IG2	51	46.6 (45.3, 47.8)			24-wk -0.49 (-1.55, 0.57)			
		IG3	61	47.7 (46.6, 48.8)			24-wk -0.06 (-1.03, 0.92)			
	MAEDS – Restrictive Eating	IG1	59	47.1 (45.4, 48.7)			24-wk 0.11 (-1.16, 1.37)			
		IG2	51	46.7 (44.9, 48.5)			24-wk -1.92 (-3.25, -0.59)			
		IG3	61	50.1 (48.5, 51.8)*			24-wk -1.09 (-2.32, 0.13)			
Mason et al. 2019[32]	BES	IG1	117	4.59 (2.97)	12-mo 3.50 (2.56)		Change (95%CI) -1.09 (-1.55, -0.63)			Significant reduction compared to CG p=0.005
		IG2	114	3.51 (2.84)	12-mo 3.49 (2.66)		-0.02 (-0.46, 0.43)			
		IG3	115	3.91 (2.58)	12-mo 3.28 (2.42)		-0.63 (-1.03, -0.24)			
		CG	87	4.36 (3.00)	12-mo 4.25 (3.30)		-0.11 (-0.59, 0.38)			
Mensinger et al. 2016[35]	EDE-Q Global scores	IG-WN	40	2.58 (0.11)	6-mo 1.75 (0.11)* (to baseline)	24-mo 2.00 (0.15)* (to baseline)				Between group difference 6-mo: 0.66 (0.27 to 1.05) 24-mo: 0.32 (-0.16 to 0.77)
		IG	40	2.35 (0.11)	6-mo 2.19 (0.11)	24-mo 2.10 (0.14)				

Study Author/year	Tool	IG/CG	Sample size (n) at baseline	Baseline Mean (SD)	During and post-intervention time points, mean (SD)	Follow-up timepoints (from end of intervention), mean (SD)	Mean change Base-post	Mean change Post-FU	Mean change Base-FU	Comments
	Weight concern	IG-WN	40	3.35 (0.13)	6-mo 2.35 (0.12)*	24-mo 2.18 (0.17)*				Between group difference 6-mo 0.24 (-0.21 to 0.70) 24-mo 0.27 (-0.28 to 0.81)
		IG	40	3.24 (0.13)	6-mo 2.52 (0.13)*	24-mo 2.38 (0.16)*				
	Shape concern	IG-WN	40	4.29 (0.16)	6-mo 2.88 (0.15)*	24-mo 2.97 (0.21)*				Between group difference 6-mo 0.42 (-0.13 to 0.96) 24-mo 0.46 (-0.19 to 1.11)
		IG	40	4.13 (0.16)	6-mo 3.15 (0.16)*	24-mo 3.29 (0.20)*				
	Eating concern	IG-WN	40	1.53 (0.15)	6-mo 0.83 (0.14)*	24-mo 0.60 (0.19)*				Between group difference 6-mo 0.46 (0.01 to 0.91) 24-mo 0.37 (-0.17 to 0.92)
		IG	40	1.13 (0.15)	6-mo 0.88 (0.19)	24-mo 0.57 (0.19)*				
	Dietary restraint	IG-WN	40	1.15 (0.18)	6-mo 0.91 (0.17)	24-mo 1.40 (0.25)				Between group difference 6-mo 1.54 (0.89 to 2.20) 24-mo 1.02 (0.23 to 1.81)
		IG	40	0.89 (0.18)	6-mo 2.22 (0.18)*	24-mo 2.16 (0.23)*				
Moss et al. 2017[36]	EDE-Q Global Scores	IG1	69	2.27 (0.14)	12-wk 2.01 (0.08)*	1-mo 1.91 (0.08)*; 6-mo 1.96 (0.08)*	Sig reduction, p=0.001		Sig reduction, p<0.001	Between group difference 12-wk: -0.002 1-mo: 0.214 6-mo: -0.135<br><br>No sig difference between groups
		IG2	66	2.34 (0.11)	12-wk 2.07 (0.08)*	1-mo 2.13 (0.09)*; 6-mo 1.92 (0.08)*	Sig reduction, p=0.001		Sig reduction, p<0.001	
	Dietary Restraint	IG1	69	1.88 (0.16)	12-wk 2.39 (0.13)*	1-mo 2.01 (0.14); 6-mo<br>1.58 (0.13)*	Sig increase, p<0.001		No change b/w/ base and 1-mo FU; sig reduction b/w base and 6-mo FU, p=0.007	Between group difference 12-wk: 0.096 1-mo:-0.185 6-mo: -0.005<br><br>No sig difference between groups For all subscales
		IG2	66	2.10 (0.15)	12-wk 2.47 (0.14)*	1-mo 2.35 (0.14); 6-mo 1.75 (0.14)*	Sig increase, p<0.001			
	Eating Concern	IG1	69	1.12 (0.17)	12-wk 0.89 (0.9)	1-mo 1.0 (0.09); 6-mo 1.09 (0.09)	No sig change		No sig change	Between group difference 12-wk: 0.018 1-mo: 0.098 6-mo: -0.109
		IG2	66	0.99 (0.13)	12-wk 0.82 (0.10)	1-mo 1.03 (0.10); 6-mo 0.94 (0.10)	No sig change		No sig change	
	Weight Concern	IG1	69	2.89 (0.15)	12-wk 2.28 (0.11)*	1-mo 2.24 (0.11)*; 6-mo 2.37 (0.10)*	Sig reduction, p<0.001		Sig reduction, p<0.001	Between group difference 12-wk: 0.089 1-mo: 0.116 6-mo: -0.081
		IG2	66	2.92 (0.14)	12-wk 2.46 (0.11)*	1-mo 2.44 (0.11)*; 6-mo 2.39 (0.11)*	Sig reduction, p<0.001		Sig reduction, p<0.001	
	Shape Concern	IG1	69	3.25 (0.2)	12-wk 2.54 (0.13)*	1-mo 2.43 (0.13)*; 6-mo 2.81 (0.12)*	Sig reduction, p<0.001		Sig reduction, p<0.001	Between group difference 12-wk: -0.097

Study Author/year	Tool	IG/CG	Sample size (n) at baseline	Baseline Mean (SD)	During and post-intervention time points, mean (SD)	Follow-up timepoints (from end of intervention), mean (SD)	Mean change Base-post	Mean change Post-FU	Mean change Base-FU	Comments
		IG2	66	3.39 (0.19)	12-wk 2.61 (0.13)*	1-mo 2.72 (0.13)*; 6-mo 2.72 (0.13)*	Sig reduction, p<0.001		Sig reduction, p<0.001	1-mo: 0.194<br>6-mo: -0.144
Muggia et al. 2014[37]	BITE	IG1	83	4.28 (2.56)	6-mo 4.04 (2.56)	12-mo 3.20 (2.17)				
		IG2	80	4.52 (2.94)	6-mo 3.33 (2.59)	12-mo 2.97 (2.47)				
Radin et al. 2020[40]	BES	IG1	100	15.49 (6.83)						Reduction in both groups. Greater decrease in IG1 (mindful arm) compared to IG2 at 12-mo, p=0.008
		IG2	94	15.79 (7.47)						
Ramirez et al. 2001[43]	EDE-Q-Eating concern	IG1	38	10.3 (5.96)	16-wk 3.63 (3.04)	3-mo 4.21 (3.86); 12-mo 4.61 (4.42)*				Sig reduction in both groups in eating concern p<0.001
		IG2	27	8.74 (6.51)	16-wk 4.92 (5.32)	3-mo 5.54 (5.00); 12-mo 7.09 (6.97)*				
	EDE-Q Restraint	IG1	38	8.89 (5.69)	16-wk 13.00 (4.95)	3-mo 8.84 (5.79); 12-mo 8.31 (4.80)				
		IG2	27	6.67 (6.47)	16-wk 13.90 (2.92)	3-mo 10.5 (4.93); 12-mo 8.13 (5.06)				
Rapoport et al. 2000[44]	BES	IG1	37	14 (9)	10-wk 8 (7)	24-wk 10 (9); 12-mo 9 (8)				Significant change over time p<0.001
		IG2	38	15 (9)	10-wk 6 (5)	24-wk 8 (9); 12-mo 9 (8)				
Raynor et al. 2006[45]	BES	IG1 and IG2 combined	IG1 n=15, IG2 n=15	16.3 (7.7)	9-wk 11.6 (7.0)*		No difference between groups			
Rieger et al. 2017[46,47]	BES	IG1	98	18.10 (8.11)	12-mo 11.41 (7.34)	24-mo 12.11 (7.63)				Sig decrease from baseline in both groups, no diff between groups
		IG2	98	17.01 (7.67)	12-mo 10.37 (6.28)	24-mo 11.71 (7.19)				
Smith et al. 2018[51]	BES	IG1	18	16.94 (8.25)	6-wk 8.37 (4.59)	4-mo 9.27 (4.99); 9-mo 10.57 (7.23)	-7.171 SE (1.264)			Both groups showed significant reductions on the BES from base to post
		IG2	18	12.66 (7.42)	6-wk 8.76 (5.94)	4-mo 7.47 (5.34); 9-mo 7.27 (5.81)	-4.293 SE (0.866)			
Steinberg et al 2014[52]	Anorectic Cognition Scale (ACS)	IG	47	32.9 (1.0)	3-mo 32.5 (1.0); 6-mo 31.7 (1.0)					Mean (SE)<br><br>No difference between groups
		CG	44	31.9 (1.0)	3-mo 32.6 (1.1); 6-mo 31.50 (1.0)					

Study Author/year	Tool	IG/CG	Sample size (n) at baseline	Baseline Mean (SD)	During and post-intervention time points, mean (SD)	Follow-up timepoints (from end of intervention), mean (SD)	Mean change Base-post	Mean change Post-FU	Mean change Base-FU	Comments
Tanco et al. 1998[53]	EDI Drive for thinness	IG-WN	18	7.9 ± 6.2	4-wk 6.9 ± 6.0; 8-wk 3.8 ± 4.4*	6-mo, n=12; 4.4 ± 3.9				Scores for IG-WN improved over time in some subscales. No change in IG and CG
		IG	19	6.1 ± 3.9	4-wk 6.5 ± 5.1; 8-wk 5.3 ± 4.6	6-mo, n=9; 7.1 ± 5.2				No change at FU in those attending the 6-mo assessment
		CG	13	7.2 ± 4.7	4-wk 6.0 ± 4.5; 8-wk 6.5 ± 4.6	NR				
	EDI Bulimia	IG-WN		5.9 ± 5.4	4-wk 2.2 ± 2.7; 8-wk 1.3 ± 1.8*	6-mo 1.4 ± 1.5				
		IG		5.0 ± 3.7	4-wk 3.5 ± 3.4; 8-wk 3.5 ± 4.1	6-mo 4.0 ± 4.3				
		CG		5.0 ± 4.5	4-wk 4.5 ± 5.8; 8-wk 4.1 ± 5.0	NR				
	EDI Body dissatisfaction	IG-WN		22.2 ± 5.4	4-wk 20.3 ± 7.3; 8-wk 16.5 ± 8.9*	6-mo 18.6 ± 7.0				
		IG		18.6 ± 6.7	4-wk 18.4 ± 7.1; 8-wk 18.1 ± 7.8	6-mo 18.4 ± 6.0				
		CG		21.2 ± 6.3	4-wk 19.4 ± 6.9; 8-wk 20.3 ± 6.0	NR				
	EDI Inefficiency	IG-WN		7.9 ± 6.4	4-wk 5.7 ± 4.3; 8-wk 3.9 ± 5.1*	6-mo 3.4 ± 4.1				
		IG		5.2 ± 4.2	4-wk 3.6 ± 4.3; 8-wk 4.2 ± 4.6	6-mo 5.9 ± 6.2				
		CG		7.9 ± 5.1	4-wk 6.5 ± 5.8; 8-wk 6.0 ± 5.6	NR				
	EDI Perfectionism	IG-WN		6.8 ± 4.1	4-wk 6.3 ± 4.1;8-wk 6.3 ± 3.8	6-mo 8.0 ± 4.2				
		IG		6.0 ± 3.8	4-wk 5.3 ± 4.2; 8-wk 5.2 ± 4.7	6-mo 4.8 ± 5.0				
		CG		5.2 ± 4.5	4-wk 4.5 ± 4.1; 8-wk 4.8 ± 4.5	NR				
	EDI Interpersonal distrust	IG-WN		3.4 ± 3.9	4-wk 3.3 ± 3.8; 8-wk 3.1 ± 4.4	6-mo 3.6 ± 4.7				
		IG		3.6 ± 3.3	4-wk 1.6 ± 2.0; 8-wk 2.1 ± 2.7	6-mo 2.2 ± 2.8				
		CG		2.9 ± 2.9	4-wk 3.4 ± 3.3; 8-wk 2.5 ± 3.3	NR				
	EDI Interoceptive awareness	IG-WN		7.6 ± 6.9	4-wk 4.6 ± 4.9*; 8-wk 3.6 ± 3.7*	6-mo 2.8 ± 2.6				
		IG		4.8 ± 4.3	4-wk 3.3 ± 3.4; 8-wk	6-mo 1.8 ± 3.5				

Study Author/year	Tool	IG/CG	Sample size (n) at baseline	Baseline Mean (SD)	During and post-intervention time points, mean (SD)	Follow-up timepoints (from end of intervention), mean (SD)	Mean change Base-post	Mean change Post-FU	Mean change Base-FU	Comments
					4.4 ± 4.5					
		CG		5.6 ± 3.8	4-wk 5.4 ± 5.3; 8-wk 4.2 ± 4.8	NR				
	EDI Maturity fears	IG-WN		2.3 ± 2.3	4-wk 1.8 ± 1.9; 8-wk 1.9 ± 2.0	6-mo 1.3 ± 1.8				
		IG		2.1 ± 2.6	4-wk 1.6 ± 1.8; 8-wk 1.9 ± 1.8	6-mo 1.0 ± 1.8				
		CG		2.9 ± 3.1	4-wk 3.0 ± 2.9; 8-wk 2.2 ± 2.8	NR				
Vander Wal et al. 2006[54]	NESQ	IG1	29	17.28 (7.81)			0.68 (6.36)			No change in either group
		IG2	32	19.78 (7.82)			-2.72 (7.59)			
Wadden et al. 1994[55]	BES	IG1	17	22.88 (8.18)	26-wk 13.71 (7.60)*; 52-wk 12.00 (6.78)*		Sig reduced 26 and 52 wk for both groups p<0.001			Greater reduction in IG1 than IG2 at 52wk p<0.02
		IG2	23	23.46 (7.27)	26-wk 17.08 (8.87)*; 52-wk18.32 (8.18)*					
Werrij et al. 2009[57]	EDE-Q Restraint	IG1	96	1.46 (1.07)	3.5-mo 2.06 (1.08); 12-mo 2.00 (1.03)					
		IG2	104	1.27 (0.97)	3.5-mo 1.97 (1.04); 12-mo NR					
	EDE-Q Eating concerns	IG1	96	1.33 (1.16)	3.5-mo 1.18 (1.03); 12-mo 1.09 (1.04)					Both groups reduced global score, shape, weight and eating concern. No diff between groups. IG2 showed a partial relapse in eating and weight concern between post-FU
		IG2	104	1.19 (1.17)	3.5-mo 0.86 (0.99); 12-mo NR					
	EDE-Q Weight concerns	IG1	96	3.09 (1.15)	3.5-mo 2.46 (1.24); 12-mo 2.41 (1.33)					
		IG2	104	2.54 (1.19)	3.5-mo 1.97 (1.23); 12-mo NR					
	EDE-Q Shape concerns	IG1	96	3.58 (1.46)	3.5-mo 2.75 (1.54); 12-mo 2.77 (1.62)					
		IG2	104	3.05 (1.52)	3.5-mo 2.26 (1.53); 12-mo NR					
	EDE-Q Global score	IG1	96	2.36 (0.94)	3.5-mo 2.11 (0.94); 12-mo 2.07 (1.04)					
		IG2	104	2.04 (0.99)	3.5-mo 1.77 (0.91); 12-mo NR					
	BES	IG1	53	16.6 (7.6)			8-wk -1.3 (5.7)			

Study Author/year	Tool	IG/CG	Sample size (n) at baseline	Baseline Mean (SD)	During and post-intervention time points, mean (SD)	Follow-up timepoints (from end of intervention), mean (SD)	Mean change Base-post	Mean change Post-FU	Mean change Base-FU	Comments
Whitelock et al. 2019[58]		IG2	54	16.5 (7.5)			8-wk -2.3 (5.8)			No difference between groups
Williamson et al. 2008[59]	MAEDS – Binge eating	IG1	12	52.4 (3.1)			6-mo change from baseline -6.5 (1.9); 9-mo change from baseline - 7.5 (1.9); 12-mo change from baseline -8.2 (2.0)			Data are mean (SE) and mean change (SE)<br>No diff b/w groups<br><br>All baseline data = mean (SE). During, post-intervention and follow up = least square mean (SE)<br><br>During the 6 month follow up period, no differences between the minimal and extended care groups were found for any variable
		IG2	12	49.5 (3.1)			6-mo change from baseline -7.6 (2.3); 9-mo change from baseline -5.0 (2.3); 12-mo change from baseline -5.0 (2.6)			
		IG3	12	44.4 (2.1)			6-mo change from baseline -6.3 (2.0); 9-mo change from baseline -7.0 (2.0); 12-mo change from baseline -7.1 (2.0)			
		IG4	12	45.3 (3.2)			NR			
	MAEDS – Purgative behavior	IG1	12	45.8 (1.1)			6-mo change from baseline -1.4 (0.7); 9-mo change from baseline 0.1 (0.7); 12-mo change from baseline -0.8 (0.7)			At 6 months, no significant treatment arm effects were detected
		IG2	12	47.6 (2.3)			6-mo change from baseline -0.2 (0.8); 9-mo change from baseline 1.3 (0.8); 12-mo change from baseline 0.4 (1.0)			
		IG3	12	43.8 (0.5)			6-mo change from baseline -0.6 (0.7); 9-mo change from baseline 1.1 (0.7); 12-mo change from baseline -0.1 (0.7)			
		IG4	12	44.8 (1.1)			NR			

Study Author/year	Tool	IG/CG	Sample size (n) at baseline	Baseline Mean (SD)	During and post-intervention time points, mean (SD)	Follow-up timepoints (from end of intervention), mean (SD)	Mean change Base-post	Mean change Post-FU	Mean change Base-FU	Comments
	MAEDS – Restrictive eating	IG1	12	43.3 (1.8)			6-mo change from baseline -2.9 (1.8); 9-mo change from baseline 0.1 (1.8); 12-mo change from baseline -0.8 (1.8)			At 6 months, restrictive eating did not differ across treatment arms
		IG2	12	46.0 (2.0)			6-mo change from baseline -3.8 (2.1); 9-mo change from baseline -0.8 (2.1); 12-mo change from baseline 0.7 (2.3)			
		IG3	12	46.1 (1.3)			6-mo change from baseline -3.3 (1.8); 9-mo change from baseline -1.6 (1.8); 12-mo change from baseline -2.0 (1.8)			
		IG4	12	42.6 (1.6)			NR			
Zwickert et al. 2016[60]	BES	IG1	31	18.9 (1.4)	3-mo 8.8 (1.5); 6-mo 9.5 (1.7); 9-mo 9.7 (1.8); 15mo 13.4 (1.9)		Both groups show significant reductions in BES, maintained at 15mo P<0.01)			Mean (SE)<br><br>No significant increase in BES scores from 9 to 15 months, no difference between groups
		IG2	29	18.1 (1.5)	3-mo 11.4 (1.5); 6-mo 12.3 (1.6); 9-mo 13.0 (1.8); 15mo 13.1 (2.0)					

*mean change is statistically significant as reported in the study

Abbreviations

Mo, months; wk, weeks, y, year; mins, minutes; IG-WN, Weight Neutral Intervention Group; CG, No treatment or waitlist control; IG, Weight Management Intervention Group; n, sample size; SD, standard deviation; R, Retention; B, Baseline; Post, Post-Treatment; FU, Follow Up; NR, not reported; F, female; P, personnel delivering intervention; PA, physical activity; BES, Binge Eating Scale; EDE-Q, Eating Disorder Examination Questionnaire; MAEDS, The Multifactorial Assessment of Eating Disorders Symptoms; EDI, Eating Disorder Inventory; EDE, Eating Disorder Examination; NESQ, Night Eating Syndrome Questionnaire; BITE, Bulimic Investigatory Test of Edinburgh; EDDS, Eating Disorder Diagnostic Screening; EAT, Eating Attitudes Test;

Table S4: Outcome data for studies reporting prevalence or frequency data for a behavior e.g. for binge eating/ eating disorder diagnosis

Study Author/year<br><br>IG/CG	Tool; measure	IG/CG	Baseline	During and post-intervention time points	Follow-up timepoints (from post-intervention)	Comments/ notes
Ariel et al. 2016[3]	BES<br>N (%) reporting moderate to severe BE	IG1	47 (34.6)		4-mo 22.6 (16.6)*	A sig lower percentage of participants in IG4 (82.0%) and IG1 (83.4%) reported Mild/No BE at 6-mo than participants in IG2 (95.6%) or IG3 (90.5%)
		IG2	45 (35.4)		2-mo 5.6 (4.4)*	
		IG3	53 (35.1)	6-mo 14.3 (9.5)*		
		IG4	54 (34.3)		4-mo 28.4 (18.0)	
Cooper et al. 2010[16]	EDE-interview<br>Presence and frequency of binge eating	Groups combined; no diff b/w groups	Any binge n=36, 24%	44-wk, n=24, 16%	3-y, n=25, 16.7% (n=7 belonged to the BE subgroup at baseline)	
			BE sub-group (≥12 episodes in 12 wks), n=14, 9.3%	44-wk, n=6 ceased binge eating	n=7 reported no BE (n=7 moved to 'any binge' group above)	
			Met BED criteria, n=6 4%	NR	NR	
			No BE, n=114, 76%	n=9 reported some binge eating (7.9%)		
LaRose et al. 2014[30]	EDDS<br># participants who met criteria for BED	Groups combined	n=36<br><br>(n=178 recruited, n=142 no BED at baseline)	6-mo, n=9; 12-mo, n=6; 18mo, n=7		Of those who met criteria for BED at baseline, all but 2 participants no longer met criteria for BED at 18 months. However, 9 participants who did not meet criteria for BED at baseline met criteria at one of the follow-up assessments. No participant met criteria for BED at all timepoints.
	Fasting/ skipping >2 meals		14%	no new cases		No sig effect of change over time for: 1) vomiting (p=.26); 2) use of laxatives or diuretics (p=.33); 3) fasting / skipping ≥ 2 meals in a row (p=.34); or 4) excessive exercise (p=.76).<br>No sig changes from baseline to any of the follow-up time points,
	Excessive exercise		18.5%	no new cases		
	Compensatory behaviors		No participants endorsed vomiting or use of laxatives or diuretics	6-mo, 3 participants who reported compensatory behaviors (1 vomiting and 3 laxative / diuretic use across 3 participants).<br><br>12-mo, 2 participants who reported compensatory behaviors (1 vomiting and 2 laxative/diuretics use)		

Study Author/year<br><br>IG/CG	Tool; measure	IG/CG	Baseline	During and post-intervention time points	Follow-up timepoints (from post-intervention)	Comments/ notes
				18mo, 3 participants who endorsed compensatory behaviors (1 vomiting and 2 laxative / diuretics use).		
Kalarachin et al. 2013[27]	EDE - OBE Episode in past 28 days	IG1	7.9 (20.2)	6-mo, 0.7(3.0)		Both groups improved over time p<0.001
		IG2	7.7(18.2)	6-mo, 1.3(4.2)		
	EDE - SBE Episode in past 28 days	IG1	4.8 (19.7)	6-mo, 0.7 (2.9)		Both groups improved over time p=0.02
		IG2	3.1 (12.2)	6-mo, 1.0 (3.8)		
Pacanowski et al. 2014[38,39]	EDDS<br>Binge eating, at least 1 episode/wk for 6-mo (n, %)	All groups combined	76, 19.4%	12-mo, 68, 19.8%; 24-mo, 54, 15.9%		30.1% at any timepoint
	No BE (n, %)		305, 80.6 %	12-mo, 275, 80.2%; 24-mo, 285, 84.1%		69.9% at any timepoint
	Severity (as a n, % of people reporting BE)<br><br>Mild, 1-3/wk		57, 75.0%	12-mo, 56, 82.4%; 24-mo, 44, 81.5%		As a proportion of people who reported BE at that timepoint, most people reported mild BE
	Moderate (n, %)		16, 21.1%	12-mo, 10, 14.7%; 24-mo, 8, 14.8%		
	Severe (n, %)		3, 3.9%	12-mo, 1, 1.5%; 24-mo, 1, 1.9%		
	Extreme (n, %)		0, 0%	12-mo, 1, 1.5%; 24-mo, 1, 1.9%		
Raman et al. 2018[42]	EDE-Q + clinical interview<br># binge episodes/ week	IG1	9.3 (8.7)	7-9-wk 3.2 (5.7)	3-mo 3.4 (6.0)	Significant difference between groups, p<0.01
		IG2	9.3 (10.6)	7-9-wk 11.6 (11.9)	3-mo 9.2 (10.6)	
Reiger et al. 2017[46]	BES<br>No BE (%)	IG1	50%	12-mo, 84.5%	12-mo, 80.9%	Significant decrease in the severity of binge eating categorisation across the trial but no difference between groups at any timepoint
		IG2	53.1%	12-mo, 83.3%	12-mo, 78%	
	Moderate BE	IG1	35.7%	12-mo, 10.3%	12-mo, 10.6%	
		IG2	35.7%	12-mo, 16.7%	12-mo, 17.1%	
	Severe BE	IG1	14.3%	12-mo, 5.2%	12-mo, 8.5%	
		IG2	11.2%	12-mo, 0%	12-mo, 4.9%	
Schyns et al. 2020[48]	EDE-Q + clinical interview<br><br>Binge eating frequency in last 7 days	Groups combined	NR	1-mo, marginally greater reduction in IG1 than IG2 p=0.056, both reduced	2mo, sig greater reduction in IG1 than IG2 p=0.03, both reduced	
	Vomiting		n=1		n=1	
	Excessive exercise, 28.9% At baseline	IG1	Mean (SD) IG1 1.13 (2.75); IG2 3.41 (6.16)		IG1 0.67 (2.11)	No significant change
		IG2			IG2 1.88 (3.14)	

Study Author/year<br><br>IG/CG	Tool; measure	IG/CG	Baseline	During and post-intervention time points	Follow-up timepoints (from post-intervention)	Comments/ notes
Simpson et al. 2015[50]	EDE-Q<br># days bingeing in last 28 days	IG1	2.2 (4.92)		12-mo 1.4 (2.61)	
		IG2	2.7 (4.02)		12-mo, 4.1 (6.06)	
		IG3	2.4 (4.04)		12-mo, 2.5 (4.14)	
	EDE-Q<br>Recurrent binge eating behavior y/n (%)	Groups combined	No, n=123		No, n=116, 94%; Yes, n=7, 6%	
			Yes, n=12		No, n=6; Yes, n=6, 50%	
	EDE-Q<br>Recurrent compensatory behavior y/n (%)	Groups combined	No, n=129		No, n=126, 98%; Yes, n=3, 2%	
			Yes, n=5		No, n=3, 60%; Yes, n=2, 40%	
Steinberg et al. 2014[52]	QEWP-R<br>Participants binge eating, n(%)	IG	14 (30%)	3-mo, 11 (27); 6-mo, 6 (14)		Sig decrease in binge eating in IG group. No change in CG. No diff between groups
		CG	8 (18%)	3-mo, 13 (32); 6-mo, 9 (21)		
Wadden et al. 1994[55]	BES<br>Severe binge eaters (score ≥27)	IG1	n=5	26wk, n=1; 52wk, n=1		
		IG2	n=9	26w, n=5; 52w, n=5		
Wadden et al. 2004[56]	EDE- OBE<br># days in last 28 days	IG1		9wk, 0 days; 20wk, 0.1 (0.2); 28wk, 0.0 (0.0) ; 40wk, 0.0 (0.0)		One episode of OBE in 2 participants at wk20
		IG2		9wk, 0 days; 20wk, 0.0 (0.0); 28wk, 0.2 (0.5); 40wk, 0.1 (0.7)		4 participants reported one OBE episode at wk 28, remitted by wk40<br><br>1 person had two episodes at 28wk and 4 episodes (in last 28 days) at 40wk
		IG-WN		9wk, 0 days; 20wk, 0.0 (0.0); 28wk, 0.0 (0.0); 40wk, 0.0 (0.0)		
	EDE- SBE<br># days in last 28 days	All groups combined	1.3 (4.2) days<br><br>67-77% reported no episodes of SBE	9wk, 0.2 (0.6); 20wk, returned to baseline		No differences between groups<br><br>Wk 40: n=7 in IG1 (though still below baseline) and n=1 in IG-WN reported SBE, p<0.03
Werrij et al. 2009[57]	Composed by authors (alongside EDE-Q)<br># binge episodes last 28 days	IG1	2.1 (7.33)	10wk, 0.56 (1.85); 14wk, 0.55 (1.95)		
		IG2	1.63 (5.07)	10wk, 0.77 (3.15); 14wk, NR		
Zwickert et al. 2016[60]	BES<br>Binge eating severity					25 participants (47%) went from 'moderate binge eating' to 'non-binge eating' category

*mean change is statistically significant as reported in the study

Abbreviations

Mo, months; wk, weeks, y, year; mins, minutes; IG-WN, Weight Neutral Intervention Group; CG, No treatment or waitlist control; IG, Weight Management Intervention Group; n, sample size; SD, standard deviation; R, Retention; B, Baseline; Post, Post-Treatment; FU, Follow Up; NR, not reported; F, female; P, personnel delivering intervention; PA, physical activity; BES, Binge Eating Scale; EDE-Q, Eating Disorder Examination Questionnaire; MAEDS, The Multifactorial Assessment of Eating Disorders Symptoms; EDI, Eating Disorder Inventory; EDE, Eating Disorder Examination; NESQ, Night Eating Syndrome Questionnaire; BITE, Bulimic Investigatory Test of Edinburgh; EDDS, Eating Disorder Diagnostic Screening; EAT, Eating Attitudes Test;

FIGURES

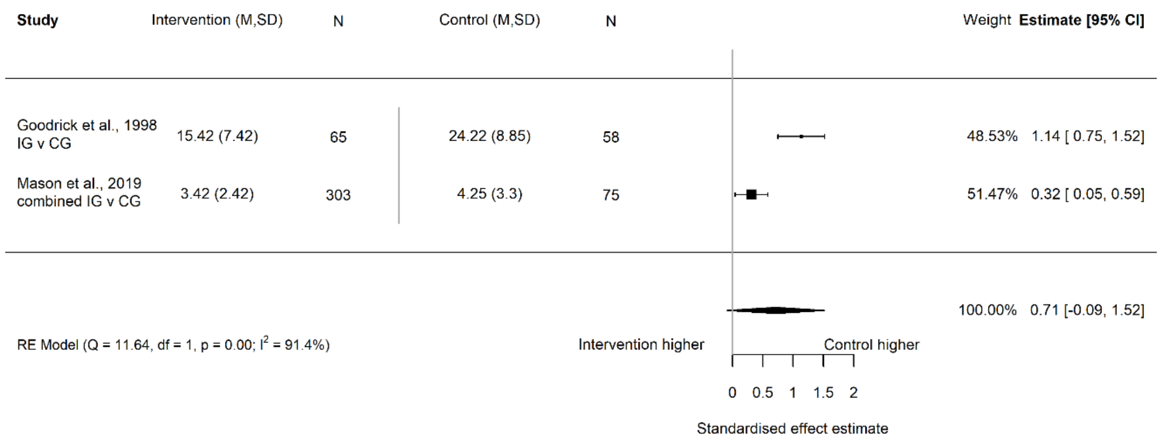

Figure S1 Forest plot of the difference in binge eating between the control group and intervention at post.
Each estimate was standardized using Hedges' g. Mason et al., 2019 had three intervention groups which were combined and compared against the control. A random effects model was used to combine estimates from each trial.

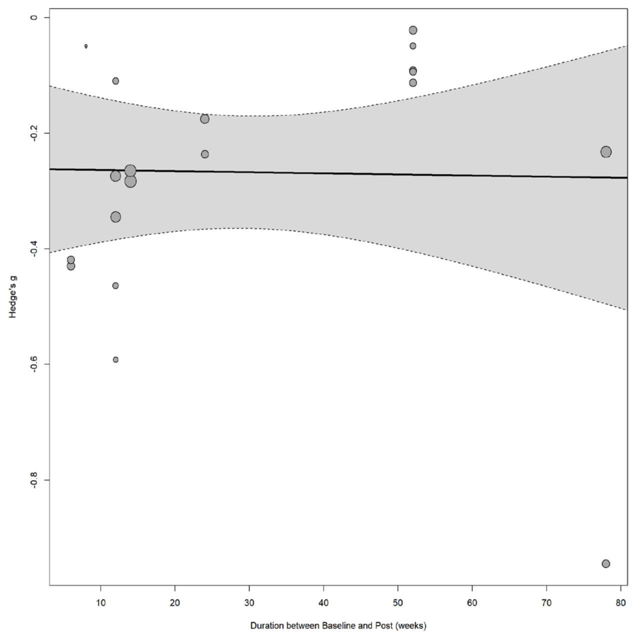

Figure S2: Eating disorder risk [Baseline - Post] meta regression
The predicted change in eating disorder risk between baseline and post (Hedge's g) as a function of intervention duration (weeks) using a mixed effects meta-regression. The grey area captures the bounds of the corresponding 95% confidence interval. Each study estimate is captured in a bubble with a size proportional to its study weight (test of moderators, Q_M (df = 2) = 0.0087; moderator (duration) beta: -0.00019; Q_m pvalue: 0.92584).

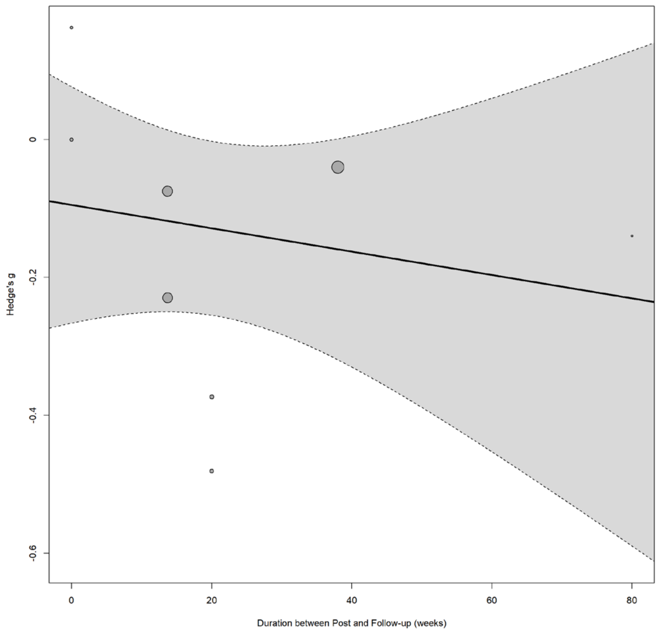

Figure S3: Eating disorder risk [Post - Follow-up] meta regression
The predicted change in eating disorder risk between post and follow-up (Hedge's g) as a function of follow-up duration (weeks) using a mixed effects meta-regression. The grey area captures the bounds of the corresponding 95% confidence interval. Each study estimate is captured in a bubble with a size proportional to its study weight (test of moderators, Q_M (df = 2) = 0.3446; Moderator time beta: -0.00169; Q_m pvalue: 0.55719).

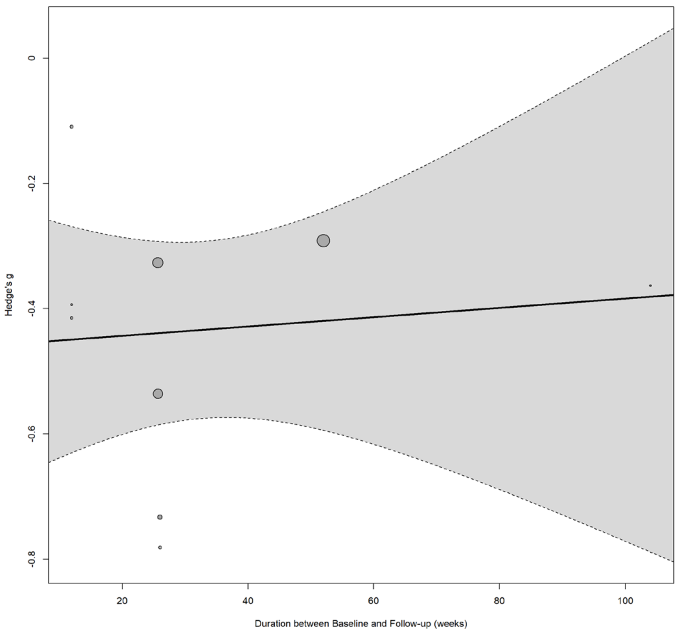

Figure S4: Eating disorder risk [Baseline - Follow-up] meta regression
The predicted change in eating disorder risk between baseline and follow-up (Hedge's g) as a function of duration (weeks) using a mixed effects meta-regression. The grey area captures the bounds of the corresponding 95% confidence interval. Each study estimate is captured in a bubble with a size proportional to its study weight (test of moderators, Q_M (df = 2) = 0.0735; Moderator time beta: 0.00074; Q_m pvalue: 0.78635).

Study	Duration	Tool	N	Baseline (M,SD)	Post (M,SD)	Difference (M,SD)	Weight	Estimate [95% CI]
**Baseline and post scores**								
Barnes et al. 2014 IG1	12	EDE	30	2.1 (0.9)	1.7 (0.8)		3.38%	-0.46 [-0.90, -0.02]
Barnes et al. 2014 IG2	12	EDE	29	1.6 (0.8)	1.2 (0.5)		3.19%	-0.59 [-1.05, -0.14]
Barnes et al. 2014 IG3	12	EDE	30	1.6 (0.9)	1.5 (0.9)		3.50%	-0.11 [-0.54, 0.32]
Dassen et al. 2018 IG1	6	EDE-Q	34	2.56 (0.78)	2.24 (0.67)		4.58%	-0.43 [-0.80, -0.06]
Dassen et al. 2018 IG2	6	EDE-Q	36	2.3 (0.86)	1.98 (0.62)		4.27%	-0.42 [-0.81, -0.03]
Jospe et al. 2017 IG1	52	EDE-Q	38	2.19 (0.9)	2.17 (0.92)		4.40%	-0.02 [-0.40, 0.36]
Jospe et al. 2017 IG2	52	EDE-Q	36	2.15 (0.9)	2.04 (1.03)		4.17%	-0.11 [-0.51, 0.28]
Jospe et al. 2017 IG3	52	EDE-Q	32	1.7 (0.83)	1.62 (0.86)		3.73%	-0.09 [-0.51, 0.32]
Jospe et al. 2017 IG4	52	EDE-Q	28	1.89 (0.99)	1.84 (1.02)		3.28%	-0.05 [-0.50, 0.40]
Jospe et al. 2017 IG5	52	EDE-Q	35	1.97 (0.92)	1.88 (1.02)		4.06%	-0.09 [-0.49, 0.31]
LaRose et al. 2014 IG1	78	EDDS	105	18.82 (8.62)	11.54 (7.38)		4.78%	-0.95 [-1.31, -0.58]
LaRose et al. 2014 IG2	78	EDDS	49	16.08 (8.52)	14.14 (7.7)		8.02%	-0.23 [-0.51, 0.04]
Mensinger et al. 2016 IG1	24	EDE-Q	33	2.35 (0.7)	2.19 (0.63)		4.16%	-0.24 [-0.63, 0.16]
Moss et al. 2017 IG1	12	EDE-Q	69	2.27 (1.16)	2.01 (0.66)		7.51%	-0.27 [-0.56, 0.01]
Moss et al. 2017 IG2	12	EDE-Q	66	2.34 (0.89)	2.07 (0.65)		7.18%	-0.34 [-0.64, -0.05]
Steinberg et al. 2014 IG1	24	ACS	45	32.9 (6.86)	31.7 (6.71)		5.24%	-0.18 [-0.52, 0.17]
Werrij et al. 2009 IG1	14	EDE-Q	96	2.36 (0.94)	2.11 (0.94)		9.97%	-0.26 [-0.50, -0.03]
Werrij et al. 2009 IG2	14	EDE-Q	104	2.04 (0.99)	1.77 (0.91)		10.63%	-0.28 [-0.51, -0.05]
RE Model for Subgroup (Q = 22.64, df = 17, p = 0.16; I^2 = 13.7%)								-0.29 [-0.37, -0.20]
**Difference scores**								
Christaki et al. 2013 IG1	8	EAT-26	18			-0.6 (9.33)	2.09%	-0.07 [-0.64, 0.49]
Christaki et al. 2013 IG2	8	EAT-26	16			-0.6 (8.8)	1.85%	-0.08 [-0.68, 0.53]
RE Model for Subgroup (Q = 0.00, df = 1, p = 0.99; I^2 = 0.0%)								-0.08 [-0.49, 0.34]
Test for Subgroup Differences: Q_M = 0.91, df = 1, p = 0.34, Pooled I^2 = 9.9							100.00%	-0.28 [-0.36, -0.19]

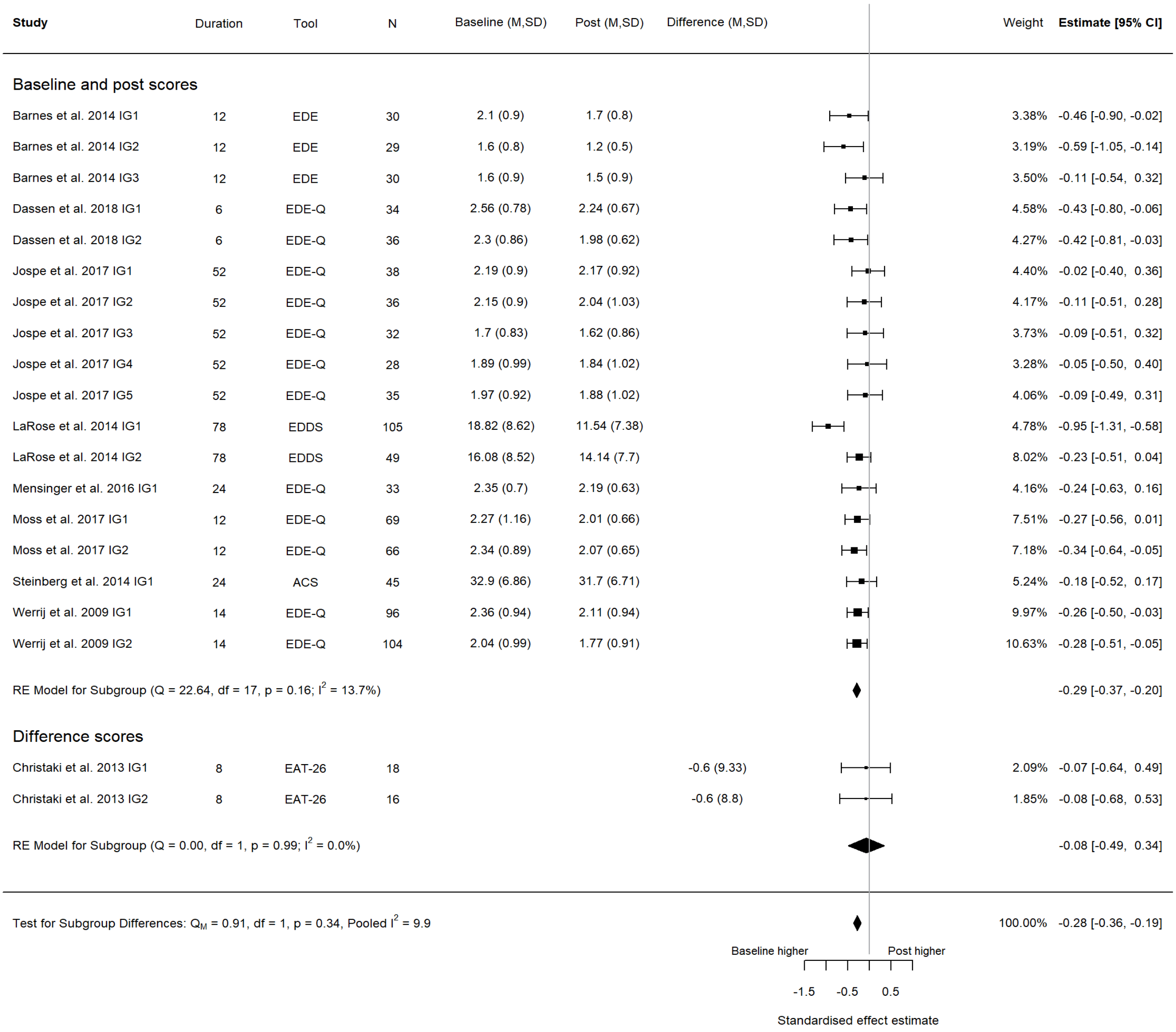

Figure S5: Eating disorder risk [Baseline - Post] assuming a correlation of 0.3
Forest plot of the change in eating disorder risk from baseline to post for each trial. Each estimate was standardized using Hedges g. A correlation of 0.3 was assumed between time points when necessary for the calculation of Hedges' g. A random effects model was used to combine estimates from each trial (prediction lower bound: -0.42, Prediction upper bound: -0.14, Tau^2: 0.0033).

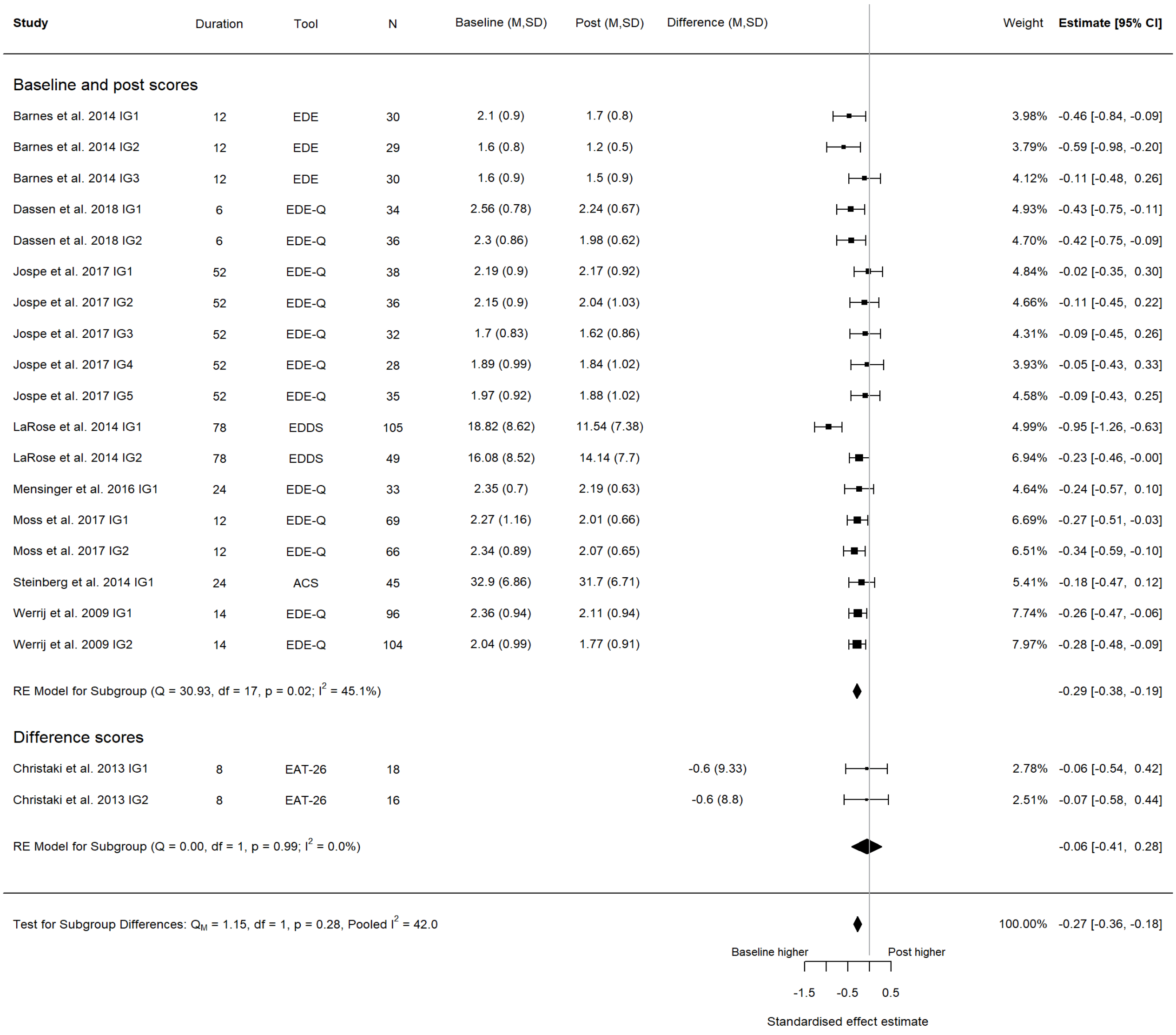

Figure S6: Eating disorder risk [Baseline - Post] assuming a correlation of 0.5

Forest plot of the change in eating disorder risk from baseline to post for each trial. Each estimate was standardized using Hedges g. A correlation of 0.5 was assumed between time points when necessary for the calculation of Hedges' g. A random effects model was used to combine estimates from each trial (prediction lower bound: -0.54, Prediction upper bound: 0, Tau^2: 0.0167).

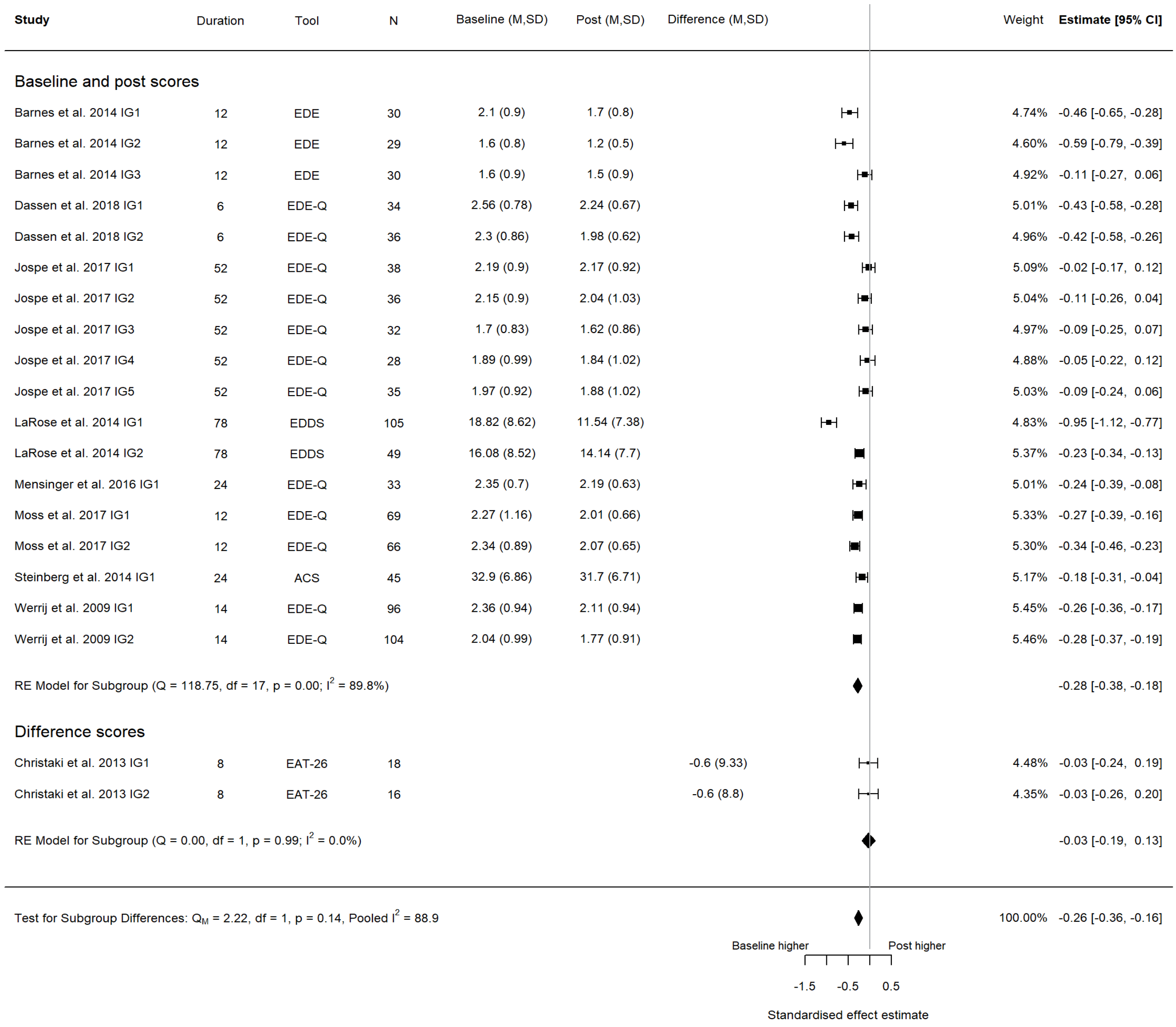

Figure S7: Eating disorder risk [Baseline - Post] assuming a correlation of 0.9

Forest plot of the change in eating disorder risk from baseline-post for each trial. Each estimate was standardized using Hedges g. A correlation of 0.9 was assumed between time points when necessary for the calculation of Hedges' g. A random effects model was used to combine estimates from each trial (Prediction lower bound: -0.67, Prediction upper bound: 0.16, Tau^2: 0.0424).

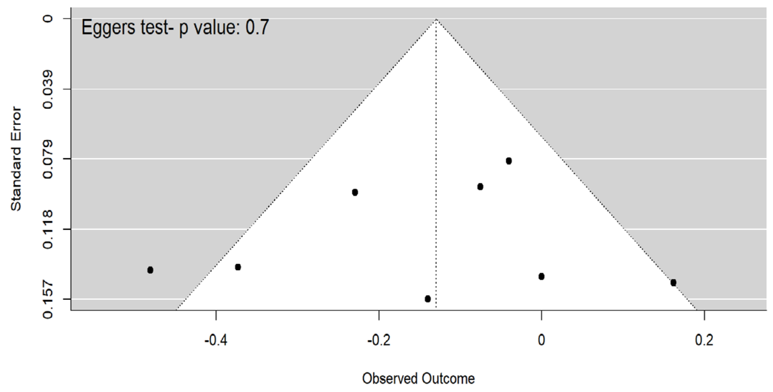

Figure S8: Eating disorder risk [Post - Follow-up] funnel plot
Funnel plot with the standardized change (Hedges' g) in eating disorder risk between post and follow-up on the x axis and standard error on the y axis.

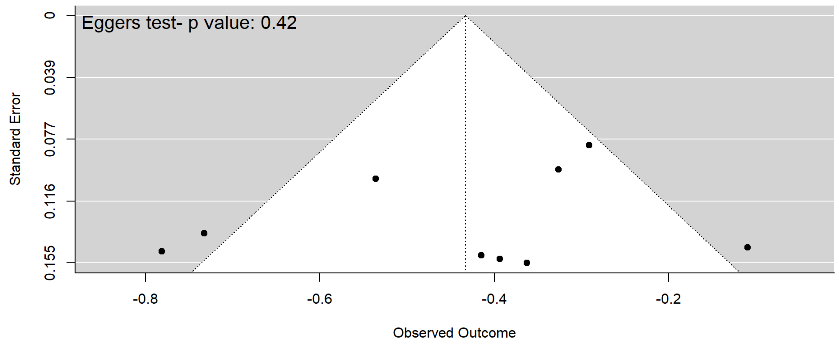

Figure S9: Eating disorder risk [Baseline - Follow-up] funnel plot
Funnel plot with the standardized change (Hedges' g) in eating disorder risk between baseline and follow-up on the x axis and standard error on the y axis.

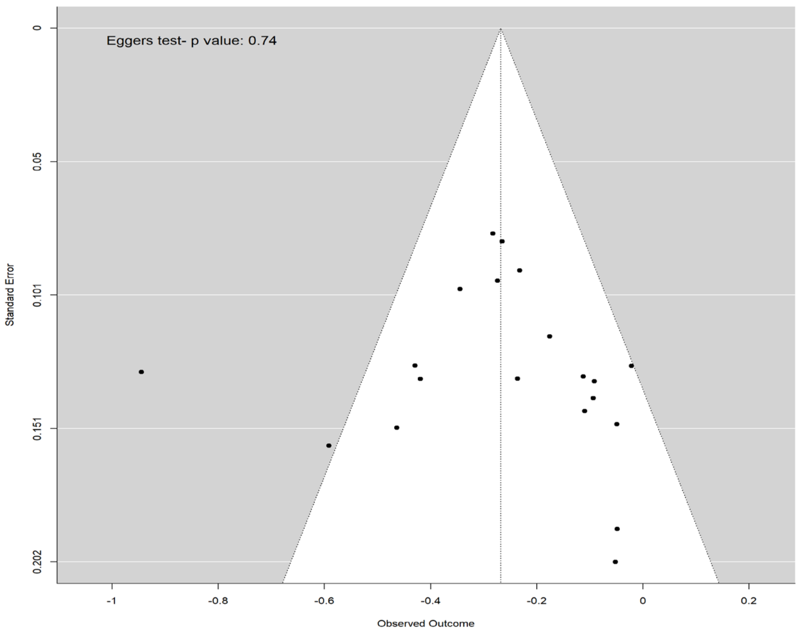

Figure S10: Eating disorder risk [Baseline - Post] funnel plot
Funnel plot with the standardized change (Hedges' g) in eating disorder risk between baseline and post on the x axis and standard error on the y axis.

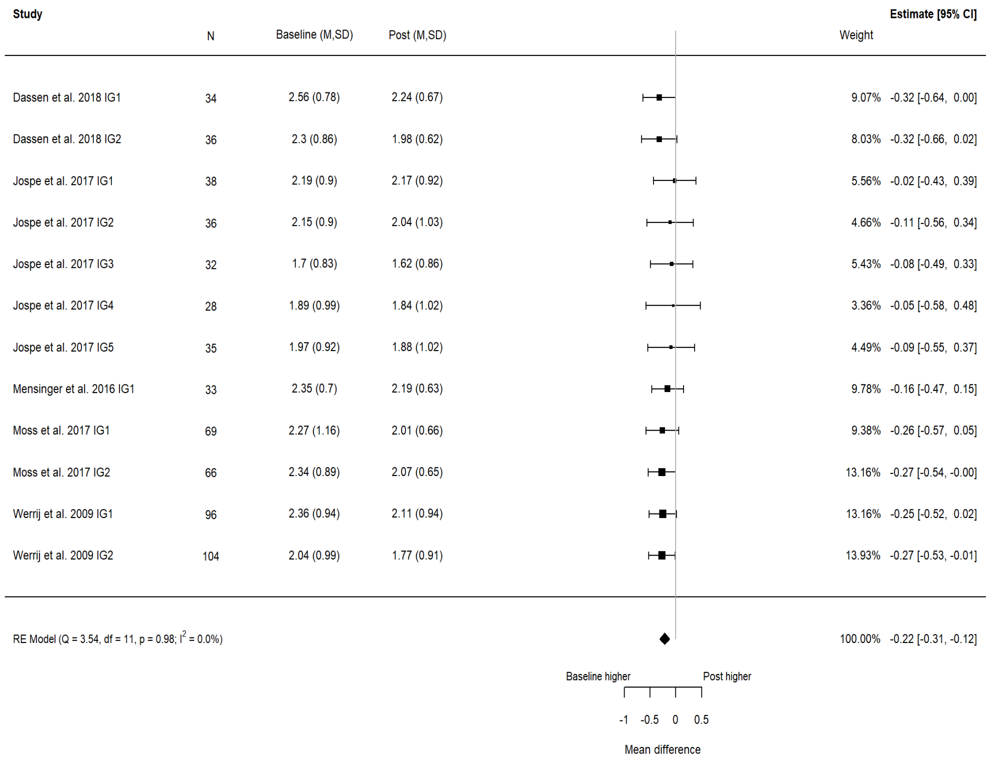

Figure S11: EDE-Q scale only [Baseline - Post] forest plot
Forest plot that only includes measurements of eating disorder risk that have been assessed with the EDE-Q tool. Raw scores were used to calculate a mean difference between baseline and post for each trial and a random effects model was used to combine estimates from each trial.

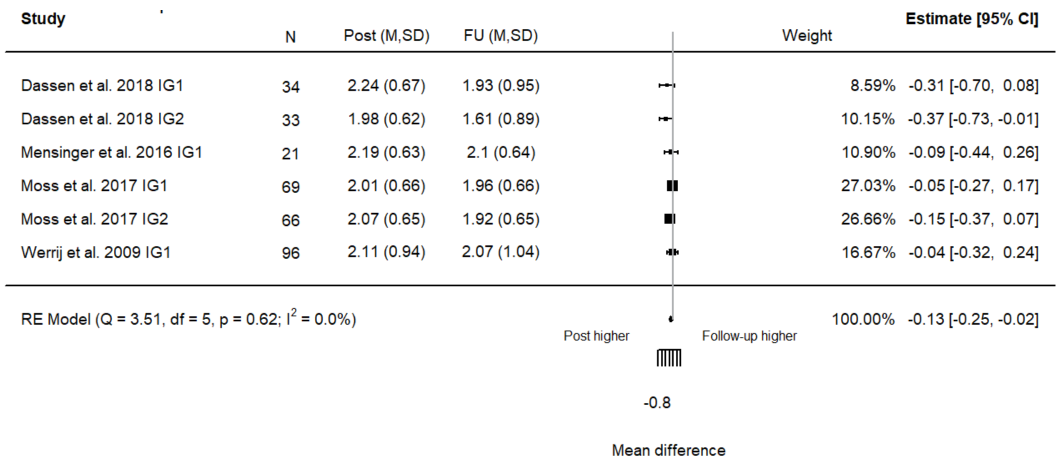

Figure S12: EDE-Q scale only [Post - Follow-up] forest plot
Forest plot that only includes measurements of eating disorder risk that have been assessed with the EDE-Q tool. Raw scores were used to calculate a mean difference between post and follow-up for each trial and a random effects model was used to combine estimates from each trial.

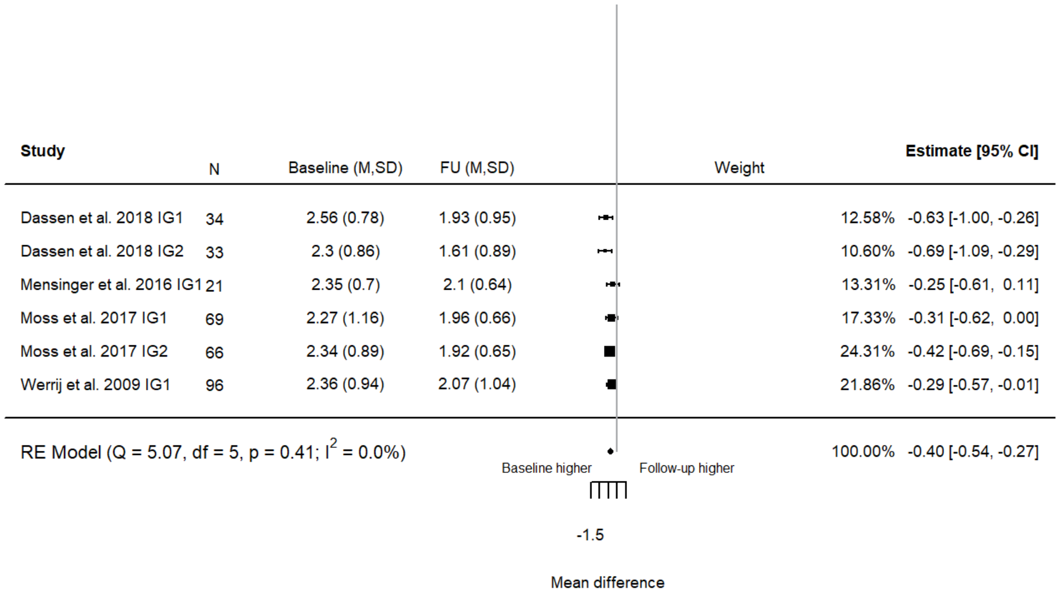

Figure S13: EDE-Q scale only [Baseline - Follow-up] forest plot
Forest plot that only includes measurements of eating disorder risk that have been assessed with the EDE-Q tool. Raw scores were used to calculate a mean difference between baseline and follow-up for each trial and a random effects model was used to combine estimates from each trial.

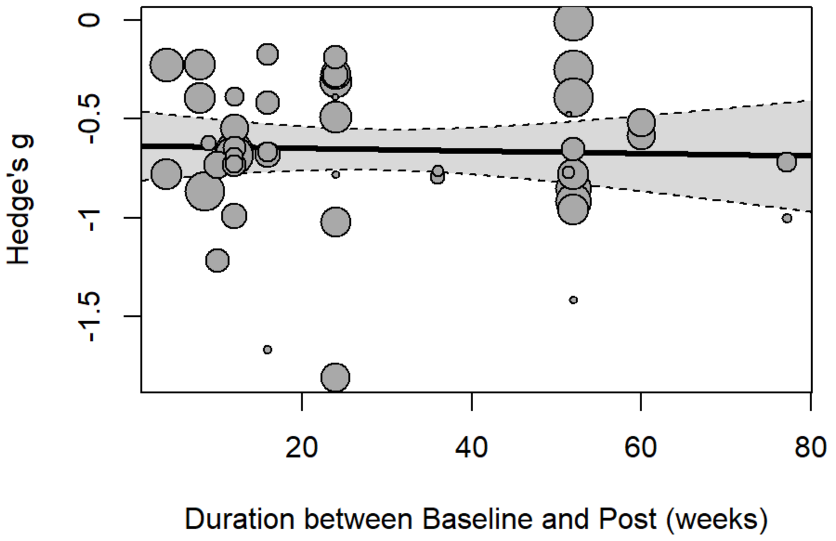

Figure S14: Binge eating [Baseline - Post] meta regression
The predicted change in binge eating between baseline and post (Hedge's g) as a function of intervention duration (weeks) using a mixed effects meta-regression. The grey area captures the bounds of the corresponding 95% confidence interval. Each study estimate is captured in a bubble with a size proportional to its study weight (test of moderators, Q_M (df = 2) = 0.0612; Moderator time beta: -0.00064; Q_m pvalue: 0.80461).

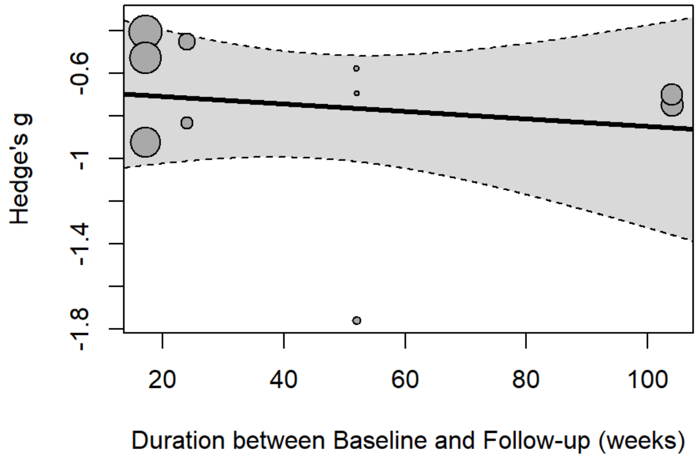

Figure S15: Binge eating [Baseline - Follow-up] meta regression
The predicted change in binge eating between baseline and follow-up (Hedge's g) as a function of duration (weeks) using a mixed effects meta-regression. The grey area captures the bounds of the corresponding 95% confidence interval. Each study estimate is captured in a bubble with a size proportional to its study weight (test of moderators, Q_M (df = 2) = 0.2075; Moderator time beta: -0.00175; Q_m pvalue: 0.64872).

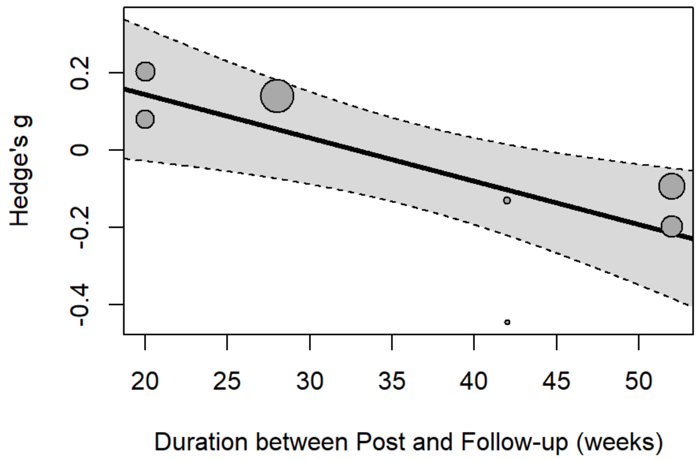

Figure S16: Binge eating [Post - Follow-up] meta regression
The predicted change in binge eating between baseline and post (Hedge's g) as a function of follow-up duration (weeks) using a mixed effects meta-regression. The grey area captures the bounds of the corresponding 95% confidence interval. Each study estimate is captured in a bubble with a size proportional to its study weight (test of moderators, Q_M (df = 2) = 7.1583; Moderator time beta: -0.01122; Q_m pvalue: 0.00746).

Study	Duration	Tool	N	Baseline (M,SD)	Post (M,SD)	Difference (M,SD)	Weight	Estimate [95% CI]
**Baseline and post scores**								
Afari et al. 2019 IG1	4	BES	43	15.7 (9.2)	13.6 (9)		2.40%	-0.23 [-0.59, 0.13]
Afari et al. 2019 IG2	4	BES	42	16.8 (8.5)	10.6 (7.2)		2.31%	-0.78 [-1.16, -0.40]
Ariel 2016 IG3	8.56	BES	151	15 (8)	8.7 (6.4)		2.97%	-0.87 [-1.07, -0.67]
Beaulieu et al. 2020 IG1	12	BES	24	15 (9)	9 (7)		1.87%	-0.73 [-1.24, -0.22]
Beaulieu et al. 2020 IG2	12	BES	22	16 (7)	12 (5)		1.81%	-0.65 [-1.17, -0.12]
Carels et al. 2014 IG1	12	BES	29	22 (10.8)	16.6 (8.5)		2.06%	-0.55 [-1.00, -0.10]
Carels et al. 2014 IG2	12	BES	30	23.1 (8.8)	15 (7.2)		2.00%	-0.99 [-1.47, -0.52]
Carels et al. 2019 IG1	16	BES	19	36.6 (4.9)	32.6 (6.7)		1.67%	-0.67 [-1.24, -0.09]
Carels et al. 2019 IG2	16	BES	21	35 (7.3)	32 (6.7)		1.80%	-0.42 [-0.95, 0.11]
Carels et al. 2019 IG3	16	BES	26	32.3 (8.8)	27 (6.3)		1.94%	-0.68 [-1.17, -0.20]
Carpenter et al. 2019 IG1	24	BES	42	19.2 (6.8)	11.5 (8.1)		2.26%	-1.02 [-1.42, -0.62]
Carpenter et al. 2019 IG2	24	BES	22	18 (7.5)	15.9 (7.3)		1.86%	-0.28 [-0.79, 0.23]
Dalle et al 2013 IG1	52	BES	43	13.5 (9.8)	6.9 (6.7)		2.33%	-0.78 [-1.16, -0.40]
Dalle et al 2013 IG2	52	BES	45	13.9 (9.6)	5.8 (6.9)		2.33%	-0.96 [-1.34, -0.58]
Dennis et al. 1999 IG1	16	BES	21	18 (8)	8.2 (1.5)		1.47%	-1.67 [-2.32, -1.02]
Dennis et al. 1999 IG2	16	BES	18	12.5 (7.7)	11.5 (1.8)		1.69%	-0.17 [-0.74, 0.39]
Dennis et al. 2001 IG1	24	BES	20	11.7 (7.6)	10.4 (5.9)		1.78%	-0.19 [-0.72, 0.35]
Dennis et al. 2001 IG3	24	BES	10	21.8 (11.1)	17.8 (8.3)		1.14%	-0.39 [-1.19, 0.41]
Dennis et al. 2001 IG4	24	BES	12	17.8 (8.8)	10.9 (8.2)		1.23%	-0.78 [-1.53, -0.03]
Glynn et al. 2022 IG1	12	BES	103	10.7 (5.07)	7.3 (5.07)		2.85%	-0.67 [-0.91, -0.43]
Glynn et al. 2022 IG2	12	BES	103	11.1 (5.07)	7.6 (5.07)		2.85%	-0.69 [-0.93, -0.45]
Goodrick et al. 1998 IG1	24	BES	65	27.82 (6.13)	15.42 (7.42)		2.37%	-1.81 [-2.18, -1.44]
Mason et al. 2019 IG1	52	BES	101	4.59 (2.97)	3.5 (2.56)		2.89%	-0.39 [-0.62, -0.16]
Mason et al. 2019 IG2	52	BES	98	3.51 (2.84)	3.49 (2.66)		2.89%	-0.01 [-0.23, 0.22]
Mason et al. 2019 IG3	52	BES	104	3.91 (2.58)	3.28 (2.42)		2.90%	-0.25 [-0.47, -0.03]
Rapoport et al. 2000 IG1	10	BES	31	14 (9)	8 (7)		2.08%	-0.73 [-1.18, -0.29]
Rapoport et al. 2000 IG2	10	BES	31	15 (9)	6 (5)		1.98%	-1.22 [-1.69, -0.74]
Raynor et al. 2006 IG1 & IG2	9	BES	15	16.3 (7.7)	11.6 (7)		1.46%	-0.62 [-1.27, 0.03]
Rieger et al. 2017 IG1	52	BES	58	18.1 (8.11)	11.41 (7.34)		2.66%	-0.85 [-1.14, -0.56]
Rieger et al. 2017 IG2	52	BES	54	17.01 (7.67)	10.37 (6.28)		2.63%	-0.92 [-1.22, -0.62]
Smith et al. 2018 IG1.1	36	BES	14	16.94 (8.25)	10.57 (7.23)		1.48%	-0.79 [-1.44, -0.15]
Smith et al. 2018 IG2.1	36	BES	11	12.66 (7.42)	7.27 (5.81)		1.36%	-0.76 [-1.45, -0.07]
Wadden et al. 1993 IG1	52	BES	17	22.88 (8.18)	12 (6.78)		1.37%	-1.41 [-2.10, -0.72]
Wadden et al. 1993 IG2	52	BES	23	23.46 (7.27)	18.32 (8.18)		1.84%	-0.65 [-1.17, -0.14]
Zwickert et al. 2016 IG1	60	BES	31	18.9 (7.79)	13.4 (10.58)		2.11%	-0.58 [-1.02, -0.15]
Zwickert et al. 2016 IG2	60	BES	29	18.1 (8.08)	13.1 (10.77)		2.06%	-0.52 [-0.97, -0.07]
RE Model for Subgroup (Q = 139.61, df = 35, p = 0.00; I^2 = 73.7%)								-0.70 [-0.83, -0.57]
**Difference scores**								
Cheng et al. 2014 IG1	51.43	BES	21			-5.38 (6.83)	1.70%	-0.91 [-1.48, -0.35]
Cheng et al. 2014 IG2	51.43	BES	15			-3.93 (8.06)	1.48%	-0.56 [-1.21, 0.08]
Fogelholm et al. 1999 IG1	12	BITE	26			-2.5 (3.57)	1.92%	-0.82 [-1.31, -0.32]
Fogelholm et al. 1999 IG2	12	BITE	26			-1.2 (3.06)	1.98%	-0.46 [-0.93, 0.02]
Fogelholm et al. 1999 IG3	12	BITE	26			-3.4 (4.59)	1.91%	-0.86 [-1.36, -0.37]
Keranen et al. 2009 IG1	77.14	BES	20			-7 (6.85)	1.59%	-1.19 [-1.79, -0.58]
Keranen et al. 2009 IG2	77.14	BES	29			-5 (6.87)	2.00%	-0.85 [-1.32, -0.38]
Whitelock et al. 2019 IG1	8	BES	53			-1.3 (5.7)	2.53%	-0.27 [-0.59, 0.06]
Whitelock et al. 2019 IG2	8	BES	54			-2.3 (5.8)	2.53%	-0.47 [-0.79, -0.14]
Martin et al. 2019 IG1	24	MAEDS	59			-1.97 (6.31)	2.59%	-0.37 [-0.68, -0.06]
Martin et al. 2019 IG2	24	MAEDS	51			-3.42 (12.44)	2.50%	-0.32 [-0.65, 0.01]
Martin et al. 2019 IG3	24	MAEDS	61			-3.07 (6.24)	2.59%	-0.58 [-0.89, -0.27]
RE Model for Subgroup (Q = 16.51, df = 11, p = 0.12; I^2 = 31.9%)								-0.57 [-0.71, -0.42]
Test for Subgroup Differences: Q_M = 0.67, df = 1, p = 0.41, Pooled I^2 = 69.2							100.00%	-0.68 [-0.78, -0.57]

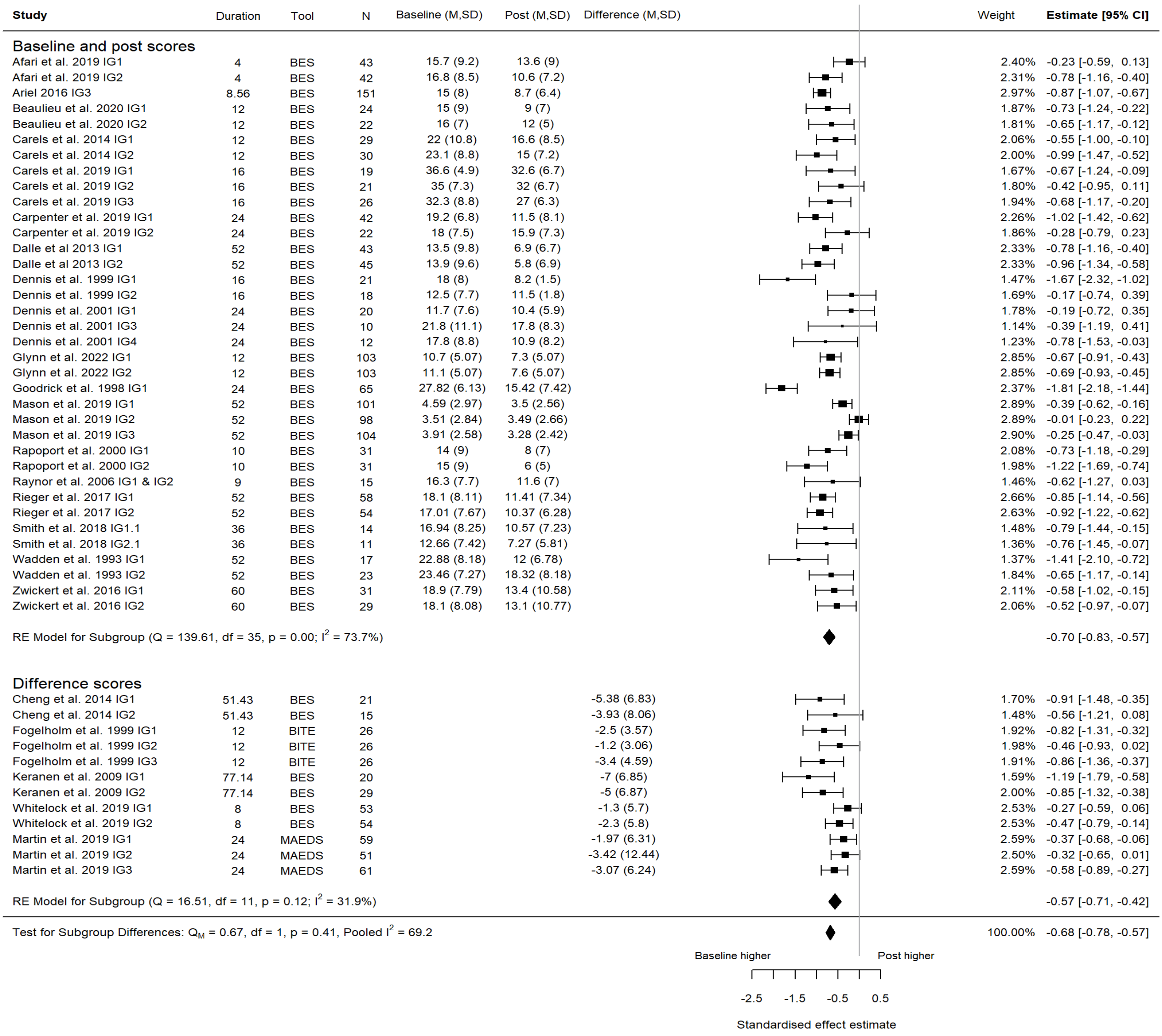

Figure S17: Binge eating [Baseline - Post] assuming a correlation of 0.3
Forest plot of the change in binge eating from baseline-post for each trial. Each estimate was standardized using Hedges g. A correlation of 0.3 was assumed between time points when necessary for the calculation of Hedges' g. A random effects model was used to combine estimates from each trial (prediction lower bound: -1.26, Prediction upper bound: -0.09, Tau^2: 0.0855).

Study	Duration	Tool	N	Baseline (M,SD)	Post (M,SD)	Difference (M,SD)	Weight	Estimate [95% CI]
**Baseline and post scores**								
Afari et al. 2019 IG1	4	BES	43	15.7 (9.2)	13.6 (9)		2.35%	-0.23 [-0.53, 0.08]
Afari et al. 2019 IG2	4	BES	42	16.8 (8.5)	10.6 (7.2)		2.27%	-0.78 [-1.11, -0.45]
Ariel 2016 IG3	8.56	BES	151	15 (8)	8.7 (6.4)		2.73%	-0.87 [-1.04, -0.69]
Beaulieu et al. 2020 IG1	12	BES	24	15 (9)	9 (7)		1.93%	-0.73 [-1.17, -0.29]
Beaulieu et al. 2020 IG2	12	BES	22	16 (7)	12 (5)		1.88%	-0.65 [-1.10, -0.19]
Carels et al. 2014 IG1	12	BES	29	22 (10.8)	16.6 (8.5)		2.09%	-0.55 [-0.93, -0.16]
Carels et al. 2014 IG2	12	BES	30	23.1 (8.8)	15 (7.2)		2.02%	-0.99 [-1.40, -0.58]
Carels et al. 2019 IG1	16	BES	19	36.6 (4.9)	32.6 (6.7)		1.77%	-0.67 [-1.16, -0.17]
Carels et al. 2019 IG2	16	BES	21	35 (7.3)	32 (6.7)		1.89%	-0.42 [-0.87, 0.03]
Carels et al. 2019 IG3	16	BES	26	32.3 (8.8)	27 (6.3)		1.99%	-0.68 [-1.10, -0.26]
Carpenter et al. 2019 IG1	24	BES	42	19.2 (6.8)	11.5 (8.1)		2.22%	-1.02 [-1.37, -0.67]
Carpenter et al. 2019 IG2	24	BES	22	18 (7.5)	15.9 (7.3)		1.94%	-0.28 [-0.71, 0.16]
Dalle et al 2013 IG1	52	BES	43	13.5 (9.8)	6.9 (6.7)		2.28%	-0.78 [-1.11, -0.45]
Dalle et al 2013 IG2	52	BES	45	13.9 (9.6)	5.8 (6.9)		2.27%	-0.96 [-1.29, -0.63]
Dennis et al. 1999 IG1	16	BES	21	18 (8)	8.2 (1.5)		1.52%	-1.67 [-2.25, -1.09]
Dennis et al. 1999 IG2	16	BES	18	12.5 (7.7)	11.5 (1.8)		1.80%	-0.17 [-0.66, 0.31]
Dennis et al. 2001 IG1	24	BES	20	11.7 (7.6)	10.4 (5.9)		1.88%	-0.19 [-0.64, 0.27]
Dennis et al. 2001 IG3	24	BES	10	21.8 (11.1)	17.8 (8.3)		1.29%	-0.39 [-1.07, 0.29]
Dennis et al. 2001 IG4	24	BES	12	17.8 (8.8)	10.9 (8.2)		1.36%	-0.78 [-1.43, -0.13]
Glynn et al. 2022 IG1	12	BES	103	10.7 (5.07)	7.3 (5.07)		2.65%	-0.67 [-0.87, -0.46]
Glynn et al. 2022 IG2	12	BES	103	11.1 (5.07)	7.6 (5.07)		2.65%	-0.69 [-0.89, -0.48]
Goodrick et al. 1998 IG1	24	BES	65	27.82 (6.13)	15.42 (7.42)		2.26%	-1.81 [-2.14, -1.48]
Mason et al. 2019 IG1	52	BES	101	4.59 (2.97)	3.5 (2.56)		2.69%	-0.39 [-0.58, -0.20]
Mason et al. 2019 IG2	52	BES	98	3.51 (2.84)	3.49 (2.66)		2.69%	-0.01 [-0.20, 0.18]
Mason et al. 2019 IG3	52	BES	104	3.91 (2.58)	3.28 (2.42)		2.69%	-0.25 [-0.44, -0.06]
Rapoport et al. 2000 IG1	10	BES	31	14 (9)	8 (7)		2.10%	-0.73 [-1.12, -0.35]
Rapoport et al. 2000 IG2	10	BES	31	15 (9)	6 (5)		1.99%	-1.22 [-1.64, -0.80]
Raynor et al. 2006 IG1 & IG2	9	BES	15	16.3 (7.7)	11.6 (7)		1.59%	-0.62 [-1.18, -0.07]
Rieger et al. 2017 IG1	52	BES	58	18.1 (8.11)	11.41 (7.34)		2.52%	-0.85 [-1.10, -0.60]
Rieger et al. 2017 IG2	52	BES	54	17.01 (7.67)	10.37 (6.28)		2.50%	-0.92 [-1.18, -0.66]
Smith et al. 2018 IG1.1	36	BES	14	16.94 (8.25)	10.57 (7.23)		1.59%	-0.79 [-1.35, -0.24]
Smith et al. 2018 IG2.1	36	BES	11	12.66 (7.42)	7.27 (5.81)		1.49%	-0.76 [-1.36, -0.17]
Wadden et al. 1993 IG1	52	BES	17	22.88 (8.18)	12 (6.78)		1.44%	-1.41 [-2.03, -0.80]
Wadden et al. 1993 IG2	52	BES	23	23.46 (7.27)	18.32 (8.18)		1.91%	-0.65 [-1.10, -0.21]
Zwickert et al. 2016 IG1	60	BES	31	18.9 (7.79)	13.4 (10.58)		2.13%	-0.58 [-0.96, -0.21]
Zwickert et al. 2016 IG2	60	BES	29	18.1 (8.08)	13.1 (10.77)		2.10%	-0.52 [-0.90, -0.13]
RE Model for Subgroup (Q = 183.85, df = 35, p = 0.00; I^2 = 80.2%)								-0.70 [-0.83, -0.57]
**Difference scores**								
Cheng et al. 2014 IG1	51.43	BES	21			-5.38 (6.83)	1.82%	-0.77 [-1.25, -0.30]
Cheng et al. 2014 IG2	51.43	BES	15			-3.93 (8.06)	1.62%	-0.47 [-1.02, 0.07]
Fogelholm et al. 1999 IG1	12	BITE	26			-2.5 (3.57)	1.99%	-0.69 [-1.11, -0.27]
Fogelholm et al. 1999 IG2	12	BITE	26			-1.2 (3.06)	2.04%	-0.39 [-0.79, 0.02]
Fogelholm et al. 1999 IG3	12	BITE	26			-3.4 (4.59)	1.98%	-0.73 [-1.15, -0.31]
Keranen et al. 2009 IG1	77.14	BES	20			-7 (6.85)	1.72%	-1.00 [-1.51, -0.49]
Keranen et al. 2009 IG2	77.14	BES	29			-5 (6.87)	2.06%	-0.72 [-1.11, -0.32]
Whitelock et al. 2019 IG1	8	BES	53			-1.3 (5.7)	2.45%	-0.23 [-0.50, 0.05]
Whitelock et al. 2019 IG2	8	BES	54			-2.3 (5.8)	2.45%	-0.39 [-0.67, -0.12]
Martin et al. 2019 IG1	24	MAEDS	59			-1.97 (6.31)	2.49%	-0.31 [-0.57, -0.05]
Martin et al. 2019 IG2	24	MAEDS	51			-3.42 (12.44)	2.43%	-0.27 [-0.55, 0.01]
Martin et al. 2019 IG3	24	MAEDS	61			-3.07 (6.24)	2.49%	-0.49 [-0.75, -0.23]
RE Model for Subgroup (Q = 16.51, df = 11, p = 0.12; I^2 = 31.9%)								-0.48 [-0.60, -0.36]
Test for Subgroup Differences: Q_M = 2.47, df = 1, p = 0.12, Pooled I^2 = 75.9							100.00%	-0.65 [-0.76, -0.55]

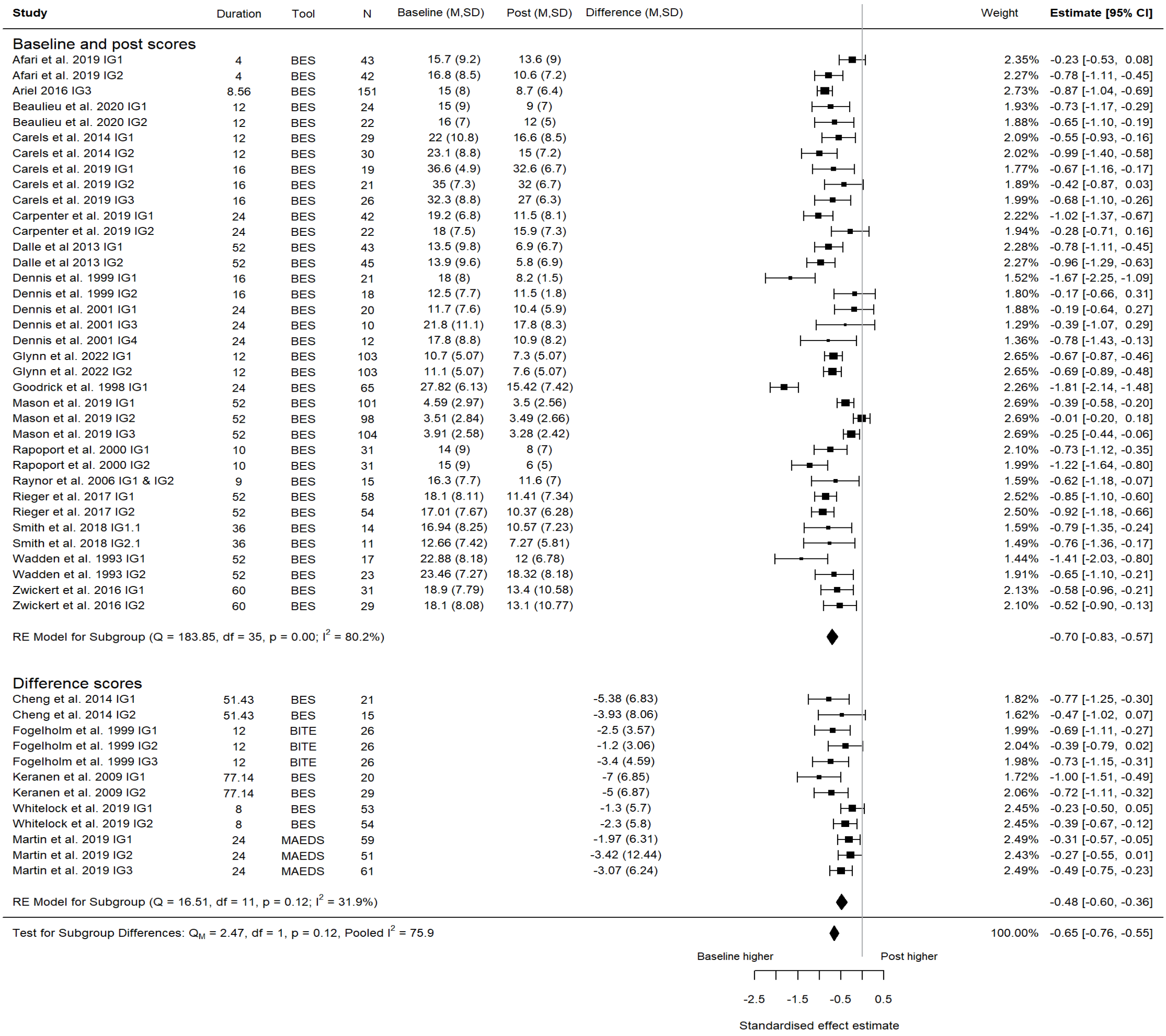

Figure S18: Binge eating [Baseline - Post] assuming a correlation of 0.5

Forest plot of the change in binge eating from baseline-post for each trial. Each estimate was standardized using Hedges g. A correlation of 0.5 was assumed between time points when necessary for the calculation of Hedges' g. A random effects model was used to combine estimates from each trial (Prediction lower bound: -1.26, Prediction upper bound: -0.05, Tau^2: 0.0929).

Study	Duration	Tool	N	Baseline (M,SD)	Post (M,SD)	Difference (M,SD)	Weight	Estimate [95% CI]
**Baseline and post scores**								
Afari et al. 2019 IG1	4	BES	43	15.7 (9.2)	13.6 (9)		2.19%	-0.23 [-0.37, -0.09]
Afari et al. 2019 IG2	4	BES	42	16.8 (8.5)	10.6 (7.2)		2.13%	-0.78 [-0.96, -0.60]
Ariel 2016 IG3	8.56	BES	151	15 (8)	8.7 (6.4)		2.23%	-0.87 [-0.97, -0.77]
Beaulieu et al. 2020 IG1	12	BES	24	15 (9)	9 (7)		2.04%	-0.73 [-0.97, -0.49]
Beaulieu et al. 2020 IG2	12	BES	22	16 (7)	12 (5)		2.04%	-0.65 [-0.89, -0.41]
Carels et al. 2014 IG1	12	BES	29	22 (10.8)	16.6 (8.5)		2.11%	-0.55 [-0.74, -0.35]
Carels et al. 2014 IG2	12	BES	30	23.1 (8.8)	15 (7.2)		2.03%	-0.99 [-1.24, -0.75]
Carels et al. 2019 IG1	16	BES	19	36.6 (4.9)	32.6 (6.7)		2.00%	-0.67 [-0.93, -0.40]
Carels et al. 2019 IG2	16	BES	21	35 (7.3)	32 (6.7)		2.08%	-0.42 [-0.64, -0.20]
Carels et al. 2019 IG3	16	BES	26	32.3 (8.8)	27 (6.3)		2.07%	-0.68 [-0.91, -0.46]
Carpenter et al. 2019 IG1	24	BES	42	19.2 (6.8)	11.5 (8.1)		2.09%	-1.02 [-1.23, -0.81]
Carpenter et al. 2019 IG2	24	BES	22	18 (7.5)	15.9 (7.3)		2.11%	-0.28 [-0.48, -0.08]
Dalle et al 2013 IG1	52	BES	43	13.5 (9.8)	6.9 (6.7)		2.14%	-0.78 [-0.96, -0.60]
Dalle et al 2013 IG2	52	BES	45	13.9 (9.6)	5.8 (6.9)		2.11%	-0.96 [-1.16, -0.76]
Dennis et al. 1999 IG1	16	BES	21	18 (8)	8.2 (1.5)		1.67%	-1.67 [-2.10, -1.24]
Dennis et al. 1999 IG2	16	BES	18	12.5 (7.7)	11.5 (1.8)		2.08%	-0.17 [-0.39, 0.04]
Dennis et al. 2001 IG1	24	BES	20	11.7 (7.6)	10.4 (5.9)		2.10%	-0.19 [-0.39, 0.02]
Dennis et al. 2001 IG3	24	BES	10	21.8 (11.1)	17.8 (8.3)		1.88%	-0.39 [-0.72, -0.06]
Dennis et al. 2001 IG4	24	BES	12	17.8 (8.8)	10.9 (8.2)		1.80%	-0.78 [-1.15, -0.42]
Glynn et al. 2022 IG1	12	BES	103	10.7 (5.07)	7.3 (5.07)		2.22%	-0.67 [-0.78, -0.56]
Glynn et al. 2022 IG2	12	BES	103	11.1 (5.07)	7.6 (5.07)		2.22%	-0.69 [-0.80, -0.58]
Goodrick et al. 1998 IG1	24	BES	65	27.82 (6.13)	15.42 (7.42)		2.03%	-1.81 [-2.06, -1.56]
Mason et al. 2019 IG1	52	BES	101	4.59 (2.97)	3.5 (2.56)		2.23%	-0.39 [-0.48, -0.30]
Mason et al. 2019 IG2	52	BES	98	3.51 (2.84)	3.49 (2.66)		2.24%	-0.01 [-0.09, 0.08]
Mason et al. 2019 IG3	52	BES	104	3.91 (2.58)	3.28 (2.42)		2.24%	-0.25 [-0.34, -0.16]
Rapoport et al. 2000 IG1	10	BES	31	14 (9)	8 (7)		2.09%	-0.73 [-0.94, -0.52]
Rapoport et al. 2000 IG2	10	BES	31	15 (9)	6 (5)		1.98%	-1.22 [-1.49, -0.94]
Raynor et al. 2006 IG1 & IG2	9	BES	15	16.3 (7.7)	11.6 (7)		1.95%	-0.62 [-0.91, -0.33]
Rieger et al. 2017 IG1	52	BES	58	18.1 (8.11)	11.41 (7.34)		2.19%	-0.85 [-0.99, -0.71]
Rieger et al. 2017 IG2	52	BES	54	17.01 (7.67)	10.37 (6.28)		2.18%	-0.92 [-1.07, -0.77]
Smith et al. 2018 IG1.1	36	BES	14	16.94 (8.25)	10.57 (7.23)		1.91%	-0.79 [-1.11, -0.48]
Smith et al. 2018 IG2.1	36	BES	11	12.66 (7.42)	7.27 (5.81)		1.88%	-0.76 [-1.09, -0.43]
Wadden et al. 1993 IG1	52	BES	17	22.88 (8.18)	12 (6.78)		1.68%	-1.41 [-1.84, -0.99]
Wadden et al. 1993 IG2	52	BES	23	23.46 (7.27)	18.32 (8.18)		2.05%	-0.65 [-0.89, -0.42]
Zwickert et al. 2016 IG1	60	BES	31	18.9 (7.79)	13.4 (10.58)		2.12%	-0.58 [-0.78, -0.39]
Zwickert et al. 2016 IG2	60	BES	29	18.1 (8.08)	13.1 (10.77)		2.12%	-0.52 [-0.71, -0.32]
RE Model for Subgroup (Q = 588.75, df = 35, p = 0.00; I^2 = 94.5%)								-0.70 [-0.82, -0.58]
**Difference scores**								
Cheng et al. 2014 IG1	51.43	BES	21			-5.38 (6.83)	2.09%	-0.35 [-0.56, -0.13]
Cheng et al. 2014 IG2	51.43	BES	15			-3.93 (8.06)	2.04%	-0.21 [-0.46, 0.03]
Fogelholm et al. 1999 IG1	12	BITE	26			-2.5 (3.57)	2.13%	-0.31 [-0.50, -0.12]
Fogelholm et al. 1999 IG2	12	BITE	26			-1.2 (3.06)	2.14%	-0.17 [-0.35, 0.01]
Fogelholm et al. 1999 IG3	12	BITE	26			-3.4 (4.59)	2.13%	-0.33 [-0.51, -0.14]
Keranen et al. 2009 IG1	77.14	BES	20			-7 (6.85)	2.06%	-0.45 [-0.68, -0.22]
Keranen et al. 2009 IG2	77.14	BES	29			-5 (6.87)	2.14%	-0.32 [-0.50, -0.14]
Whitelock et al. 2019 IG1	8	BES	53			-1.3 (5.7)	2.21%	-0.10 [-0.22, 0.02]
Whitelock et al. 2019 IG2	8	BES	54			-2.3 (5.8)	2.21%	-0.18 [-0.30, -0.05]
Martin et al. 2019 IG1	24	MAEDS	59			-1.97 (6.31)	2.21%	-0.14 [-0.26, -0.02]
Martin et al. 2019 IG2	24	MAEDS	51			-3.42 (12.44)	2.20%	-0.12 [-0.25, 0.00]
Martin et al. 2019 IG3	24	MAEDS	61			-3.07 (6.24)	2.21%	-0.22 [-0.34, -0.10]
RE Model for Subgroup (Q = 16.51, df = 11, p = 0.12; I^2 = 31.9%)								-0.21 [-0.27, -0.16]
Test for Subgroup Differences: Q_M = 18.28, df = 1, p = 0.00, Pooled I^2 = 93.0							100.00%	-0.58 [-0.69, -0.47]

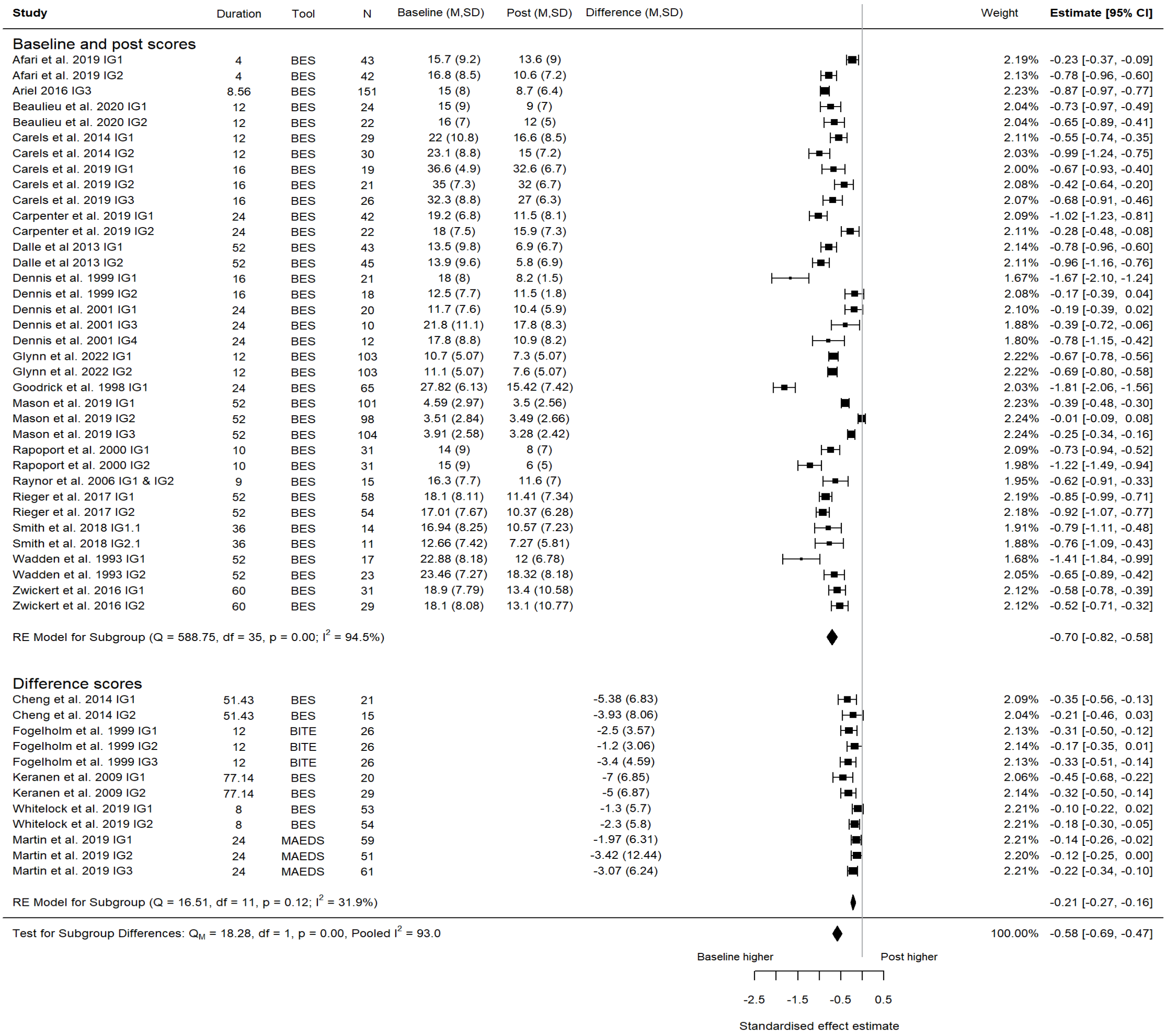

Figure S19: Binge eating [Baseline - Post] assuming a correlation of 0.9
Forest plot of the change in binge eating from baseline-post for each trial. Each estimate was standardized using Hedges g. A correlation of 0.9 was assumed between time points when necessary for the calculation of Hedges' g. A random effects model was used to combine estimates from each trial (Prediction lower bound: -1.31, Prediction upper bound: 0.15, Tau^2: 0.1344).

Study	Duration	Tool	N	Baseline (M,SD)	Post (M,SD)	Difference (M,SD)	Weight	Estimate [95% CI]
**Minimal intervention or usual care**								
Martin et al. 2019 IG3	24	MAEDS	61			-3.07 (6.24)	2.29%	-0.49 [-0.75, -0.23]
Whitelock et al. 2019 IG2	8	BES	54			-2.3 (5.8)	2.26%	-0.39 [-0.67, -0.12]
Keranen et al. 2009 IG2	77.14	BES	29			-5 (6.87)	1.92%	-0.72 [-1.11, -0.32]
Dennis et al. 1999 IG2	16	BES	18	12.5 (7.7)	11.5 (1.8)		1.98%	-0.17 [-0.55, 0.20]
RE Model for Subgroup (Q = 4.07, df = 3, p = 0.25; I^2 = 0.0%)								-0.44 [-0.59, -0.28]
**Full intervention**								
Martin et al. 2019 IG2	24	MAEDS	51			-3.42 (12.44)	2.24%	-0.27 [-0.55, 0.01]
Martin et al. 2019 IG1	24	MAEDS	59			-1.97 (6.31)	2.29%	-0.31 [-0.57, -0.05]
Whitelock et al. 2019 IG1	8	BES	53			-1.3 (5.7)	2.26%	-0.23 [-0.50, 0.05]
Keranen et al. 2009 IG1	77.14	BES	20			-7 (6.85)	1.61%	-1.00 [-1.51, -0.49]
Fogelholm et al. 1999 IG3	12	BITE	26			-3.4 (4.59)	1.85%	-0.73 [-1.15, -0.31]
Fogelholm et al. 1999 IG2	12	BITE	26			-1.2 (3.06)	1.90%	-0.39 [-0.79, 0.02]
Fogelholm et al. 1999 IG1	12	BITE	26			-2.5 (3.57)	1.86%	-0.69 [-1.11, -0.27]
Cheng et al. 2014 IG2	51.43	BES	15			-3.93 (8.06)	1.52%	-0.47 [-1.02, 0.07]
Cheng et al. 2014 IG1	51.43	BES	21			-5.38 (6.83)	1.70%	-0.77 [-1.25, -0.30]
Zwickert et al. 2016 IG2	60	BES	29	18.1 (8.08)	13.1 (10.77)		2.18%	-0.52 [-0.82, -0.21]
Zwickert et al. 2016 IG1	60	BES	31	18.9 (7.79)	13.4 (10.58)		2.19%	-0.58 [-0.88, -0.29]
Wadden et al. 1993 IG2	52	BES	23	23.46 (7.27)	18.32 (8.18)		2.03%	-0.65 [-1.01, -0.30]
Wadden et al. 1993 IG1	52	BES	17	22.88 (8.18)	12 (6.78)		1.56%	-1.41 [-1.94, -0.88]
Smith et al. 2018 IG2.1	36	BES	11	12.66 (7.42)	7.27 (5.81)		1.69%	-0.76 [-1.24, -0.28]
Smith et al. 2018 IG1.1	36	BES	14	16.94 (8.25)	10.57 (7.23)		1.77%	-0.79 [-1.25, -0.34]
Rieger et al. 2017 IG2	52	BES	54	17.01 (7.67)	10.37 (6.28)		2.42%	-0.92 [-1.13, -0.71]
Rieger et al. 2017 IG1	52	BES	58	18.1 (8.11)	11.41 (7.34)		2.44%	-0.85 [-1.05, -0.65]
Raynor et al. 2006 IG1 & IG2	9	BES	15	16.3 (7.7)	11.6 (7)		1.78%	-0.62 [-1.07, -0.18]
Rapoport et al. 2000 IG2	10	BES	31	15 (9)	6 (5)		2.04%	-1.22 [-1.57, -0.86]
Rapoport et al. 2000 IG1	10	BES	31	14 (9)	8 (7)		2.16%	-0.73 [-1.04, -0.43]
Mason et al. 2019 IG3	52	BES	104	3.91 (2.58)	3.28 (2.42)		2.55%	-0.25 [-0.40, -0.10]
Mason et al. 2019 IG2	52	BES	98	3.51 (2.84)	3.49 (2.66)		2.55%	-0.01 [-0.16, 0.14]
Mason et al. 2019 IG1	52	BES	101	4.59 (2.97)	3.5 (2.56)		2.55%	-0.39 [-0.54, -0.24]
Goodrick et al. 1998 IG1	24	BES	65	27.82 (6.13)	15.42 (7.42)		2.20%	-1.81 [-2.11, -1.52]
Glynn et al. 2022 IG2	12	BES	103	11.1 (5.07)	7.6 (5.07)		2.52%	-0.69 [-0.85, -0.52]
Glynn et al. 2022 IG1	12	BES	103	10.7 (5.07)	7.3 (5.07)		2.52%	-0.67 [-0.83, -0.50]
Dennis et al. 2001 IG4	24	BES	12	17.8 (8.8)	10.9 (8.2)		1.57%	-0.78 [-1.31, -0.26]
Dennis et al. 2001 IG3	24	BES	10	21.8 (11.1)	17.8 (8.3)		1.55%	-0.39 [-0.92, 0.14]
Dennis et al. 2001 IG1	24	BES	20	11.7 (7.6)	10.4 (5.9)		2.04%	-0.19 [-0.54, 0.17]
Dennis et al. 1999 IG1	16	BES	21	18 (8)	8.2 (1.5)		1.61%	-1.67 [-2.18, -1.16]
Dalle et al 2013 IG2	52	BES	45	13.9 (9.6)	5.8 (6.9)		2.27%	-0.96 [-1.23, -0.69]
Dalle et al 2013 IG1	52	BES	43	13.5 (9.8)	6.9 (6.7)		2.29%	-0.78 [-1.04, -0.52]
Carpenter et al. 2019 IG2	24	BES	22	18 (7.5)	15.9 (7.3)		2.08%	-0.28 [-0.62, 0.06]
Carpenter et al. 2019 IG1	24	BES	42	19.2 (6.8)	11.5 (8.1)		2.23%	-1.02 [-1.31, -0.73]
Carels et al. 2019 IG3	16	BES	26	32.3 (8.8)	27 (6.3)		2.09%	-0.68 [-1.02, -0.35]
Carels et al. 2019 IG2	16	BES	21	35 (7.3)	32 (6.7)		2.03%	-0.42 [-0.77, -0.07]
Carels et al. 2019 IG1	16	BES	19	36.6 (4.9)	32.6 (6.7)		1.92%	-0.67 [-1.06, -0.27]
Carels et al. 2014 IG2	12	BES	30	23.1 (8.8)	15 (7.2)		2.08%	-0.99 [-1.33, -0.66]
Carels et al. 2014 IG1	12	BES	29	22 (10.8)	16.6 (8.5)		2.17%	-0.55 [-0.85, -0.24]
Beaulieu et al. 2020 IG2	12	BES	22	16 (7)	12 (5)		2.01%	-0.65 [-1.01, -0.28]
Beaulieu et al. 2020 IG1	12	BES	24	15 (9)	9 (7)		2.04%	-0.73 [-1.09, -0.38]
Ariel 2016 IG3	8.56	BES	151	15 (8)	8.7 (6.4)		2.56%	-0.87 [-1.01, -0.73]
Afari et al. 2019 IG2	4	BES	42	16.8 (8.5)	10.6 (7.2)		2.28%	-0.78 [-1.05, -0.51]
Afari et al. 2019 IG1	4	BES	43	15.7 (9.2)	13.6 (9)		2.35%	-0.23 [-0.47, 0.01]
RE Model for Subgroup (Q = 291.03, df = 43, p = 0.00; I^2 = 84.6%)								-0.68 [-0.78, -0.57]
Test for Subgroup Differences: Q_M = 2.31, df = 1, p = 0.13, Pooled I^2 = 83.2							100.00%	-0.66 [-0.76, -0.55]

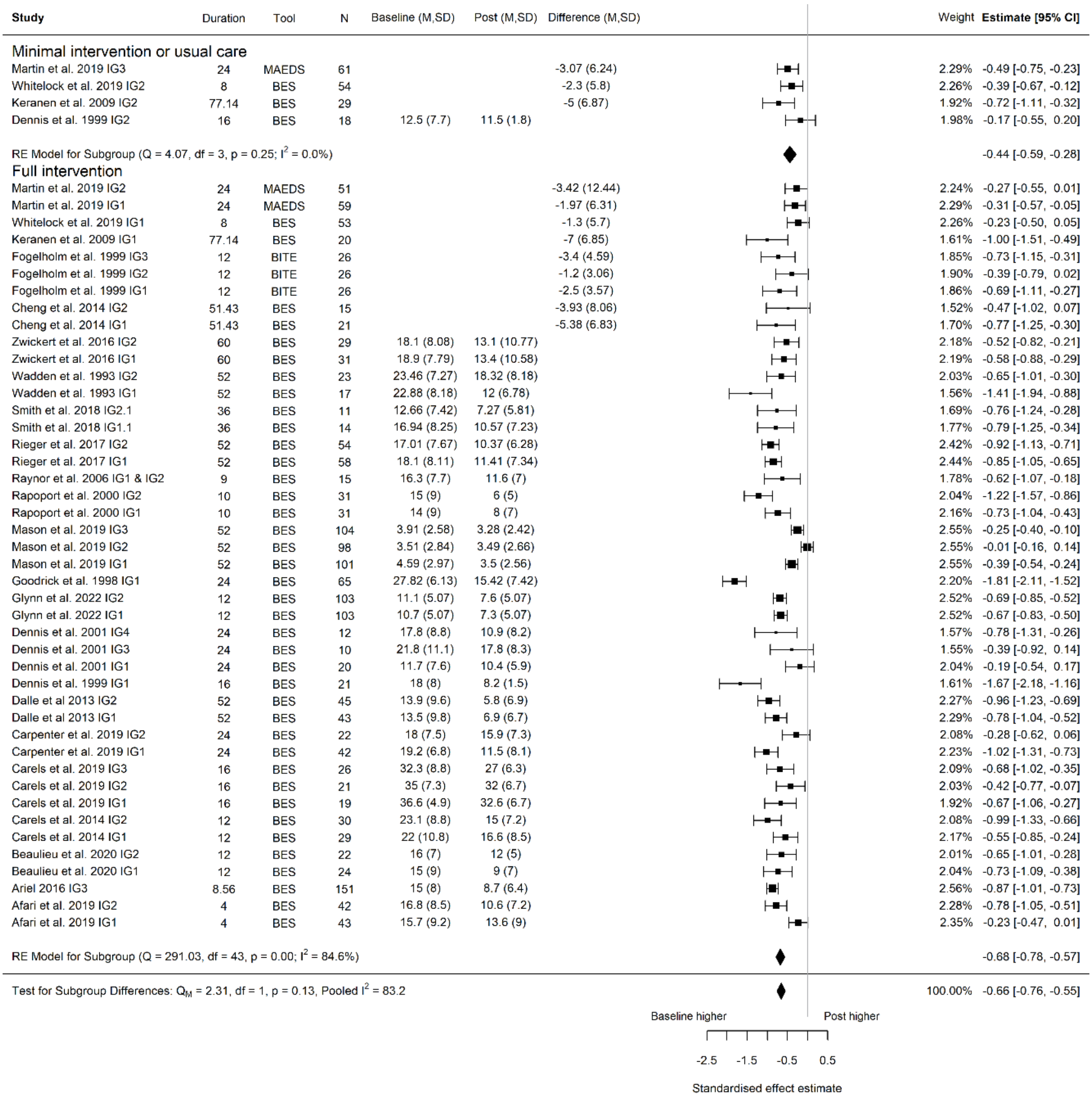

Figure S20. Forest plot of the change in binge eating from baseline to post for each trial split into the subgroups minimal or full intervention.

Each estimate was standardized using Hedges' g. A correlation of 0.7 was assumed between time points when necessary for the calculation of Hedges' g. A random effects model was used to combine estimates from each trial.

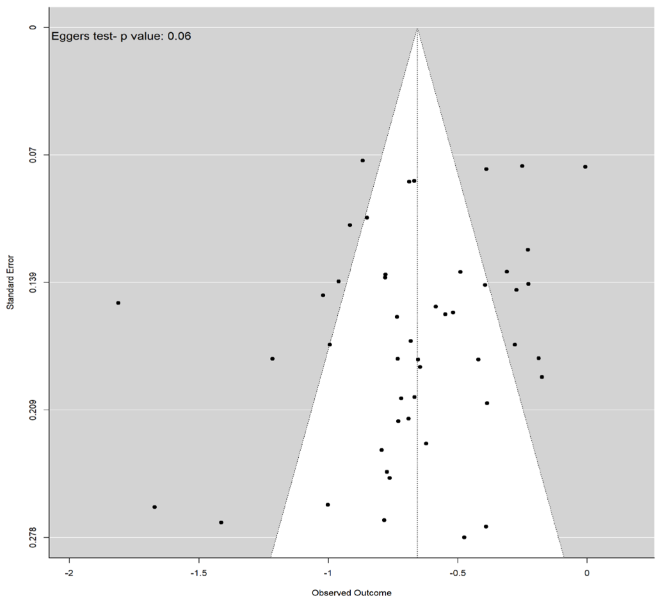

Figure S21: Binge eating [Baseline - Post] funnel plot
Funnel plot with the standardized change (Hedges' g) in binge eating between baseline and post on the x axis and standard error on the y axis.

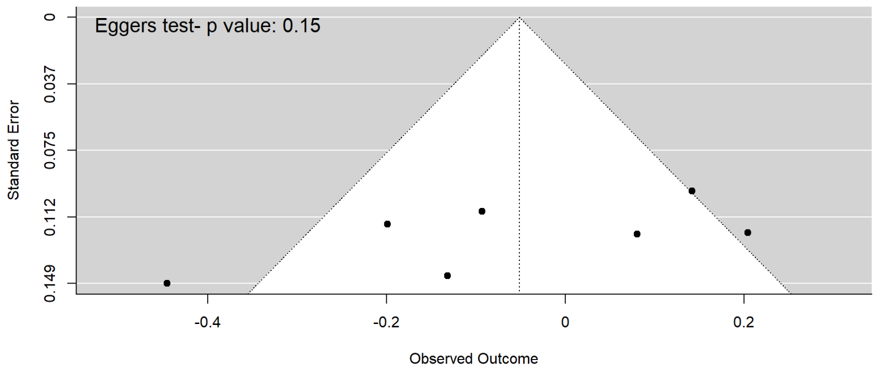

Figure S22: Binge eating [Post - Follow-up] funnel plot
Funnel plot with the standardized change (Hedges' g) in binge eating between post and follow-up on the x axis and standard error on the y axis.

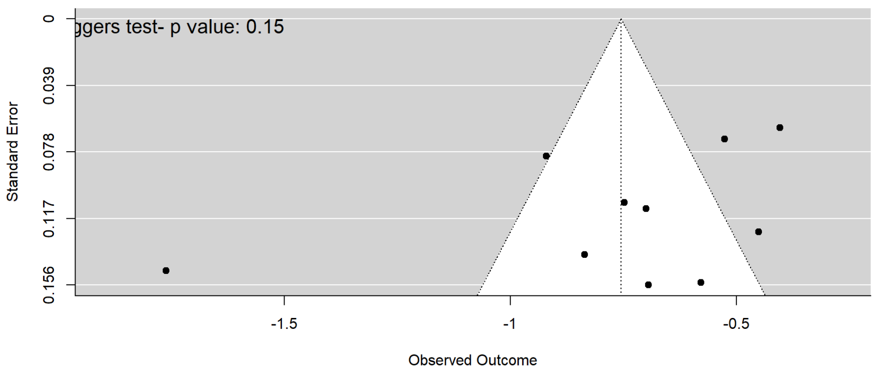

Figure S23: Binge eating [Baseline - Follow-up] funnel plot
Funnel plot with the standardized change (Hedges' g) in binge eating between baseline and follow-up on the x axis and standard error on the y axis.

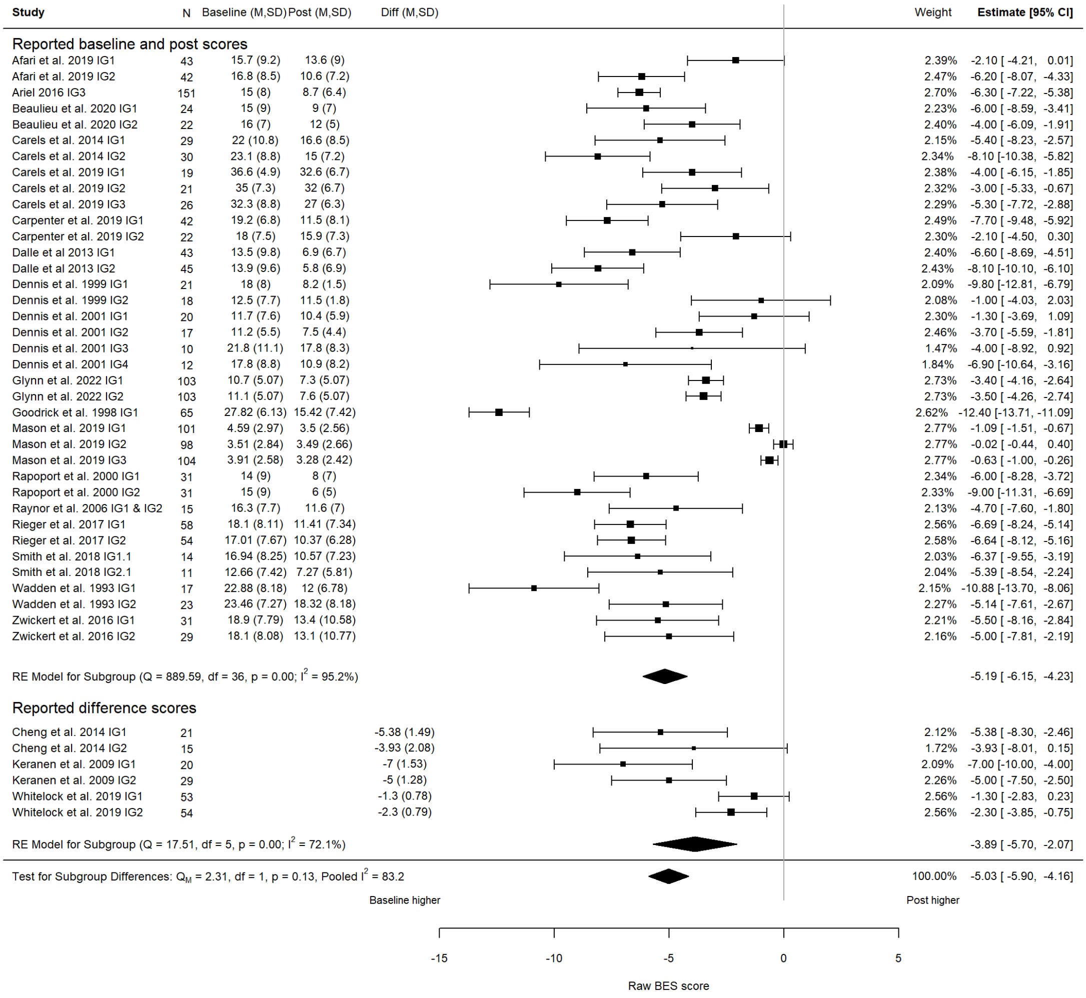

Figure S24: BES scale only [Baseline - Post] forest plot

Forest plot that only includes measurements of binge eating that have been assessed with the BES tool. Raw scores were used to calculate a mean difference between baseline and post for each trial and a random effects model was used to combine estimates from each trial.

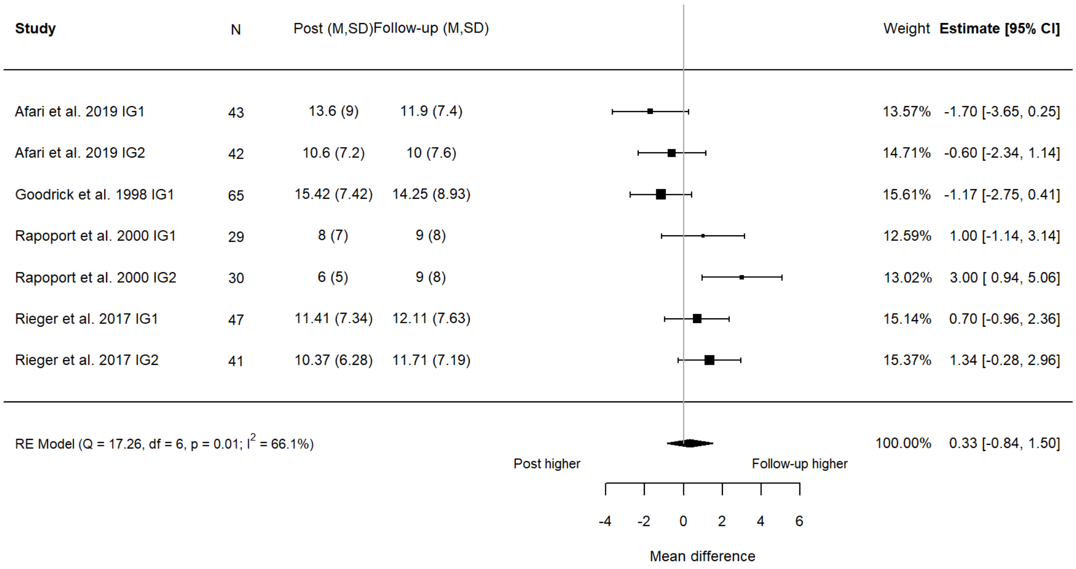

Figure S25: BES scale only [Post - Follow-up] forest plot
Forest plot that only includes measurements of binge eating that have been assessed with the BES tool. Raw scores were used to calculate a mean difference between post and follow-up for each trial and a random effects model was used to combine estimates from each trial.

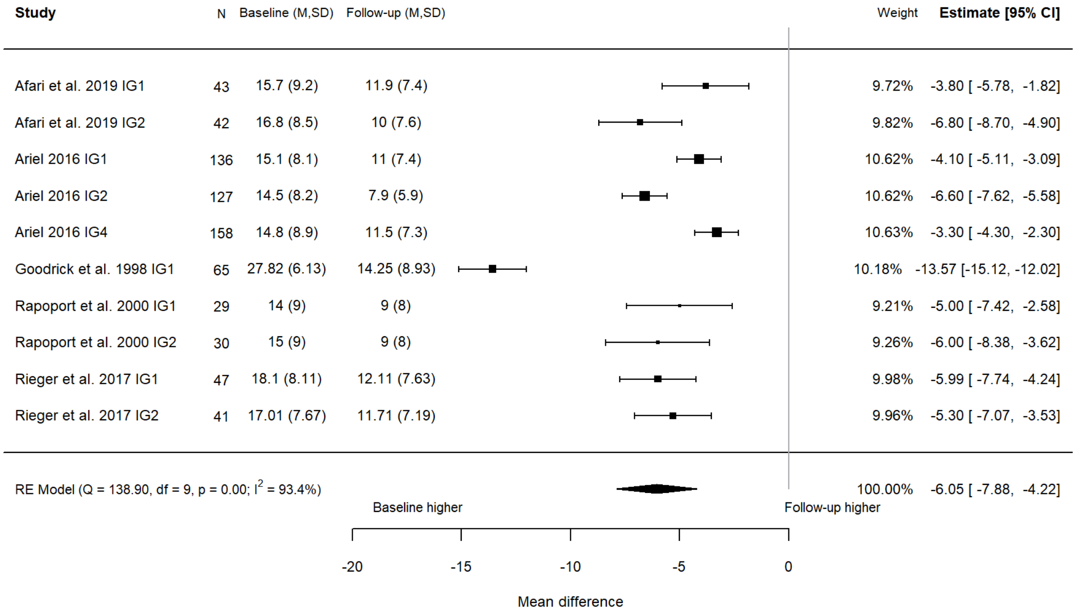

Figure S26. BES scale only [Baseline - Follow-up] forest plot
Forest plot that only includes measurements of binge eating that have been assessed with the BES tool. Raw scores were used to calculate a mean difference between baseline and follow-up for each trial and a random effects model was used to combine estimates from each trial.

References

1. Dietetics AoNa. Evidence Analysis Manual. Chicago: Academy of Nutrition and Dietetics, 2016.
2. Afari N, Herbert MS, Godfrey KM, et al. Acceptance and commitment therapy as an adjunct to the MOVE! programme: a randomized controlled trial. Obes Sci Pract 2019;5(5):397-407. (In eng). DOI: 10.1002/osp4.356.
3. Ariel AH, Perri MG. Effect of dose of behavioral treatment for obesity on binge eating severity. Eating Behaviors 2016;22:55-61. DOI: https://doi.org/10.1016/j.eatbeh.2016.03.032.
4. Bacon L, Keim NL, Van Loan MD, et al. Evaluating a 'non-diet' wellness intervention for improvement of metabolic fitness, psychological well-being and eating and activity behaviors. Int J Obes Relat Metab Disord 2002;26(6):854-65. (In eng). DOI: 10.1038/sj.ijo.0802012.
5. Bacon L, Stern JS, Van Loan MD, Keim NL. Size acceptance and intuitive eating improve health for obese, female chronic dieters. J Am Diet Assoc 2005;105(6):929-36. (In eng). DOI: 10.1016/j.jada.2005.03.011.
6. Barnes RD, Ivezaj V, Martino S, Pittman BP, Grilo CM. Back to Basics? No Weight Loss from Motivational Interviewing Compared to Nutrition Psychoeducation at One-Year Follow-Up. Obesity (Silver Spring) 2017;25(12):2074-2078. (In eng). DOI: 10.1002/oby.21972.
7. Barnes RD, White MA, Martino S, Grilo CM. A randomized controlled trial comparing scalable weight loss treatments in primary care. Obesity 2014;22(12):2508-2516. DOI: https://doi.org/10.1002/oby.20889.
8. Beaulieu K, Casanova N, Oustric P, et al. Matched Weight Loss Through Intermittent or Continuous Energy Restriction Does Not Lead To Compensatory Increases in Appetite and Eating Behavior in a Randomized Controlled Trial in Women with Overweight and Obesity. The Journal of Nutrition 2019;150(3):623-633. DOI: 10.1093/jn/nxz296.
9. Bolognese MA, Franco CB, Ferrari A, et al. Group Nutrition Counseling or Individualized Prescription for Women With Obesity? A Clinical Trial. Front Public Health 2020;8:127. (In eng). DOI: 10.3389/fpubh.2020.00127.
10. Carels RA, Young KM, Koball A, et al. Transforming your life: an environmental modification approach to weight loss. J Health Psychol 2011;16(3):430-8. (In eng). DOI: 10.1177/1359105310380986.
11. Carels RA, Burmeister JM, Koball AM, et al. A randomized trial comparing two approaches to weight loss: differences in weight loss maintenance. J Health Psychol 2014;19(2):296-311. (In eng). DOI: 10.1177/1359105312470156.
12. Carels RA, Caroline Miller J, Selensky JC, et al. Using an acceptance-based behavioral approach as a supplement to obesity treatment: A stepped-care approach. Journal of Contextual Behavioral Science 2019;12:98-105. DOI: https://doi.org/10.1016/j.jcbs.2019.03.002.
13. Carpenter KM, Vickerman KA, Salmon EE, Javitz HS, Epel ES, Lovejoy JC. A Randomized Pilot Study of a Phone-Based Mindfulness and Weight Loss Program. Behavioral Medicine 2019;45(4):271-281. DOI: 10.1080/08964289.2017.1384359.
14. Cheng HL, Griffin H, Claes B-E, et al. Influence of dietary macronutrient composition on eating behaviour and self-perception in young women undergoing weight management. Eating and Weight Disorders - Studies on Anorexia, Bulimia and Obesity 2014;19(2):241-247. DOI: 10.1007/s40519-014-0110-y.
15. Christaki E, Kokkinos A, Costarelli V, Alexopoulos EC, Chrousos GP, Darviri C. Stress management can facilitate weight loss in Greek overweight and obese women: a pilot study. Journal of Human Nutrition and Dietetics 2013;26(s1):132-139. DOI: https://doi.org/10.1111/jhn.12086.
16. Cooper Z, Doll HA, Hawker DM, et al. Testing a new cognitive behavioural treatment for obesity: A randomized controlled trial with three-year follow-up. Behaviour Research and Therapy 2010;48(8):706-713. DOI: https://doi.org/10.1016/j.brat.2010.03.008.
17. Dalle Grave R, Calugi S, Gavasso I, El Ghoch M, Marchesini G. A randomized trial of energy-restricted high-protein versus high-carbohydrate, low-fat diet in morbid obesity. Obesity 2013;21(9):1774-1781. DOI: https://doi.org/10.1002/oby.20320.

18. Dassen FCM, Houben K, Van Breukelen GJP, Jansen A. Gamified working memory training in overweight individuals reduces food intake but not body weight. Appetite 2018;124:89-98. DOI: https://doi.org/10.1016/j.appet.2017.05.009.
19. Dennis KE, Tomoyasu N, McCrone SH, Goldberg AP, Bunyard L, Bing Qi B. Self-Efficacy Targeted Treatments for Weight Loss in Postmenopausal Women. Sch Inq Nurs Pract 2001(3):259-276. (In eng). DOI: 10.1891/0889-7182.15.3.259.
20. Dennis KE, Pane KW, Adams BK, Qi BB. The Impact of a Shipboard Weight Control Program. Obesity Research 1999;7(1):60-67. DOI: https://doi.org/10.1002/j.1550-8528.1999.tb00391.x.
21. DiMarco ID, Klein DA, Clark VL, Wilson GT. The use of motivational interviewing techniques to enhance the efficacy of guided self-help behavioral weight loss treatment. Eat Behav 2009;10(2):134-6. (In eng). DOI: 10.1016/j.eatbeh.2009.02.001.
22. Fogelholm M, Kukkonen-Harjula K, Oja P. Eating control and physical activity as determinants of short-term weight maintenance after a very-low-calorie diet among obese women. International Journal of Obesity 1999;23(2):203-210. DOI: 10.1038/sj.ijo.0800825.
23. Glynn EL, Fleming SA, Edwards CG, Wilson MJ, Evans M, Leidy HJ. Consuming a Protein and Fiber-Based Supplement Preload Promotes Weight Loss and Alters Metabolic Markers in Overweight Adults in a 12-Week, Randomized, Double-Blind, Placebo-Controlled Trial. J Nutr 2022;152(6):1415-1425. (In eng). DOI: 10.1093/jn/nxac038.
24. Goodrick GK, Poston WS, 2nd, Kimball KT, Reeves RS, Foreyt JP. Nondieting versus dieting treatment for overweight binge-eating women. J Consult Clin Psychol 1998;66(2):363-8. (In eng). DOI: 10.1037//0022-006x.66.2.363.
25. Jeffery RW, Wing RR, Thorson C, Burton LR. Use of personal trainers and financial incentives to increase exercise in a behavioral weight-loss program. J Consult Clin Psychol 1998;66(5):777-83. (In eng). DOI: 10.1037//0022-006x.66.5.777.
26. Jospe MR, Roy M, Brown RC, et al. The Effect of Different Types of Monitoring Strategies on Weight Loss: A Randomized Controlled Trial. Obesity (Silver Spring) 2017;25(9):1490-1498. (In eng). DOI: 10.1002/oby.21898.
27. Kalarchian MA, Marcus MD, Courcoulas AP, Cheng Y, Levine MD. Preoperative lifestyle intervention in bariatric surgery: initial results from a randomized, controlled trial. Obesity (Silver Spring) 2013;21(2):254-60. (In eng). DOI: 10.1002/oby.20069.
28. Keränen A-M, Savolainen MJ, Reponen AH, et al. The effect of eating behavior on weight loss and maintenance during a lifestyle intervention. Preventive Medicine 2009;49(1):32-38. DOI: https://doi.org/10.1016/j.ypmed.2009.04.011.
29. Raynor HA, Steeves EA, Hecht J, Fava JL, Wing RR. Limiting variety in non-nutrient-dense, energy-dense foods during a lifestyle intervention: a randomized controlled trial. Am J Clin Nutr 2012;95(6):1305-14. (In eng). DOI: 10.3945/ajcn.111.031153.
30. LaRose JG, Fava JL, Steeves EA, Hecht J, Wing RR, Raynor HA. Daily self-weighing within a lifestyle intervention: impact on disordered eating symptoms. Health Psychol 2014;33(3):297-300. (In eng). DOI: 10.1037/a0034218.
31. Foster-Schubert KE, Alfano CM, Duggan CR, et al. Effect of Diet and Exercise, Alone or Combined, on Weight and Body Composition in Overweight-to-Obese Postmenopausal Women. Obesity 2012;20(8):1628-1638. DOI: https://doi.org/10.1038/oby.2011.76.
32. Mason C, de Dieu Tapsoba J, Duggan C, Wang CY, Alfano CM, McTiernan A. Eating behaviors and weight loss outcomes in a 12-month randomized trial of diet and/or exercise intervention in postmenopausal women. Int J Behav Nutr Phys Act 2019;16(1):113. (In eng). DOI: 10.1186/s12966-019-0887-1.
33. Martin CK, Johnson WD, Myers CA, et al. Effect of different doses of supervised exercise on food intake, metabolism, and non-exercise physical activity: The E-MECHANIC randomized controlled trial. Am J Clin Nutr 2019;110(3):583-592. (In eng). DOI: 10.1093/ajcn/nqz054.

34. Myers CA, Johnson WD, Earnest CP, et al. Examination of mechanisms (E-MECHANIC) of exercise-induced weight compensation: study protocol for a randomized controlled trial. Trials 2014;15(1):212. DOI: 10.1186/1745-6215-15-212.
35. Mensinger JL, Calogero RM, Tylka TL. Internalized weight stigma moderates eating behavior outcomes in women with high BMI participating in a healthy living program. Appetite 2016;102:32-43. DOI: https://doi.org/10.1016/j.appet.2016.01.033.
36. Moss EL, Tobin LN, Campbell TS, von Ranson KM. Behavioral weight-loss treatment plus motivational interviewing versus attention control: lessons learned from a randomized controlled trial. Trials 2017;18(1):351. (In eng). DOI: 10.1186/s13063-017-2094-1.
37. Muggia C, Falchi AG, Michelini I, et al. Brief group cognitive behavioral treatment in addition to prescriptive diet versus standard care in obese and overweight patients. A randomized controlled trial. e-SPEN Journal 2014;9(1):e26-e33. DOI: https://doi.org/10.1016/j.clnme.2013.11.002.
38. Sherwood NE, Crain AL, Martinson BC, et al. Keep it off: a phone-based intervention for long-term weight-loss maintenance. Contemp Clin Trials 2011;32(4):551-60. (In eng). DOI: 10.1016/j.cct.2011.03.011.
39. Pacanowski CR, Senso MM, Oriogun K, Crain AL, Sherwood NE. Binge eating behavior and weight loss maintenance over a 2-year period. J Obes 2014;2014:249315. (In eng). DOI: 10.1155/2014/249315.
40. Radin RM, Epel ES, Daubenmier J, et al. Do stress eating or compulsive eating influence metabolic health in a mindfulness-based weight loss intervention? Health Psychol 2020;39(2):147-158. (In eng). DOI: 10.1037/hea0000807.
41. Daubenmier J, Moran PJ, Kristeller J, et al. Effects of a mindfulness-based weight loss intervention in adults with obesity: A randomized clinical trial. Obesity (Silver Spring) 2016;24(4):794-804. (In eng). DOI: 10.1002/oby.21396.
42. Raman J, Hay P, Tchanturia K, Smith E. A randomised controlled trial of manualized cognitive remediation therapy in adult obesity. Appetite 2018;123:269-279. (In eng). DOI: 10.1016/j.appet.2017.12.023.
43. Ramirez EM, Rosen JC. A comparison of weight control and weight control plus body image therapy for obese men and women. J Consult Clin Psychol 2001;69(3):440-6. (In eng). DOI: 10.1037//0022-006x.69.3.440.
44. Rapoport L, Clark M, Wardle J. Evaluation of a modified cognitive-behavioural programme for weight management. Int J Obes Relat Metab Disord 2000;24(12):1726-37. (In eng). DOI: 10.1038/sj.ijo.0801465.
45. Raynor HA, Niemeier HM, Wing RR. Effect of limiting snack food variety on long-term sensory-specific satiety and monotony during obesity treatment. Eating Behaviors 2006;7(1):1-14. DOI: https://doi.org/10.1016/j.eatbeh.2005.05.005.
46. Rieger E, Treasure J, Murray K, Caterson I. The use of support people to improve the weight-related and psychological outcomes of adults with obesity: A randomised controlled trial. Behaviour Research and Therapy 2017;94:48-59. DOI: https://doi.org/10.1016/j.brat.2017.04.012.
47. Rieger E, Treasure J, Swinbourne J, Adam B, Manns C, Caterson I. The effectiveness of including support people in a cognitive behavioural weight loss maintenance programme for obese adults: study rationale and design. Clinical Obesity 2014;4(2):77-90. DOI: https://doi.org/10.1111/cob.12042.
48. Schyns G, van den Akker K, Roefs A, Houben K, Jansen A. Exposure therapy vs lifestyle intervention to reduce food cue reactivity and binge eating in obesity: A pilot study. Journal of Behavior Therapy and Experimental Psychiatry 2020;67:101453. DOI: https://doi.org/10.1016/j.jbtep.2019.01.005.
49. van den Akker K, Schyns G, Jansen A. Enhancing inhibitory learning to reduce overeating: Design and rationale of a cue exposure therapy trial in overweight and obese women. Contemporary Clinical Trials 2016;49:85-91. DOI: https://doi.org/10.1016/j.cct.2016.06.008.

50. Simpson SA, McNamara R, Shaw C, et al. A feasibility randomised controlled trial of a motivational interviewing-based intervention for weight loss maintenance in adults. Health Technol Assess 2015;19(50):v-vi, xix-xxv, 1-378. (In eng). DOI: 10.3310/hta19500.
51. Smith BW, Shelley BM, Sloan AL, Colleran KM, Erickson K. A Preliminary Randomized Controlled Trial of a Mindful Eating Intervention for Post-menopausal Obese Women. Mindfulness 2018;9:836-849.
52. Steinberg DM, Tate DF, Bennett GG, Ennett S, Samuel-Hodge C, Ward DS. Daily self-weighing and adverse psychological outcomes: a randomized controlled trial. Am J Prev Med 2014;46(1):24-9. (In eng). DOI: 10.1016/j.amepre.2013.08.006.
53. Tanco S, Linden W, Earle T. Well-being and morbid obesity in women: A controlled therapy evaluation. International Journal of Eating Disorders 1998;23(3):325-339. DOI: https://doi.org/10.1002/(SICI)1098-108X(199804)23:3<325::AID-EAT10>3.0.CO;2-X.
54. Vander Wal JS, Waller SM, Klurfeld DM, et al. Effect of a post-dinner snack and partial meal replacement program on weight loss. Int J Food Sci Nutr 2006;57(1-2):97-106. (In eng). DOI: 10.1080/09637480600658369.
55. Wadden TA, Foster GD, Letizia KA. One-year behavioral treatment of obesity: comparison of moderate and severe caloric restriction and the effects of weight maintenance therapy. J Consult Clin Psychol 1994;62(1):165-71. (In eng). DOI: 10.1037//0022-006x.62.1.165.
56. Wadden TA, Foster GD, Sarwer DB, et al. Dieting and the development of eating disorders in obese women: results of a randomized controlled trial. Am J Clin Nutr 2004;80(3):560-8. (In eng). DOI: 10.1093/ajcn/80.3.560.
57. Werrij MQ, Jansen A, Mulkens S, Elgersma HJ, Ament AJ, Hospers HJ. Adding cognitive therapy to dietetic treatment is associated with less relapse in obesity. J Psychosom Res 2009;67(4):315-24. (In eng). DOI: 10.1016/j.jpsychores.2008.12.011.
58. Whitelock V, Kersbergen I, Higgs S, Aveyard P, Halford JCG, Robinson E. A smartphone based attentive eating intervention for energy intake and weight loss: results from a randomised controlled trial. BMC Public Health 2019;19(1):611. (In eng). DOI: 10.1186/s12889-019-6923-x.
59. Williamson DA, Martin CK, Anton SD, et al. Is caloric restriction associated with development of eating-disorder symptoms? Results from the CALERIE trial. Health Psychol 2008;27(1s):S32-42. (In eng). DOI: 10.1037/0278-6133.27.1.S32.
60. Zwickert K, Rieger E, Swinbourne J, et al. High or low intensity text-messaging combined with group treatment equally promote weight loss maintenance in obese adults. Obesity Research & Clinical Practice 2016;10(6):680-691. DOI: https://doi.org/10.1016/j.orcp.2016.01.001.
61. Wooldridge JS, Herbert MS, Hernandez J, et al. Improvement in 6-min Walk Test Distance Following Treatment for Behavioral Weight Loss and Disinhibited Eating: an Exploratory Secondary Analysis. Int J Behav Med 2019;26(4):443-448. (In eng). DOI: 10.1007/s12529-019-09796-1.
62. Barnes RD, White MA, Martino S, Grilo CM. A randomized controlled trial comparing scalable weight loss treatments in primary care. Obesity (Silver Spring) 2014;22(12):2508-16. (In eng). DOI: 10.1002/oby.20889.
63. Jospe MR, Brown RC, Williams SM, Roy M, Meredith-Jones KA, Taylor RW. Self-monitoring has no adverse effect on disordered eating in adults seeking treatment for obesity. Obes Sci Pract 2018;4(3):283-288. (In eng). DOI: 10.1002/osp4.168.
